# AXL-associated tumor inflammation as a poor prognostic signature in chemotherapy-treated triple-negative breast cancer patients

**DOI:** 10.1038/npjbcancer.2016.33

**Published:** 2016-11-02

**Authors:** Giulia Bottai, Carlotta Raschioni, Borbála Székely, Luca Di Tommaso, Attila M Szász, Agnese Losurdo, Balázs Győrffy, Balázs Ács, Rosalba Torrisi, Niki Karachaliou, Tímea Tőkés, Michele Caruso, Janina Kulka, Massimo Roncalli, Armando Santoro, Alberto Mantovani, Rafael Rosell, Jorge S Reis-Filho, Libero Santarpia

**Affiliations:** 1 Oncology Experimental Therapeutics, IRCCS Humanitas Clinical and Research Center, Milan, Italy; 2 2nd Department of Pathology, Semmelweis University, Budapest, Hungary; 3 Department of Pathology, IRCCS Humanitas Clinical and Research Center, Milan, Italy; 4 Department of Oncology and Hematology, IRCCS Humanitas Cancer Center, Milan, Italy; 5 MTA TTK Momentum Cancer Biomarker Research Group, Hungarian Academy of Sciences, Budapest, Hungary; 6 2nd Department of Pediatrics, Semmelweis University, Budapest, Hungary; 7 Cancer Biology and Precision Medicine Program, Catalan Institute of Oncology, Hospital Germans Trias i Pujol, Barcelona, Spain; 8 Department of Medical Oncology, Humanitas Oncology Center of Catania, Catania, Italy; 9 Humanitas University, Milan, Italy; 10 Department of Immunology and Inflammation, IRCCS Humanitas Clinical and Research Center, Milan, Italy; 11 Germans Trias i Pujol Health Sciences Institute and Hospital, Campus Can Ruti, Barcelona, Spain; 12 Department of Pathology, Memorial Sloan-Kettering Cancer Center, New York, NY, USA

## Abstract

A subgroup of triple-negative breast cancer (TNBC) shows epithelial-to-mesenchymal transition (EMT) features, which are sustained by the interaction between cancer cells and tumor-associated macrophages (TAMs). In this study, the clinical relevance of 30 EMT-related kinases and the potential cross-talk with TAMs were investigated in a cohort of 203 TNBC patients treated with adjuvant chemotherapy. The prognostic value of the evaluated markers was validated in two independent cohorts of TNBC patients treated with adjuvant chemotherapy (*N*=95; *N*=137). *In vitro*, we investigated the potential synergism between cancer cells and TAMs. We found that the EMT-related kinase AXL showed the highest correlation with the frequency of CD163-positive macrophages (*r*_S_=0.503; *P*<0.0001). Relapsing TNBC patients presented high expression of AXL (*P*<0.0001) and CD163 (*P*<0.018), but only AXL retained independent prognostic significance in multivariate analysis (relapse-free survival, *P*=0.002; overall survival *P*=0.001). *In vitro* analysis demonstrated that *AXL*-expressing TNBC cells were able to polarize human macrophages towards an M2-like phenotype, and modulate a specific pattern of pro-tumor cytokines and chemokines. Selective AXL inhibition impaired the activity of M2-like macrophages, reducing cancer cell invasiveness, and restoring the sensitivity of breast cancer cells to chemotherapeutic drugs. These data suggest that the EMT-related kinase AXL overexpressed in cancer cells has prognostic significance, and contributes to the functional skewing of macrophage functions in TNBC. AXL inhibition may represent a novel strategy to target cancer cells, as well as tumor-promoting TAMs in TNBC.

## Introduction

Triple-negative breast cancer (TNBC) is clinically defined by the lack of expression of estrogen and progesterone receptors, and no overexpression/amplification of HER2. This subgroup of tumors is usually characterized by an aggressive phenotype associated with an increased risk of early recurrence with distant metastasis to visceral organs and poor prognosis.^1,2^ Burgeoning evidence demonstrates that TNBC is a very heterogeneous disease, encompassing different molecular entities.^3,4^ Current treatment approaches are limited to cytotoxic chemotherapy due to the lack of specific therapeutic targets.^4,5^ Even though TNBC patients are initially responsive to chemotherapy, the long-term results are not satisfactory.^1,2,4^ Therefore, the identification of reliable prognostic markers in chemotherapy-treated patients, as well as novel targetable signaling pathways, may allow a better stratification of TNBC patients into different risk groups and the development of novel treatment strategies.

A subgroup of TNBC shows epithelial-to-mesenchymal transition (EMT) features, leading to tumor progression and resistance to chemotherapy.^1–5^ Early tumor recurrences, driven by chemotherapy-resistant cancer cells, represent a prominent cause of poor outcome in TNBC. In this context, EMT allows tumor cells to avoid apoptosis and cellular senescence, increasing tumor cell invasive properties and drug resistance.^1,6–8^

Growing evidence indicates that EMT is induced by different biological mechanisms, and that the mutual interaction between cancer cells and the surrounding microenvironment is a crucial step supporting the entire EMT process.^9,10^ Tumor cells that undergo EMT regulate the phenotype and the activity of non-malignant stromal cells, particularly tumor-associated macrophages (TAMs), contributing to cancer progression and metastatic spread.^11,12^ TAMs are able to affect the behavior of malignant cells, and to modulate the functions of tumor-infiltrating lymphocytes, overall promoting tumor progression and influencing therapy response.^12–19^ In particular, stromal TAMs with pro-tumor functions, resembling the phenotype of M2-polarized macrophages, have been shown to be capable of inducing EMT, ultimately establishing a positive local feedback loop that sustains cancer cells invasiveness, indirectly influencing the response to chemotherapy.^10–19^ Conversely, conventional chemotherapy has been demonstrated to contribute to the modulation of the tumor microenvironment by inducing the recruitment of TAMs at the tumor site and by reducing therapy efficacy in breast cancer patients.^15–20^

Even though the interaction between cancer cells with mesenchymal traits and TAMs is emerging as a crucial factor in tumor progression and response to therapy, the clinical relevance of this cross-talk in TNBC is still poorly understood. In this study, we first evaluated the expression of several kinases involved in EMT in a large cohort of TNBC patients treated with anthracycline–taxane-based adjuvant chemotherapy. Subsequently, we assessed the clinical relevance of the most significant EMT-related kinase, the receptor AXL, validating its prognostic and functional role in additional independent cohorts of TNBC and by *in vitro* assays. Finally, we evaluated the correlation between AXL and TAMs, investigating specific mechanisms by which TNBC cells and macrophages cooperate to influence tumor progression and response to therapy.

## Results

### The receptor tyrosine kinase AXL is positively associated with macrophage infiltration in TNBC

To evaluate the potential relationship between cancer cells with mesenchymal features and the presence of TAMs in breast tumor stroma, we selected 30 relevant EMT-related kinases and correlated their expression with the expression of the pan-macrophage marker CD68 in a cohort of 203 TNBC patients (Supplementary Tables 1 and 2). We found that *AXL* was the most significant kinase correlated with the frequency of CD68-positive TAMs in the internal cohort of 203 TNBC patients (Spearman’s coefficient, *r*_S_=0.405; Bonferroni-adjusted *P*=0.007; Supplementary Figure 1a; Supplementary Table 2). We confirmed this positive correlation also at the protein level in the same cohort (rs=0.342; *P*<0.0001; Supplementary Figure 1b), and by analyzing gene expression data from three publicly available TNBC data sets (*N*=311; *r*_S_=0.360; *P*<0.0001; Supplementary Figure 1c; Supplementary Table 1).

**Figure 1:**
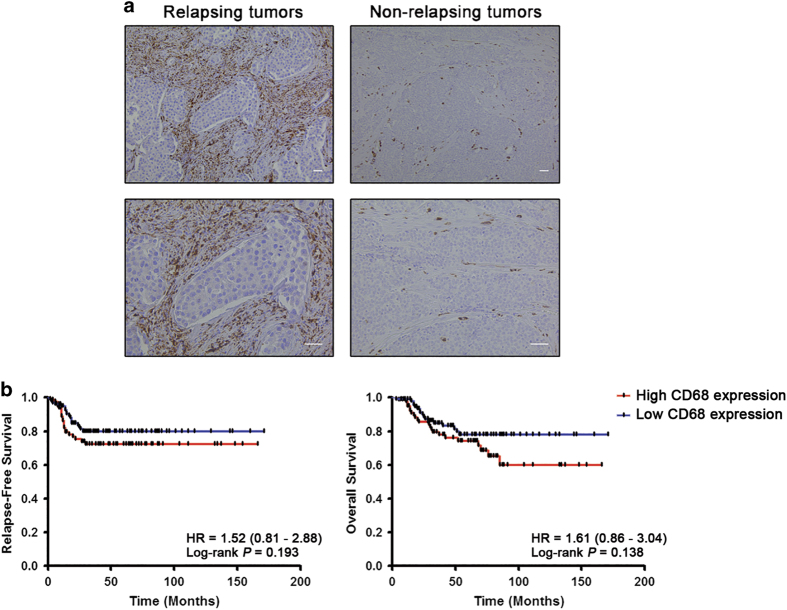
High infiltration of CD68-positive cells is associated with tumor relapse but not with survival in triple-negative breast cancer. (**a**) Representative immunohistochemical staining of CD68 in tumor samples from TNBC patients with recurrence (left panel) and without recurrence (right panel). Scale bars represent 50 μm. (**b**) Kaplan–Meier analysis for relapse-free survival (left panel) and overall survival (right panel) according to the content of CD68-positive cells in tumor stroma. TNBC patients (*N*=203) were stratified by absent/moderate (0–2) or dense (3) macrophage infiltration. Curves were compared using log-rank test. *P* values and HR (95% CI in parentheses) are shown. CI, confidence interval; HR, hazard ratio; TNBC, triple-negative breast cancer.

Furthermore, considering the potential association between *AXL* expression and tumor immune response, we evaluated the clinical significance of global macrophage content in the internal cohort of TNBC patients. We found a higher content of CD68-positive macrophages in TNBC patients who experienced recurrence within 36 months after surgery compared with non-recurrent patients (55.3% vs. 38.5%, *P*=0.045; Figure 1a; Table 1). No statistically significant association between the presence of CD68-positive macrophages and other clinicopathological parameters was, however, identified (Table 1). Furthermore, Kaplan–Meier analysis showed no prognostic relevance of CD68-positive macrophage count in terms of relapse-free survival (RFS) and overall survival (OS) in TNBC patients (Figure 1b).

### AXL correlates with M2-polarized macrophages and is an independent prognostic marker in TNBC

To evaluate the potential role of AXL in the regulation of cancer-related inflammation in TNBC, we analyzed co-regulated genes and pathways using the SEEK platform and the Ingenuity Pathway Analysis software. This analysis demonstrated that, besides its well-known role in EMT, *AXL* is strongly co-expressed with genes involved in several immune functions (Supplementary Figure 2a; Supplementary Table 3), confirming AXL as a key factor of a gene network that influence both EMT and tumor-associated inflammation in TNBC.

Given the plasticity and heterogeneity of macrophages, we next investigated the role of AXL in shaping the inflammatory tumor microenvironment in TNBC. Two distinct immune gene modules reflecting the polarization of anti-tumor M1 or pro-tumor M2 macrophages were assembled based on a literature review and analyzed using SEEK (Supplementary Figure 2b,c and Supplementary Table 4). We found that *AXL* was consistently co-expressed with genes enclosed in the M2-related module (co-expression score=1.013; *P*=0.013), whereas it showed no relationship with M1-polarized macrophages in TNBC (Supplementary Figure 2b,c and Supplementary Table 5), suggesting that AXL expressed by tumor cells may be involved in the switch towards an M2 phenotype, thus sustaining the pro-tumor activity of TAMs.

**Figure 2:**
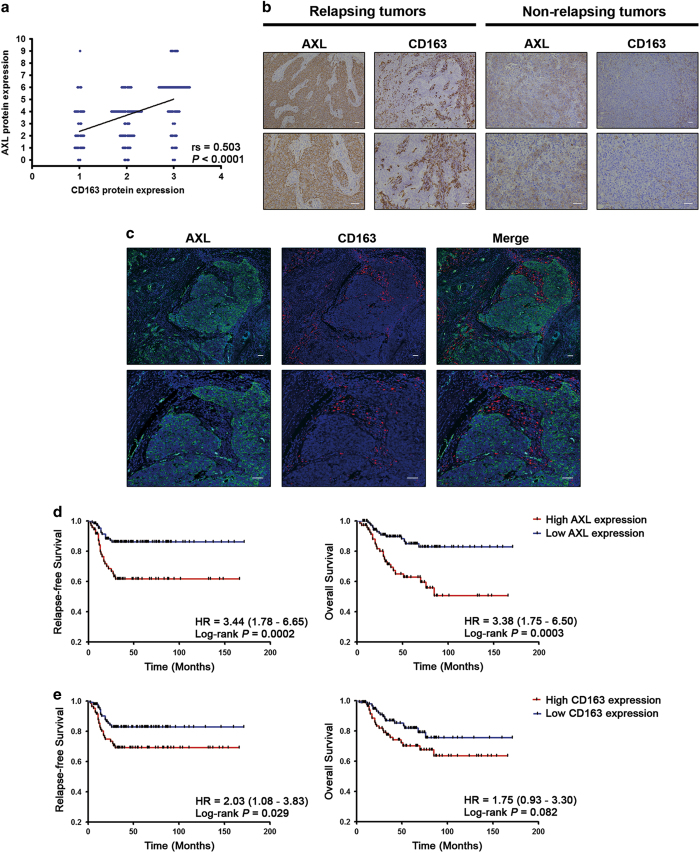
AXL expression correlates with the infiltration of CD163-positive cells in tumor stroma and is associated with survival in triple-negative breast cancer. (**a**) A scatter diagram shows a positive correlation between immunohistochemical staining of AXL and CD163 in TNBC (*N*=203; Spearman’s coefficient, *r*_S_=0.503; *P*<0.0001). (**b**) Representative immunohistochemical staining of AXL and CD163 in tumor samples of serial sections from TNBC patients with recurrence (left panel) and without recurrence (right panel). Scale bars represent 50 μm. (**c**) Representative pictures of double immunofluorescent staining and confocal microscopy of TNBC sections, showing that AXL-expressing cancer cells (green) are in close contact with adjacent stromal TAMs (red). Scale bars represent 50 μm. (**d**) Kaplan–Meier analysis for relapse-free survival (left panel) and overall survival (right panel) according to AXL immunohistochemical score. TNBC patients (*N*=203) were stratified by low (0–4) or high (6–9) AXL expression. (**e**) Kaplan–Meier analysis for relapse-free survival (left panel) and overall survival (right panel) according to the content of CD163-positive cells in tumor stroma. TNBC patients (*N*=203) were stratified by absent/moderate (0–2) or dense (3) CD163-positive macrophage infiltration. Curves were compared using log-rank test. *P* values and HR (95% CI in parentheses) are shown. CI, confidence interval; HR, hazard ratio; TNBC, triple-negative breast cancer.

To confirm these observations, we analyzed the expression of AXL and the M2 macrophage marker CD163 in the internal cohort of 203 TNBC patients, which revealed a positive correlation between AXL and the infiltration of CD163-positive cells (*r*_S_=0.503; *P*<0.0001; Figure 2a). Furthermore, TNBC patients who experienced distant relapse had a significant higher expression of both AXL and CD163 compared with patients without recurrence (*P*<0.0001; *P*=0.018, respectively; Figure 2b and c; Table 1). High levels of AXL expression were also associated with lymph node positivity (*P*=0.042), and interestingly with metastasis to visceral organs as the first sites of distant recurrence (*P*=0.036; Table 1).

To assess the prognostic value of AXL and CD163 in TNBC patients, we performed Kaplan–Meier and Cox univariate regression analyses in the internal cohort of TNBC patients (*N*=203; Figure 2d,e and Supplementary Table 6). We found that patients with high levels of AXL protein expression had significant shorter RFS and OS (log-rank *P*=0.0002; hazard ratio (HR)=3.44; 95% confidence interval (CI), 1.78–6.65 for RFS; log-rank *P*=0.0003; HR=3.38; 95% CI, 1.75–6.50 for OS; Figure 2c and Supplementary Table 6), whereas CD163 was associated with RFS only (log-rank *P*=0.029; HR=2.03; 95% CI, 1.08–3.83; Figure 2d and Supplementary Table 6). Cox multivariate regression analysis, adjusted for age at diagnosis, histological grade, lymph node status, tumor size, and tumor stage, demonstrated that only AXL expression remained an independent poor prognostic factor for RFS and OS in TNBC patients treated with adjuvant chemotherapy, along with lymph node status, whereas CD163 only retained a positive trend for reduced RFS (Table 2). Multivariate Cox analysis performed in an additional independent cohort of 95 chemotherapy-treated TNBC patients, and also analyzing AXL as a continuous variable, confirmed the prognostic significance of AXL for both RFS and OS (Supplementary Table 7 and Supplementary Table 8).

We next assessed the prognostic value of *AXL* by analyzing the gene expression data from 137 TNBC patients treated with adjuvant chemotherapy (Supplementary Table 1). We confirmed that *AXL* expression was consistently associated with reduced RFS (log-rank *P*=0.008; HR=2.3; 95% CI, 1.20–4.30; Supplementary Figure 3a). We also found that *AXL* expression did not correlate with expression levels of the proliferation marker *MKI67* (Pearson’s coefficient, *r*=0.014; Supplementary Figure 3b).

### *AXL*-overexpressing breast cancer cells and M2-like macrophages reciprocally interact *in vitro*

**Figure 3:**
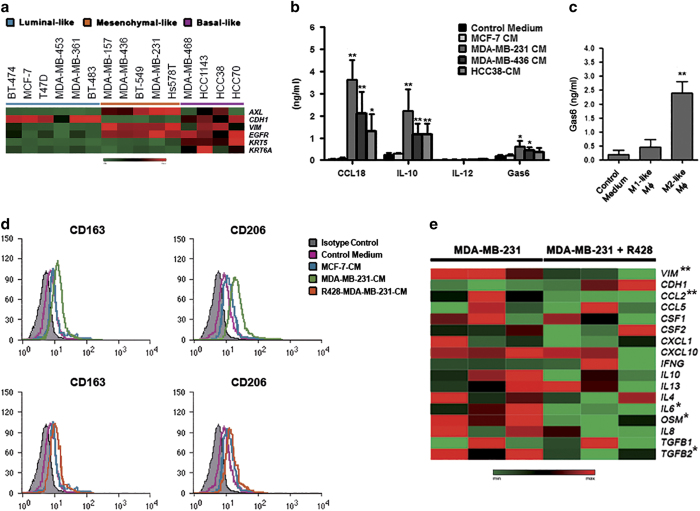
*AXL*-overexpressing breast cancer cells promote the polarization of M2 macrophages. (**a**) Expression analysis of *AXL, EMT *(*CDH1* and *VIM*), and basal (*EGFR*, *KRT5*, and *KRT6A*) markers in 15 breast cancer cell lines by qRT-PCR. Gene expression levels were visualized in a heatmap. (**b**) Cytokine levels in the media of macrophages cultured in the absence or presence of MCF-7- or TNBC cell-derived conditioned medium (CM) measured by ELISA. *P* values were obtained using a two-tailed Student’s *t*-test (mean±s.d., *N*=4 experiments; *P<0.05; **P⩽0.01). (**c**) ELISA analysis of Gas6 in the medium of *in vitro* polarized human macrophages (Mφ). *P* values were obtained using a two-tailed Student’s *t*-test (mean±s.d., *N*=4 experiments; **P⩽0.01). (**d**) Flow cytometric analysis of the M2 markers CD163 and CD206 in human monocytes cultured in the absence (pink) or presence of MCF-7-CM (blue), MDA-MB-231-CM (green; upper panel), or R428-treated MDA-MB-231-CM (orange; lower panel) for 6 days. Gray histograms represent staining with isotype controls. The histograms are representatives of five independent experiments. (**e**) Heatmap showing the effect of R428 on the expression of EMT markers and relevant cytokines/chemokines in MDA-MB-231 cells (three biological replicates were shown). Significant genes were indicated with an asterisks (*P<0.05; **P⩽0.01). ELISA, enzyme-linked immunosorbent assay; EMT, epithelial-to-mesenchymal transition; TNBC, triple-negative breast cancer.

To characterize the expression of *AXL in vitro*, we analyzed the expression of the tyrosine kinase, EMT (*CDH1* and *VIM*), and basal (*EGFR*, *KRT5*, and *KRT6A*) markers in 15 breast cancer cell lines by quantitative real-time PCR (qRT-PCR; Figure 3a). We demonstrated that *AXL* expression was higher in all triple-negative mesenchymal-like breast cancer cell lines, compared with luminal/epithelial cells (Figure 3a). High levels of *AXL* expression were also found in two triple-negative basal-like cells (Figure 3a).

Then, we investigated the biological mechanisms underlying the cross-talk between *AXL*-expressing breast cancer cells and TAMs, and its impact on tumor progression and anticancer drug resistance. To identify the soluble factors potentially mediating the interaction between macrophages and breast cancer cells, we measured the release of several major cytokines/chemokines by macrophages exposed to the conditioned medium (CM) derived from the *AXL*-expressing breast cancer cell lines HCC38, MDA-MB-231, and MDA-MB-436, and from the *AXL*-negative MCF-7 cells. We found that the medium of macrophages treated with the CM derived from *AXL*-expressing cells, especially from mesenchymal-like cells, was enriched for tumor-promoting mediators, including CCL18, and IL-10, compared with that from macrophages treated with MCF-7-CM (Figure 3b). Conversely, the presence of *AXL*-expressing cells did not affect the release of IL-12, which is commonly associated with the M1 phenotype (Figure 3b). Mesenchymal-like cells also induced an increased production of the AXL ligand Gas6 (*P*<0.05; Figure 3b), and a positive trend was also observed for basal-like cells, although not reaching statistical significance (Figure 3b). Consistently, we found that Gas6 was significantly released from *in vitro* polarized M2-like macrophages, but not from M1-like cells (*P*=0.014; Figure 3c). These results may suggest that the interaction between M2 TAMs and mesenchymal TNBC cells could be partially modulated by the AXL/Gas6 signaling. To further investigate the ability of *AXL*-expressing TNBC cells to promote macrophage polarization towards a pro-tumor phenotype, we analyzed the expression of the M2 specific markers CD206 and CD163 by flow cytometry (Figure 3d). *AXL*-expressing mesenchymal-like TNBC cells, but not MCF-7, were able to polarize macrophages towards an M2 phenotype (Figure 3d, upper panel). We also found that *AXL*-expressing basal-like cells can induce these phenotypic changes in macrophages, although to a lesser extent than mesenchymal-like cells, whereas the polarizing effect of *AXL*-negative basal-like cells was marginal (Supplementary Figure 4), suggesting that AXL may sustain the cancer inflammation cross-talk beyond its primary role in EMT. Noteworthy, the capability of MDA-MB-231 cells to change the expression of these surface markers was impaired by the selective inhibition of AXL with R428 (Figure 3d, lower panel). Consistently, we found that R428-treated MDA-MB-231 cells showed reduced expression of vimentin (*P*=0.008), *CCL2* (*P*=0.008), *IL6* (*P*=0.024), *oncostatin M* (*OSM*, *P*=0.019), and *TGFB2* (*P*=0.029) as compared with untreated cells (Figure 3e), indicating that AXL may contribute to the recruitment, and the polarization of macrophages into M2 cells by increasing the release of specific cytokines and chemokines.

**Figure 4:**
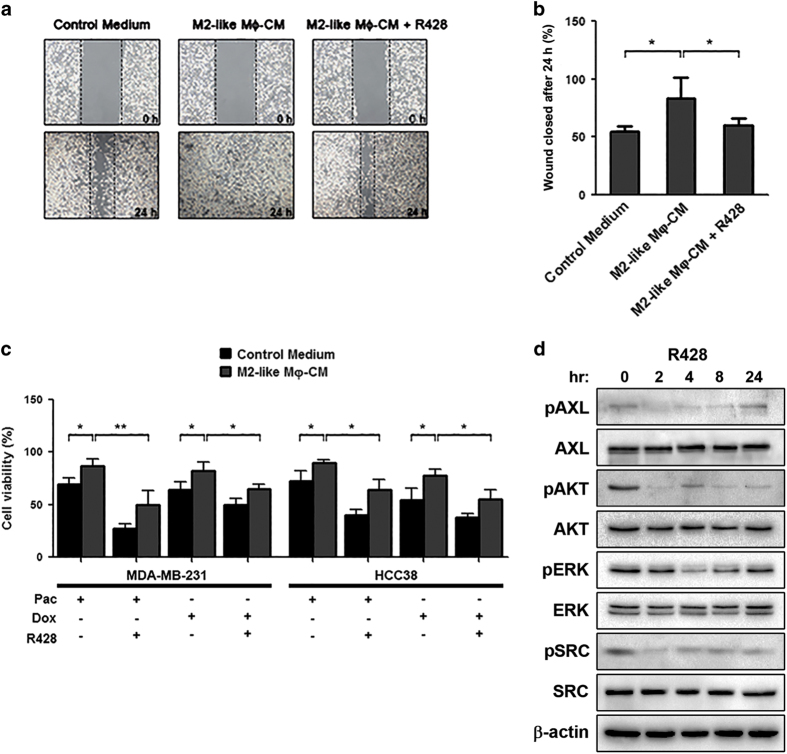
M2-polarized macrophages sustain tumor aggressiveness and influence drug sensitivity of *AXL*-overexpressing breast cancer cells. (**a**) Wound-healing assays were conducted with untreated or R428-treated MDA-MB-231 cells cultivated in the absence (control medium) or presence of conditioned medium (CM) derived from *in vitro* polarized M2 macrophages. (**b**) Statistical analysis of wound closure. Gap size at 0 h was set to 100% and percentage of closed wound was calculated after 24 h. (**c**) M2-polarized macrophages increase the resistance of HCC38 and MDA-MB-231 cells to paclitaxel (Pac) and doxorubicin (Dox) treatments compared with cells treated with control medium, whereas the selective inhibition of AXL with R428 restores drug sensitivity in TNBC cells. (**d**) Western blot with the indicated antibodies of lysates from MDA-MB-231 cells treated with 1 μmol/l R428 at different time points. β-Actin was used as a loading control. All *P *values were obtained using a two-tailed Student’s *t*-test (mean±s.d., *N*=3 experiments; *P<0.05; **P⩽0.01).

Afterwards, we evaluated the relevance of the reciprocal cross-talk between M2 TAMs and cancer cells for tumor progression and chemotherapy response. We demonstrated that the CM from M2-polarized macrophages enhanced MDA-MB-231 cell migration (*P*=0.020; Figure 4a,b), and increased the resistance of MDA-MB-231 and HCC38 TNBC cells to paclitaxel (*P*=0.028; *P*=0.039, respectively) and doxorubicin (*P*=0.043; *P*=0.029, respectively; Figure 4c). Importantly, AXL inhibition with R428 reduced the migratory capacity (*P*=0.036, Figure 4a,b) and restored the sensitivity of MDA-MB-231 and HCC38 cells to paclitaxel (*P*=0.012; *P*=0.019, respectively) and doxorubicin (*P*=0.027; *P*=0.020, respectively; Figure 4c). In addition to AXL inhibition, the treatment with R428 affected the activation of other oncogenic pathways, including AKT, ERK1/2, and SRC signaling, suggesting possible cross-talks and escape mechanisms to AXL inhibition in TNBC (Figure 4d). Collectively, these data suggest that the interaction between M2 TAMs and TNBC cells through AXL has an important role in supporting tumor progression and chemoresistance.

## Discussion

The tumor microenvironment is of paramount importance in breast cancer progression, and accumulating evidence indicates an emerging role of the cross-talk signaling between mesenchymal cancer cells and TAMs. In fact, EMT and TAMs provide invasive and metastatic capabilities to tumor cells and modulate the tumor microenvironment, leading to the suppression of anti-cancer immune response, and limiting the effects of cytotoxic chemotherapy.^6–11^ TNBC, which is often characterized by the presence of both EMT and TAMs, is a good model to explore potential molecular markers maintaining the biological intersections between these two signalings.^9–12^ In this study, we demonstrated that the receptor AXL was the most significant EMT-related kinase associated with macrophage cells in the tumor stroma of TNBC. This receptor is emerging as an important effector of the EMT program, and its activation is responsible for triggering important oncogenic pathways, such as PI3K/AKT/mTOR, NF-κB, EGFR, and MAPK cascades, involved in cell proliferation, survival, and invasion.^21–26^ In line with different studies on human cancer, we found that the increased expression of AXL significantly correlated with poor outcome in TNBC.^21–28^ High levels of AXL were associated with reduced RFS and OS in TNBC patients treated with anthracycline–taxane-based adjuvant chemotherapy. Interestingly, AXL overexpression was associated with distant tumor recurrence, particularly to visceral organs, indicating a specific route of tumor dissemination for a subset of these tumors. *AXL* has been previously shown to be one of the most differentially expressed genes in the mesenchymal stem-like subtype compared with other subgroups of TNBC.^29^ Our data demonstrated that the expression of *AXL* was a specific feature of TNBC cells, especially those with mesenchymal features, sustaining the key role of AXL in the activation of the EMT program, and in mediating cancer cell aggressiveness.^21–24,30^ Furthermore, we showed that AXL was involved in the modulation of several immune pathways, including leukocyte migration and chemotaxis, macrophage activation, and agranulocyte adhesion and diapedesis, further supporting the biological relevance of the interaction between *AXL*-expressing cancer cells and cancer-related immune responses in TNBC.^9–15^

Previous studies reported that CD68-positive TAMs are a potential prognostic marker in breast cancer.^17,22,31,32^ However, we found that CD68-positive macrophages in tumor stroma mildly correlated with tumor relapse and were not significantly associated with TNBC patients outcome. Therefore, given potential differences in the evaluation of protein expression among studies, the clinical significance and utility of CD68 expression in TNBC remains uncertain. These data also highlight the concept that CD68 probably does not accurately reflect the presence and function of distinct macrophage subpopulations within the tumor stroma, at least in breast cancer.^18,33,34^ Consequently, our results advocate the importance to further evaluate the biological role and functions of different macrophage subpopulations in breast cancer. Indeed, macrophages exhibit remarkable functional and phenotypic plasticity, with activated M2-like cells displaying tumor-promoting activities.^14,22^ Accordingly, most human tumors exhibit TAMs with an M2-like phenotype, promoting EMT, and contributing to tumor progression and drug resistance.^11–20,35^ In agreement with these reports, we found that the massive presence of M2 macrophages correlated with an aggressive behavior of TNBC. Moreover, *AXL* was significantly co-expressed with genes associated with M2 macrophages, and positively correlated with the infiltration of CD163-positive M2 cells, suggesting an important relationship between *AXL*-expressing cells and TAMs with pro-tumor activity in a subgroup of TNBC.

The cross-talk between mesenchymal cancer cells and tumor microenvironment is recognized as a key factor in tumor progression in several tumors.^10–12,14,30^ However, we demonstrated that CD163-positive M2 TAMs did not retain their prognostic significance in multivariate analysis. Our data suggest that the CD163-positive embedded stromal cells likely have a role in sustaining the aggressiveness of cancer cells, but the presence of pro-tumor macrophages alone may not be sufficient to affect the outcome of TNBC patients. Although the mechanisms underlying the link between cancer cells and TAMs are complex and difficult to dissect *in vitro*, we observed the reciprocal nature of this interaction sustained by AXL. Our data show that *AXL*-positive TNBC cells with mesenchymal traits activate human macrophages to an M2-like phenotype, modulating a specific pattern of pro-tumor cytokines and chemokines. Indeed, mesenchymal-like cells were able to increase the release of CCL18 and IL-10, which are known macrophage-derived mediators of metastatic dissemination and resistance to chemotherapy in breast cancer.^36,37^ Furthermore, we demonstrated that the selective inhibition of AXL with R428 impaired the ability of mesenchymal cells to induce the polarization of macrophages, by reducing the release of CCL2, IL-6, oncostatin M, and TGF-β, which are well-known inducers of the M2 phenotype.^13,38^ In addition, AXL may be also involved in the recruitment of macrophages at the tumor site, increasing the production of CCL2 that functions as a monocyte chemoattractant protein.^39^ Noteworthy, we found that *AXL*-expressing mesenchymal-like cells were also capable of inducing the release of the AXL ligand Gas6, which is selectively secreted by M2-type macrophages. Our results suggest that the AXL/Gas6 signaling may have a role in modulating the interaction between mesenchymal TNBC cells and M2 TAMs, and support previous findings, indicating that tumor cells induced infiltrating TAMs to increase the production of Gas6, promoting cell growth and metastasis in different cancer models.^40^ Even though AXL can be activated by Gas6, alternative ligand-independent mechanisms, including the interaction with EGFR, which is frequently express in TNBC, have been demonstrated.^1,24–26^ Therefore, although the biological modalities of AXL action should be further investigated, our results suggest that TNBC cells with mesenchymal features may ‘educate’ infiltrating TAMs to support tumor progression, and that targeting AXL may be a novel strategy to reduce both EMT and the pro-tumor activity of TAMs in TNBC.

Finally, we demonstrated that the presence of M2-polarized macrophages enhanced the migratory potential and chemoresistance of TNBC cells, whereas the selective pharmacological inhibition of AXL was able to drastically reduce cell aggressiveness, and to restore response to chemotherapeutic drugs, including taxanes and anthracyclines. These results are in agreement with recent findings showing that infiltrating macrophages reduced the primary breast tumor drug response, and that R428 enhanced the efficacy of anti-mitotic drugs in mesenchymal-like lung and breast cancer cells.^37,41^ Noteworthy, AXL inhibition also affected the activation of other oncogenic pathways, providing the evidence of cross-talk signaling between different pathways and the activation of compensatory feedback networks. We showed that *AXL* was also expressed in basal-like breast cancer cells. Even though the effect of *AXL*-expressing basal-like cells on macrophage polarization was mild compared with that of mesenchymal-like cells, TAMs were equally able to induce chemoresistance in both TNBC models. These findings suggest that, beyond the role of mesenchymal-like cells in supporting the cross-talk with TAMs, AXL inhibition could be a potential therapeutic strategy for a broader range of patients with *AXL*-expressing TNBC.

Our study has some limitations. Even though we provide evidence of the involvement of AXL kinase in macrophage polarization, the specific requirement of AXL for the interaction with TAMs, the identification of the soluble mediators of this cross-talk, and the potentially distinct biological role of different subtypes of TNBC cells warrant further studies. Given the retrospective nature of this study, and to determine the potential heterogeneity of treatment effects associated with AXL functions, our findings would need to be further validated in a prospective clinical trial. Despite these limitations, our results suggest that AXL is a prognostic indicator of outcome in TNBC treated with chemotherapy. Furthermore, AXL supports the pro-tumor activity of M2-type macrophages, inducing tumor progression and resistance to chemotherapy. Overall, our data provide support for the use of AXL targeted therapy to reduce tumor aggressiveness, overcome chemotherapy resistance, and impair the cross-talk between cancer cells and TAMs, in patients with TNBC overexpressing AXL.

## Materials and Methods

### Patients’ cohorts and tumor samples

Formalin-fixed, paraffin-embedded (FFPE) tissues were retrospectively collected from 203 patients with histologically confirmed invasive ductal TNBC, who underwent surgery at Humanitas Clinical and Research Institute (Rozzano-Milan, Italy) from 2006 to 2011. To validate our findings, an additional independent cohort of TNBC samples (*N*=95) who underwent surgery at Humanitas Oncology Center of Catania (Catania, Italy; *N*=59) and Semmelweis University Hospital (Budapest, Hungary; *N*=36) from 1999 to 2008 was used. Estrogen, progesterone, and HER2 status were centrally assessed by immunohistochemistry and/or fluorescence *in situ* hybridization in ~90% of patients at Humanitas Clinical and Research Institute. The study was approved by the ethical committees of the Italian and Hungarian Institutions. The REporting of tumor MARKer Studies (REMARK) guidelines were followed in reporting results of this study.^42^ All patients were treated with adjuvant anthracycline–taxane-based chemotherapy. Clinical characteristics of patients included in this study are presented in Supplementary Table 1.

### Immunohistochemistry, immunofluorescence, and evaluation of staining

FFPE sections (3 μm) from TNBC patients included in the discovery (*N*=203) and validation (*N*=95) cohorts were incubated with AXL (R&D Systems, Minneapolis, MN, USA), CD163 (Novocastra, Newcastle, UK), and CD68 (Dako, Glostrup, Denmark) antibodies for 1 h at room temperature. CD68 and CD163 staining in the tumor stroma was scored using a four-tiered system ranging from 0 (absent) to 3 (dense).^43^ AXL staining was scored semiquantitatively, as previously described.^21^ In brief, intensity was recorded as 0 (no staining), 1 (weak staining), 2 (moderate staining), or 3 (strong staining), and the proportion of positive tumor cells was defined as the following: 0<1%; 1=1–9%; 2=10–49%; and 3⩾50%. A composite staining index was calculated by multiplying the intensity by the percentage of positive cells, and patients were stratified by low (0–4) or high (6–9) AXL expression for statistical analyses. The optimal cut-off point was determined by maximizing the sum of sensitivity and specificity. AXL expression was also evaluated as a continuous variable. For immunofluorescence, sections were incubated with AXL and CD163 primary antibodies, and then with donkey anti-goat Alexa 488-conjugated (Life Technologies, Carlsbad, CA, USA) and donkey anti-mouse Alexa-647-conjugated (Life Technologies) antibodies. Slides were counterstained with 4,6-diamidino-2-phenylindole (DAPI). Images were captured using an Olympus BX53 or Olympus Fluoview FV1000 laser scanning confocal microscope (Olympus, Tokyo, Japan). Detailed procedures for immunohistochemistry and immunofluorescence are described in Supplementary Methods.

### Expression analysis by quantitative reverse-transcription PCR

On the basis of a comprehensive literature review, we selected the 30 most functionally relevant kinases associated with EMT in breast cancer (Supplementary Table 2), and evaluated their expression by qRT-PCR, as described in Supplementary Methods. We also assessed the expression of conventional EMT (*CDH1* and *VIM*), and basal (*EGFR*, *KRT5*, and *KRT6A*) markers, and of a panel of cytokines/chemokines in breast cancer cell lines. The expression of selected genes was evaluated using TaqMan probes from Applied Biosystems (Foster City, CA, USA), following the manufacturer’s guidelines (Supplementary Table 9).

### Gene expression normalization and molecular subtype definition

Publicly available gene expression data from 311 TNBC patients were collected (Supplementary Table 1) and used for correlative analysis. An additional cohort of 137 TNBC patients treated with adjuvant chemotherapy was analyzed to validate our findings from the discovery phase (Supplementary Table 1). Detailed *in silico* analyses are reported in Supplementary Methods.

### Cell cultures, treatments, and preparation of tumor-conditioned media

Breast cancer cell lines (BT-474, BT-483, BT-549, HCC38, HCC70, HCC1143, Hs578T, MCF-7, MDA-MB-157, MDA-MB-453, MDA-MB-231, MDA-MB-361, MDA-MB-436, MDA-MB-468, and T47D) were obtained from the American Type Culture Collection (Manassas, VA, USA), and grown according to the standard protocols at 37 °C with 5% CO_2_. Paclitaxel, doxorubicin, and R428 (Selleck Chemicals, Houston, TX, USA) were dissolved in dimethyl sulfoxide. Cells were treated with paclitaxel (25 nmol/l), doxorubicin (1 μmol/l), R428 (1 μmol/l), or control vehicle. Once grown to sub-confluence, cells were serum starved and incubated with fresh medium for 24 h. CM were collected and filtered at 0.2 μm.

### Macrophages differentiation

Human monocytes were obtained from normal donor buffy coat by two-step gradient centrifugation and then polarized. Detailed procedures are described in Supplementary Methods. Freshly isolated human monocytes were also cultured in the absence or presence of 30% CM from HCC38, MCF-7, MDA-MB-231, MDA-MB-436, or MDA-MB-468 for 6 days, as previously described.^12,44^

### Flow cytometry

Macrophages were treated as indicated in the text, and analyzed by flow cytometry on a FACS Canto flow cytometer (BD Biosciences, San Jose, CA, USA). Human FcRs were blocked using 1% human serum in phosphate-buffered saline. Cells were washed, resuspended in FACS buffer (0.5% bovine serum albumin and 0.05% NaN_3_ in phosphate-buffered saline), and stained with fluorochrome-conjugated monoclonal antibodies against CD163 and CD206, or appropriate isotype controls (BD Biosciences).

### Enzyme-linked immunosorbent assay

The levels of CCL18, IL-10, IL-12, and growth arrest-specific 6 (Gas6) in macrophage supernatants were measured by commercially available ELISA kits according to manufacturer’s instructions (R&D Systems). All experiments were performed with three wells for each condition and repeated four times.

### Cell viability and wound-healing assays

Viable cells were identified using the 3-[4,5-dimethylthiazol-2-yl]-2,5-diphenyltetrazolium bromide assay (Sigma Aldrich, Milan, Italy), as previously described.^45^ For the wound-healing assay, breast cancer cells were seeded in six-well plates, and then scraped with a pipette tip. A detailed description of the assays can be found in Supplementary Methods.

### Western blotting

Immunodetection of proteins was performed using standard protocols. Further details on immunoblotting are provided in Supplementary Methods. The phospho-AXL (Tyr779) and AXL antibodies were purchased from R&D Systems. The phospho-AKT (Ser473), AKT, phospho-ERK1/2 (Thr202/Tyr204), ERK1/2, phospho-SRC (Tyr416), SRC, and β-actin were from Cell Signaling Technology (Danvers, MA, USA).

### Statistical analysis

Differences between two groups were determined using the Student’s *t-*test. Spearman’s rank and Pearson’s linear correlation tests were used to evaluate the correlation between variables. Clinicopathological associations were tested using Fisher’s exact test. Co-expression and enrichment analyses were performed using the Search-based Exploration of Expression Compendium (SEEK; http://seek.princeton.edu).^46^ Pathway analysis was performed using Ingenuity Pathway Analysis software (Qiagen, Redwood City, CA, USA). Survival analyses were performed by the Kaplan–Meier method, log-rank test (Mantel–Cox), and Cox univariate proportional hazard model. Multivariate Cox proportional hazard regression analysis was adjusted for relevant clinical covariates, including age at diagnosis, histological grade, lymph node status, tumor size, and tumor stage. *P* values were corrected using the Bonferroni or the Benjamini–Hochberg methods as indicated in the text. All tests were two-sided and the level of statistical significance was set at *P*<0.05. Statistical analyses were performed using GraphPad Prism version 5 (GraphPad Software, La Jolla, CA, USA), Epi Info version 7 (CDC, Atlanta, GA, USA), and R software (http://www.r-project.org). Further details on statistical methods are described in Supplementary Methods.

### Availability of data and materials

Information on the publicly available breast cancer data sets used in this study is provided in Supplementary Table 1.

## Figures and Tables

**Table 1:** Associations between CD68, CD163 and AXL proteins expression and clinicopathological features in triple-negative breast cancer

*Clinicopathological characteristics*	*CD68*				*CD163*				*AXL*			
	N	*High expression (%)*	*Low expression (%)*	P* value*^a^	N	*High expression (%)*	*Low expression (%)*	P* value*^a^	N	*High expression (%)*	*Low expression (%)*	P* value*^a^
*Age at diagnosis*												
<50	102	39 (38.2)	63 (61.8)	2.57E−01	102	44 (43.1)	58 (56.9)	8.88E−01	102	37 (36.3)	65 (63.7)	6.66E−01
⩾50	101	47 (46.5)	54 (53.5)		101	45 (44.6)	56 (55.4)		101	40 (39.6)	61 (60.4)	
												
*Lymph node status*												
Negative	96	35 (36.5)	61 (63.5)	1.19E−01	96	37 (38.5)	59 (58.3)	1.59E−01	96	29 (30.2)	67 (69.8)	**4.20E−02**
Positive	107	51 (47.7)	56 (52.3)		107	52 (48.6)	55 (51.4)		107	48 (44.9)	59(55.1)	
												
*Histological grade*												
G1–2	48	21 (43.7)	27 (56.3)	8.68E−01	48	26 (54.2)	22 (45.8)	1.34E−01	48	21 (43.7)	27 (56.3)	3.95E−01
G3	155	65 (41.9)	90 (58.1)		155	63 (40.6)	92 (59.4)		155	56 (36.1)	99 (63.9)	
												
*TNM staging*												
I–II	136	55 (40.4)	81 (59.6)	4.53E−01	136	58 (42.7)	78 (57.3)	6.54E−01	136	50 (36.8)	86 (63.2)	6.47E−01
III	67	31 (46.3)	36 (53.7)		67	31 (46.3)	36 (53.7)		67	27 (40.3)	40 (59.7)	
												
*Tumor size (mm)*												
⩽20	105	42 (40.0)	63 (60.0)	4.78E−01	105	47 (44.8)	58 (55.2)	8.88E−01	105	39 (37.1)	66 (62.9)	7.74E−01
>20	97	44 (45.4)	53 (54.6)		97	42 (43.3)	55 (56.7)		97	38 (39.2)	59 (60.8)	
												
*Lymphovascular invasion*												
Absent	65	29 (44.6)	36 (55.4)	4.06E−01	65	29 (44.6)	36 (55.4)	8.39E−01	65	24 (36.9)	41 (63.1)	8.35E−01
Present	38	13 (34.2)	25 (65.8)		38	16 (42.1)	22 (57.9)		38	15 (39.5)	23 (60.5)	
												
*Recurrence*												
Yes	47	26 (55.3)	21 (44.7)	**4.50E−02**	47	28 (59.6)	19 (40.4)	**1.80E−02**	47	31 (66.0)	16 (34.0)	**<1.00E−04**
No	156	60 (38.5)	96 (61.5)		156	61 (39.1)	95 (60.9)		156	46 (29.5)	110 (70.5)	
												
*First site of distant metastasis*												
Bone	8	2 (25.0)	6 (75.0)	1.01E−01	8	4 (50.0)	4 (50.0)	6.95E−01	8	3 (37.5)	5 (62.5)	**3.60E−02**
Visceral	25	16 (64.0)	9 (36.0)		25	15 (60.0)	10 (40.0)		25	20 (80.0)	5 (20.0)	

a Significant *P* values (Fisher’s exact test) are given in bold.

**Table 2:** Multivariate Cox regression analysis of AXL and CD163 for relapse-free survival and overall survival in triple-negative breast cancer (*N*=203)

*Variable*	*HR*	*95% CI*	P* value*^a^	*HR*	*95% CI*	P* value*^a^
	*Relapse-free survival*			*Overall survival*		
AXL	2.84	1.45–5.55	**2.20E−03**	3.09	1.58–6.06	**1.00E−03**
Age	0.88	0.46–1.69	7.11E−01	1.08	0.57–2.07	8.09E−01
Grade	1.68	0.69–4.11	2.53E−01	2.06	0.84–5.09	1.16E−01
Nodal status	2.53	1.01–6.31	4.72E−02	2.62	1.05–6.55	**3.98E−02**
Tumor size	1.33	0.69–2.58	3.97E−01	1.17	0.61–2.25	6.30E−01
Tumor stage	1.72	0.81–3.66	1.60E−01	1.67	0.78–3.57	1.88E−01
CD163	1.85	0.96–3.55	6.58E−02	1.63	0.85–3.12	1.40E−01
Age	0.95	0.50–1.80	8.72E−01	1.16	0.61–2.20	6.45E−01
Grade	1.68	0.68–4.13	2.60E−01	1.90	0.77–4.73	1.65E−01
Nodal status	2.90	1.18–7.13	**2.02E−02**	3.08	1.25–7.59	**1.43E−02**
Tumor size	1.36	0.70–2.65	3.60E−01	1.19	0.62–2.30	5.99E−01
Tumor stage	1.54	0.74–3.22	2.48E−01	1.41	0.67–2.94	3.66E−01

Abbreviations: CI, confidence interval; HR, hazard ratio.

a Significant *P* values are given in bold.

Multivariate analysis adjusted for age (⩾50 vs. <50), histological grade (G3 vs. G1–2), nodal status (1 vs. 0), tumor size (>20 mm vs. ⩽20 mm), and tumor stage (III vs. I–II).
