# Supplementary material for: AXL-associated tumor inflammation as a poor prognostic signature in chemotherapy-treated triple-negative breast cancer patients
Source: NPJ Breast Cancer. 2016 Nov 2;2:16033–. doi: 10.1038/npjbcancer.2016.33 (PMC5515347; doi:10.1038/npjbcancer.2016.33)
Supplement: Supplementary Methods [file npjbcancer201633-s1.pdf]

SUPPLEMENTARY METHODS

Immunohistochemistry, immunofluorescence, and evaluation of staining

Formalin-fixed, paraffin-embedded tissues (FFPE) sections (3 μm) from TNBC patients included in the discovery (N = 203) and in the validation (N = 95) cohorts were deparaffinized and subjected to epitope retrieval. For immunohistochemistry (IHC), tissue sections were treated with Peroxidase Blocking Reagent (Dako, Glostrup, Denmark) and Background Sniper (Biocare Medical, Concord, CA, USA), and then incubated with AXL (R&D Systems, Minneapolis, MN, USA), CD163 (Novocastra, Newcastle, UK), and CD68 (Dako) antibodies for 1 hr at room temperature. MACH 4 Universal HRP Polymer and diaminobenzidine (DAB; Biocare Medical) were used for chromogenic immunodetection, followed by counterstaining with hematoxylin. Negative control slides without primary antibody were included. Macrophages were quantified by screening the entire carcinoma area at low magnification using a x5 lens. Necrotic areas were excluded from the analysis, and only macrophages into tumor stroma were taken into account. Immunohistochemical evaluation was independently performed by two pathologists blinded for patient characteristics and outcome. At least three visual fields from different areas of each tumor were used for the evaluation. CD68-positive and CD163-positive macrophages were counted in three high-power fields per slide, and the mean number was then calculated. Finally, CD68 and CD163 staining in the tumor stroma was scored using a four-tiered system: 0 (no staining); 1 (few CD68- or CD163-positive macrophages); 2 (moderate number of CD68- or CD163-positive macrophages); 3 (multiple CD68- or CD163-positive macrophages).[1] For statistical analyses, these categories were dichotomized into absent/moderate (0 - 2) or dense (3) macrophage infiltration. AXL staining was scored semiquantitatively, as previously described.[2] Briefly, intensity was recorded as 0 (no staining), 1 (weak staining), 2 (moderate staining) or 3 (strong staining), and the proportion of positive tumor cells was defined as 0 < 1%; 1 = 1 - 9%; 2 = 10 - 49%; 3 ≥ 50%. A composite staining index was calculated by multiplying the intensity by the percentage of positive cells, and patients were stratified by low (0 - 4) or high (6 - 9) AXL expression for statistical analyses. The

optimal cut-off point was determined by maximizing the sum of sensitivity and specificity. AXL expression was also evaluated as a continuous variable. For immunofluorescence, sections were washed twice with PBS containing 0.05% Tween-20 (washing buffer; Sigma-Aldrich) and then blocked in PBS containing 2% bovine serum albumin and 2% donkey serum for 30 min at room temperature. Sections were then incubated with AXL and CD163 primary antibodies for 1 hr at room temperature, washed with washing buffer and then incubated with donkey anti-goat Alexa 488-conjugated (Life Technologies, Carlsbad, CA) and donkey anti-mouse Alexa-647-conjugated (Life Technologies) antibodies. Slides were counterstained with DAPI, mounted with ProLong Gold (Invitrogen, Carlsbad, CA) and stored in the dark at 4 °C. Images were captured using an Olympus BX53 or Olympus Fluoview FV1000 laser scanning confocal microscope (Olympus, Tokyo, Japan).

Expression analysis by quantitative reverse transcription PCR

We conducted a review of the literature using PubMed, Web of Science, and Embase databases from 2000 to 2016 using the search terms “breast cancer”, “epithelial-to-mesenchymal transition”, and “kinases”. Additional studies were identified through the references listed in review publications. Based on this comprehensive literature review, we selected the 30 most functionally relevant and well characterized kinases associated with EMT in breast cancer (Supplementary Table 2), and evaluated their expression by quantitative real-time PCR (qRT-PCR). We also assessed the expression of EMT (*CDH1*, and *VIM*), and basal (*EGFR*, *KRT5*, and *KRT6A*) markers, and a panel of cytokines/chemokines in breast cancer cell lines (Supplementary Table 8). Briefly, total RNA was extracted from two 6 µm-thick sections of FFPE breast tumor samples (N = 203) using the High Pure FFPE RNA Micro Kit (Roche, Mannheim, Germany), according to manufacturer’s instructions. Total RNA from cells was extracted using Trizol (Qiagen, Redwood City, CA, USA). The concentration and quality of RNA were assessed with NanoDrop ND-1000 Spectrophotometer (NanoDrop Technologies, Wilmington, DE, USA). The expression of selected genes was evaluated

using TaqMan probes from Applied Biosystems, following the manufacturer's guidelines (Supplementary Table 9). Three replicates per sample were assayed for each gene. PCR amplification was carried out using a ViiA 7 Real-Time PCR system (Applied Biosystems, Foster City, CA, USA), and glyceraldehyde-3-phosphate dehydrogenase (*GAPDH*) served as the normalizing control for relative quantification using the comparative CT method.

Gene expression normalization and molecular subtype definition

Publicly available gene expression data from 311 TNBC patients were collected (Supplementary Table 1). An additional cohort of 137 TNBC patients treated with adjuvant chemotherapy was analyzed (Supplementary Table 1). Gene expression data were generated with Affymetrix U133A (Affymetrix, Santa Clara, CA, USA) gene chips and normalized in the R environment using the affy Bioconductor package (http://www.bioconductor.org). For genes targeted by multiple microarray probes, only the probe set with the highest JetSet score was selected. ER and HER2 status were determined for each patient using the probe sets 205225_at and 216836_s_at, respectively.

Macrophages differentiation

Human monocytes were obtained from normal donor buffy coat by two-step gradient centrifugation, as previously described.[3] Briefly, non-adherent cells were removed, and purified monocytes were cultivated for 6 days in RPMI 1640 with 5% FBS and 50 ng/ml of recombinant human M-CSF (Peprotech, Rocky Hill, NJ, USA). M1 macrophages were polarized by culturing overnight the M-CSF-treated cells with LPS (100 ng/ml; Peprotech) and IFN-γ (100 ng/ml; Peprotech). M2 macrophages were obtained using IL-4 (20 ng/ml; Peprotech).[3] Conditioned media (CM) were collected and filtered at 0.2 µm. Freshly isolated human monocytes were also cultured in the absence or presence of 30% CM from HCC38, MCF-7, MDA-MB-231, MDA-MB-436, or MDA-MB-468 for 6 days.[3, 4]

Cell viability and wound healing assays

Viable cells were identified using the 3-[4,5-dimethylthiazol-2-yl]-2,5-diphenyltetrazolium bromide assay (MTT; Sigma Aldrich, Milan, Italy), as previously described.[5] Briefly, 3 x 10^3 MDA-MB-231 cells were plated in 96-well plates in DMEM supplemented with 10% FBS. Cells were allowed to attach overnight, and then treated as indicated in the text. After 72 hr, the MTT reagent (5 mg/ml in PBS) was added to each well, followed by an incubation for 4 hr at 37 °C. The MTT crystals were solubilized in DMSO. The absorbance was read at 560 nm with an iMark plate reader (Bio-Rad, Hercules, CA, USA). All the treatments were performed in triplicate, and cell viability was expressed as a percentage of the control (mean ± SD). For the wound healing assay, breast cancer cells were seeded in 6-well plates and grown at 37 °C in DMEM with 10% FBS. At 90% confluence, a scratch was produced using a pipette tip. Cells were then incubated with control medium or M2 macrophages-CM for 24 hr in the absence or presence of R428. Cell migration was captured immediately after stimulation (0 hr time point) and following 24 hr to monitor the closure of the wounded area. Image analysis was conducted with ImageJ software.

Western Blotting

Cells were washed with ice-cold PBS and scraped into ice-cold RIPA lysis buffer (Thermo Scientific, Rockford, IL, USA) supplemented with protease and phosphatase inhibitor cocktails (Thermo Scientific). Lysates were cleared by centrifugation at 13,000 rpm for 10 min at 4 °C, supernatants were removed and assayed for protein concentration using the Pierce BCA Protein Assay Kit (Thermo Scientific). Sixty micrograms of total lysate was resolved on SDS-PAGE gels and transferred to PVDF membranes (Bio-Rad). Membranes were blocked for 1 hr in 5% non-fat dry milk in Tris-buffered saline (TBS)-Tween and then hybridized using the primary antibodies in 5% non-fat dry milk TBS-Tween. The phospho-AXL (Tyr779), and AXL antibodies were purchased from R&D Systems. The phospho-AKT (Ser473), AKT, phospho-ERK1/2

(Thr202/Tyr204), ERK1/2, phospho-SRC (Tyr416), SRC, and β-actin were from Cell Signaling Technology (Danvers, MA, USA). Rabbit horseradish peroxidase (HRP)-conjugated secondary antibodies (Cell Signaling Technology) were diluted in 5% non-fat dry milk in TBS-Tween. Protein-antibody complexes were detected by chemiluminescence with Clarity Western ECL Substrate (Bio-Rad). Chemiluminescence imaging was performed on a Bio-Rad ChemiDoc MP Imager.

Statistical analysis

Differences between two groups were determined using the Student's *t* test. Spearman's rank and Pearson's linear correlation tests were used to evaluate the correlation between variables. Clinicopathological associations were tested using Fisher's exact test. Co-expression and enrichment analyses were performed using the Search-based Exploration of Expression Compendium (SEEK) (http://seek.princeton.edu).[6] Pathway analysis was performed using Ingenuity Pathway Analysis software (Qiagen). Assuming a survival rate based on a pilot study, with a two-sided significance of 0.05, the analysis of tissue samples from 123 patients will have 80% power to detect a difference in survival associated with the expression of the marker. Survival analyses were performed by the Kaplan-Meier method, log-rank test (Mantel-Cox), and Cox univariate proportional hazard model. Multivariate Cox proportional hazard regression analysis was adjusted for relevant clinical covariates, including age at diagnosis, histological grade, lymph node status, tumor size, and tumor stage. Patients who developed recurrence within 36 months after primary surgery were considered positive for tumor relapse, whereas patients who remained free of recurrence for the same time frame were defined as non-relapsing tumors. To investigate differential association with first site of distant metastases, recurrences were grouped within visceral, or bone. Relapse-free survival (RFS) was defined as the time from surgery until the detection of recurrence. Overall survival (OS) was defined as the time interval in months from surgery until the date of death or the most recent follow-up. For the additional cohort of adjuvant-

treated TNBC patients (N = 137) only RFS data were available. To correct for inter-study batch effects we performed a Cox regression analysis including single datasets as a potential confounding variable. We found that there was no survival difference between the datasets used to set up the database (P = 0.635; hazard ratio (HR) = 1.00; 95% confidence interval (CI), 0.99 - 1.01). P-values were corrected using the Bonferroni or the Benjamini-Hochberg methods as indicated in the text. All tests were two-sided and the level of statistical significance was set at $P < 0.05$. Statistical analyses were performed using GraphPad Prism version 5, Epi Info version 7, and R software.

REFERENCES

1. Medrek C, Pontén F, Jirström K, Leandersson K. The presence of tumor associated macrophages in tumor stroma as a prognostic marker for breast cancer patients. *BMC Cancer* **12**, 306 (2012).
2. Gjerdrum C, Tiron C, Høiby T, Stefansson I, Haugen H, Sandal T *et al.* Axl is an essential epithelial-to-mesenchymal transition-induced regulator of breast cancer metastasis and patient survival. *Proc Natl Acad Sci U S A* **107**, 1124-1129 (2010).
3. Solinas G, Schiarea S, Liguori M, Fabbri M, Pesce S, Zammataro L *et al.* Tumor-conditioned macrophages secrete migration-stimulating factor: a new marker for M2-polarization, influencing tumor cell motility. *J Immunol* **185**, 642-652 (2010).
4. Su S, Liu Q, Chen J, Chen J, Chen F, He C *et al.* A positive feedback loop between mesenchymal-like cancer cells and macrophages is essential to breast cancer metastasis. *Cancer Cell* **25**, 605-620 (2014).
5. Győrffy B, Bottai G, Lehmann-Che J, Kéri G, Orfi L, Iwamoto T *et al.* TP53 mutation-correlated genes predict the risk of tumor relapse and identify MPS1 as a potential therapeutic kinase in TP53-mutated breast cancers. *Mol Oncol* **8**, 508-519 (2014).

6. Aure MR, Leivonen SK, Fleischer T, Zhu Q, Overgaard J, Alsner J *et al*. Individual and combined effects of DNA methylation and copy number alterations on miRNA expression in breast tumors. *Genome Biol* **14**, R126 (2013).
