# Supplementary material for: AXL-associated tumor inflammation as a poor prognostic signature in chemotherapy-treated triple-negative breast cancer patients
Source: NPJ Breast Cancer. 2016 Nov 2;2:16033–. doi: 10.1038/npjbcancer.2016.33 (PMC5515347; doi:10.1038/npjbcancer.2016.33)
Supplement: Supplementary Tables [file npjbcancer201633-s2.pdf]

Supplementary Table 1. Patient characteristics.

Clinical and pathological information	*Discovery cohorts*				*Validation cohorts*	
	Internal cohort	*Affymetrix data sets*			*Affymetrix data sets*	*Internal cohort*
		Hatziz	*IGR*	*MAQC-II*		
Patients (N)	203	198	29	84	137	95
Median age, years (IQR)	50 (46-68)	48.2 (25-75)	49 (33-67)	50 (29-75)	-	51 (46-66)
Median tumor size, mm (IQR)	19 (15-32)	-	60 (20-175)	-	-	20 (9.5-30)
T stage						
T1	51.2%	6.6%	3.5%	13.1%	-	45.3%
T2	36.4%	47.0%	31.0%	38.1%	-	41.0%
T3	8.0%	32.8%	65.5%	22.6%	-	8.4%
T4	3.9%	13.1%	0.0%	21.4%	-	5.3%
Unknown	0.5%	0.5%	0.0%	4.8%	-	0.0%
TNM Stage						
I-II	67.0%	52.5%	58.6%	45.2%	-	58.9%
III	33.0%	46.5%	41.4%	50.0%	-	41.1%
Unknown	0.0%	1.0%	0.0%	4.8%	-	0.0%
Node status						
Negative	46.3%	27.3%	34.5%	19.0%	32.8%	45.3%
Positive	53.7%	72.7%	65.5%	79.8%	32.8%	54.7%
Unknown	0.0%	0.0%	0.0%	1.2%	34.4%	0.0%
Histological grade						
grade 1	1.0%	0.5%	0.0%	0.0%	2.2%	2.1%
grade 2	22.7%	12.7%	13.8%	17.9%	8.0%	20.0%
grade 3	76.3%	78.2%	75.9%	72.6%	65.7%	77.9%
Unknown		8.6%	10.3%	9.5%	24.1%	
Lymphovascular invasion						
Absent	32.0%	-	-	-	-	44.2%
Present	18.7%	-	-	-	-	34.7%
Unknown	49.3%	-	-	-	-	21.1%
Recurrence within 36 months						
Yes	19.7%	-	-	-	29.9%	18.9%
No	80.3%	-	-	-	70.1%	81.1%
Site of first distant recurrence						
Bone	17.0%	-	-	-	-	19.0%
Visceral	53.2%	-	-	-	-	61.9%
Unknown	29.8%				-	19.1%
Death of Breast cancer						
Yes	19.7%	-	-	-	-	20.0%
No	78.3%	-	-	-	-	80.0%
Unknown	2.0%	-	-	-	-	0.0%

**Series accession no**	GSE25066	GSE22093	GSE20194	GSE3494, GSE4611, GSE5327, GSE1456, GSE19615, GSE21653, GSE31519, GSE37946, GSE45255

Abbreviations: IQR, Interquartile range.

Supplementary Table 2. Correlations between CD68 protein expression and gene expression of the 30 most relevant EMT-associated kinases in-triple negative breast cancer (N = 203).

	CD68 protein expression		
EMT-associated kinases	***Spearman correlation coefficient***	**P-*value*[a]**	**P-*value*[b]**
AKT1	0.173	**1.37E-02**	3.43E-01
AKT2	-0.086	2.24E-01	2.47E+00
AXL	0.405	**< 1.00E-04**	**7.10E-03**
DDR1	0.048	4.98E-01	3.85E+00
DDR2	0.131	6.25E-02	1.06E+00
EGFR	0.03	6.67E-01	3.85E+00
ERK1	-0.125	7.55E-02	1.13E+00
ERK2	0.171	**1.47E-02**	3.53E-01
FAK	0.177	**1.18E-02**	3.19E-01
FGFR1	0.183	**9.00E-03**	2.52E-01
FGFR2	0.162	**2.08E-02**	4.38E-01
FGFR3	0.13	6.36E-02	1.06E+00
FYN	0.154	**2.81E-02**	5.32E-01
GSK3B	-0.005	9.47E-01	3.85E+00
IGF1R	0.006	9.34E-01	3.85E+00
IKKA	0.156	**2.59E-02**	5.18E-01
IKKB	0.163	**1.99E-02**	4.38E-01
LYN	0.174	**1.32E-02**	3.43E-01
MET	0.052	4.63E-01	3.85E+00
PDGFRA	-0.091	1.97E-01	2.36E+00
PDGFRB	-0.026	7.15E-01	3.85E+00
PTK2B	-0.064	3.65E-01	3.65E+00
SRC	0.056	4.28E-01	3.85E+00
SYK	-0.097	1.67E-01	2.16E+00
TGFBR1	-0.03	6.71E-01	3.85E+00
TGFBR2	0.168	**1.69E-02**	3.89E-01
VEGFR1	0.043	5.43E-01	3.85E+00
VEGFR2	0.111	1.17E-01	1.63E+00
VEGFR3	0.207	**3.10E-03**	8.99E-02
YES1	0.154	**2.80E-02**	5.32E-01

Significant *P*-values are given in bold.
[a]Spearman Rank correlation *P*-values.
[b]*P*-values corrected for multiple-testing error by the Bonferroni method.

Supplementary Table 3. SEEK analysis for the enrichment of genes co-expressed with *AXL* in triple negative breast cancer.

Gene ontology biological process	**P-*value*[a]**	***q value*[b]**
Extracellular matrix organization	4.54E-18	3.88E-17
Cell migration	9.99E-14	6.36E-13
Collagen metabolic process	9.99E-14	2.51E-12
Extracellular matrix disassembly	2.53E-13	9.73E-12
Collagen catabolic process	6.27E-13	3.20E-11
Cell adhesion	2.27E-10	1.52E-09
Positive regulation of immune system process	2.97E-10	2.11E-09
Collagen fibril organization	2.97E-10	2.86E-09
Regulation of cell migration	5.17E-10	6.26E-09
Regulation of cell motility	5.37E-10	6.26E-09
Regulation of cellular component movement	1.14E-09	1.17E-08
Leukocyte migration	3.29E-09	5.39E-08
Activation of immune response	3.19E-08	3.19E-07
Wound healing	8.34E-08	5.19E-07
Positive regulation of locomotion	8.34E-08	5.19E-07
Hemostasis	8.64E-08	5.85E-07
Cell activation	8.64E-08	5.85E-07
Blood coagulation	1.92E-07	1.32E-06
Positive regulation of cell motility	1.92E-07	1.38E-06
Cell chemotaxis	2.98E-07	2.72E-06
Positive regulation of cell migration	2.98E-07	3.50E-06
Chemotaxis	6.75E-07	5.01E-06
Complement activation	6.75E-07	6.83E-06
Cardiovascular system development	8.89E-07	9.08E-06
Blood vessel development	1.47E-06	2.32E-05
Platelet activation	2.09E-06	3.23E-05
Protein activation cascade	2.09E-06	3.37E-05
Humoral immune response	2.09E-06	5.71E-05
Regulation of response to external stimulus	7.78E-06	1.12E-04
Positive regulation of cell proliferation	1.46E-05	1.34E-04
Regulation of protein processing	1.46E-05	2.06E-04
Cell morphogenesis involved in differentiation	2.79E-05	2.37E-04
Leukocyte chemotaxis	2.79E-05	3.04E-04
Positive regulation of fibroblast proliferation	2.79E-05	3.58E-04
Collagen biosynthetic process	2.79E-05	3.63E-04
Regulation of smooth muscle cell migration	2.79E-05	3.63E-04
Cell substrate adhesion	2.79E-05	5.12E-04
Cell morphogenesis	9.61E-05	6.17E-04
Immune effector process	9.61E-05	6.26E-04
Regulation of leukocyte migration	9.61E-05	6.65E-04
Inflammatory response	9.61E-05	6.77E-04
Platelet degranulation	9.61E-05	8.26E-04

Regulation of chemotaxis	9.61E-05	8.87E-04
Immunoglobulin mediated immune response	9.61E-05	1.04E-03
Immune response regulating signaling pathway	1.22E-04	1.05E-03
Skeletal system development	1.22E-04	1.05E-03
Response to ketone	1.22E-04	1.89E-03
Negative regulation of immune system process	1.22E-04	2.01E-03
Regulation of cell activation	1.39E-04	2.59E-03
Positive regulation of chemotaxis	1.39E-04	2.59E-03
Positive regulation of leukocyte activation	1.39E-04	2.70E-03
Blood vessel morphogenesis	1.59E-04	2.70E-03
Negative regulation of cell proliferation	3.29E-04	3.28E-03
Regulation of leukocyte activation	3.29E-04	3.58E-03
Regulation of leukocyte chemotaxis	3.29E-04	3.58E-03
Regulation of multicellular organismal development	5.03E-04	3.79E-03
Regulation of epithelial to mesenchymal transition	5.03E-04	4.02E-03
Positive regulation of lymphocyte activation	5.03E-04	4.14E-03
Regulation of lymphocyte activation	5.03E-04	4.22E-03
Positive regulation of developmental process	5.03E-04	4.22E-03
Cellular component disassembly	5.03E-04	4.50E-03
Transmembrane receptor protein serine threonine kinase signaling pathway	5.03E-04	4.54E-03
Mesenchyme development	5.03E-04	4.92E-03
Regulation of complement activation	5.03E-04	5.49E-03
Epithelial to mesenchymal transition	5.03E-04	5.74E-03
Positive regulation of cell differentiation	5.03E-04	5.74E-03
Regulation of anatomical structure morphogenesis	5.03E-04	5.74E-03
Stem cell development	5.03E-04	5.74E-03
Protein processing	5.03E-04	5.74E-03
Platelet derived growth factor receptor signaling pathway	5.03E-04	6.62E-03
Positive regulation of response to external stimulus	5.03E-04	6.98E-03
Leukocyte activation	5.39E-04	7.04E-03
Regulation of leukocyte proliferation	5.39E-04	7.36E-03
Angiogenesis	5.39E-04	7.77E-03
Antigen receptor mediated signaling pathway	5.39E-04	7.77E-03
Proteoglycan biosynthetic process	5.39E-04	7.77E-03
Regulation of stem cell differentiation	5.39E-04	7.77E-03
Negative regulation of signaling	1.13E-03	7.77E-03
Macrophage activation	1.13E-03	7.77E-03
Ovulation cycle	1.13E-03	7.77E-03
Wnt receptor signaling pathway	1.13E-03	7.77E-03
Immune response regulating cell surface receptor signaling pathway	1.13E-03	7.77E-03
Regulation of Wnt receptor signaling pathway	1.13E-03	7.80E-03
Stem cell differentiation	1.13E-03	8.52E-03
Immune response activating signal transduction	1.13E-03	9.10E-03
Chemokine mediated signaling pathway	1.13E-03	9.66E-03
Lymphocyte activation	1.13E-03	1.11E-02
Negative regulation of canonical Wnt receptor signaling pathway	1.13E-03	1.18E-02

Proteoglycan metabolic process	1.13E-03	1.23E-02
Axon guidance	1.13E-03	1.23E-02
Negative regulation of signal transduction	1.75E-03	1.25E-02
Regulation of cell differentiation	2.03E-03	1.51E-02
Regulation of defense response	2.03E-03	1.55E-02
Negative regulation of Wnt receptor signaling pathway	2.03E-03	1.59E-02
Regulation of lymphocyte proliferation	2.03E-03	1.76E-02
Renal system development	2.03E-03	1.76E-02
Blood circulation	2.03E-03	1.80E-02
Regulation of canonical Wnt receptor signaling pathway	2.03E-03	1.86E-02
Regulation of cell morphogenesis	2.03E-03	1.93E-02
Cellular response to vascular endothelial growth factor stimulus	2.03E-03	2.10E-02
Endothelial cell migration	2.03E-03	2.13E-02
Positive regulation of Wnt receptor signaling pathway	2.03E-03	2.22E-02
Regulation of granulocyte chemotaxis	2.03E-03	2.31E-02
Negative regulation of developmental process	2.03E-03	2.48E-02
Vascular endothelial growth factor receptor signaling pathway	2.03E-03	3.01E-02
Positive regulation of intracellular protein transport	2.03E-03	3.07E-02
Regulation of wound healing	2.03E-03	3.09E-02
Positive regulation of leukocyte chemotaxis	2.03E-03	3.10E-02
Regulation of inflammatory response	2.03E-03	3.28E-02
Transforming growth factor beta receptor signaling pathway	2.03E-03	3.30E-02
Cellular response to transforming growth factor beta stimulus	2.03E-03	3.30E-02
Organ morphogenesis	2.15E-03	3.30E-02
Adaptive immune response based on somatic recombination of immune receptors built from immunoglobulin superfamily domains	2.15E-03	3.30E-02
Positive regulation of cell development	2.15E-03	3.30E-02
Positive regulation of intracellular transport	2.15E-03	3.70E-02
Canonical Wnt receptor signaling pathway	2.15E-03	3.70E-02
Cell cell adhesion	2.38E-03	3.99E-02
Exocytosis	2.38E-03	4.03E-02
Immune response activating cell surface receptor signaling pathway	3.68E-03	4.20E-02
Negative regulation of leukocyte proliferation	3.68E-03	4.36E-02
Regulation of B cell proliferation	3.68E-03	4.36E-02
Rregulation of B cell activation	4.07E-03	4.41E-02
Cell matrix adhesion	4.07E-03	4.41E-02
Anatomical structure formation involved in morphogenesis	4.07E-03	4.41E-02
Positive regulation of protein transport	4.07E-03	4.48E-02
Granulocyte chemotaxis	4.07E-03	4.54E-02
Positive regulation of lymphocyte proliferation	4.07E-03	4.54E-02
Morphogenesis of an epithelium	4.07E-03	4.54E-02
Positive regulation of nucleocytoplasmic transport	4.07E-03	4.54E-02

[a]Benjamini-Hochberg-adjusted.
[b]A threshold q value ≤ 0.05 for the Benjamini-Hochberg false discovery rate was used.

Supplementary Table 4. Genes enclosed in the M1 or M2 macrophage modules.

Gene symbol	***Gene name***
	M1-related module
APOL3	apolipoprotein L, 3
CCL15	chemokine (C-C motif) ligand 14; chemokine (C-C motif) ligand 15
CCL19	chemokine (C-C motif) ligand 19
CCL20	chemokine (C-C motif) ligand 20
CCL4	chemokine (C-C motif) ligand 4
CCL5	chemokine (C-C motif) ligand 5
CCR7	chemokine (C-C motif) receptor 7
CD69	CD69 molecule
CD80	CD80 molecule
CD86	CD86 molecule
CFB	complement factor B
CXCL1	chemokine (C-X-C motif) ligand 1 (melanoma growth stimulating activity, alpha)
CXCL10	chemokine (C-X-C motif) ligand 10
CXCL11	chemokine (C-X-C motif) ligand 11
CXCL9	chemokine (C-X-C motif) ligand 9
FCGR2A	Fc fragment of IgG, low affinity IIa, receptor (CD32)
FCGR3B	Fc fragment of IgG, low affinity IIIb, receptor (CD16b)
GBP1	guanylate binding protein 1, interferon-inducible, 67kDa
GBP2	guanylate binding protein 2, interferon-inducible
GBP3	guanylate binding protein 3
GBP4	guanylate binding protein 4
GBP5	guanylate binding protein 5
HESX1	HESX homeobox 1
ICAM1	intercellular adhesion molecule 1
IL12B	interleukin 12B
IL15	interleukin 15
IL15RA	interleukin 15 receptor, alpha
IL18	interleukin 18
IL1B	interleukin 1, beta
IL23A	interleukin 23, alpha subunit p19
IL2RA	interleukin 2 receptor, alpha
IL32	interleukin 32
IL6	interleukin 6
IRF1	interferon regulatory factor 1
IRF7	interferon regulatory factor 7
PDE4B	phosphodiesterase 4B, cAMP-specific
PTX3	pentraxin 3, long
SOCS3	suppressor of cytokine signaling 3
TLR2	toll-like receptor 2
TLR4	toll-like receptor 4
TNF	tumor necrosis factor
TNFSF10	tumor necrosis factor (ligand) superfamily, member 10
	M2-related module

ADK	adenosine kinase
ADORA3	adenosine A3 receptor
ALOX15	arachidonate 15-lipoxygenase
ARG1	arginase 1
CCL13	chemokine (C-C motif) ligand 13
CCL17	chemokine (C-C motif) ligand 17
CCL18	chemokine (C-C motif) ligand 18
CCL2	chemokine (C-C motif) ligand 2
CCL23	chemokine (C-C motif) ligand 23
CCL24	chemokine (C-C motif) ligand 24
CD163	CD163 molecule
CD36	CD36 molecule
CD9	CD9 molecule
CERK	ceramide kinase
CHN2	chimerin 2
COL6A2	collagen, type VI, alpha 2
CXCR4	chemokine (C-X-C motif) receptor 4
EGR2	early growth response 2
FGL2	fibrinogen-like 2
FN1	fibronectin 1
GAS6	growth arrest-specific 6
GAS7	growth arrest-specific 7
HEXB	hexosaminidase B
HNMT	histamine N-methyltransferase
HRH1	histamine receptor H1
HS3ST1	heparan sulfate (glucosamine) 3-O-sulfotransferase 1
IGF1	insulin-like growth factor 1 (somatomedin C)
IL10	interleukin 10
IL13	interleukin 13
IL1RN	interleukin 1 receptor antagonist
IL4	interleukin 4
IL4R	interleukin 4 receptor
LIPA	lipase A, lysosomal acid, cholesterol esterase
LTA4H	leukotriene A4 hydrolase
MAF	v-maf avian musculoaponeurotic fibrosarcoma oncogene homolog
MERTK	MER proto-oncogene, tyrosine kinase
MRC1	mannose receptor, C type 1
MRC2	mannose receptor, C type 2
MSR1	macrophage scavenger receptor 1
SEPP1	selenoprotein P, plasma, 1
TGFB1	transforming growth factor, beta 1
TGFBR2	transforming growth factor, beta receptor II
TLR5	toll-like receptor 5
TPST2	tyrosylprotein sulfotransferase 2

Supplementary Table 5. Genes co-expressed with the M1- or M2-related macrophage modules in triple-negative breast cancer.

Gene symbol	*Co-expression score*	P-*value*	*Gene symbol*	*Co-expression score*	P-*value*
M1-related module			*M2-related module*		
EPSTI1	2.349	< 1.00E-04	*C3AR1*	1.340	< 1.00E-04
UBD	2.139	< 1.00E-04	*MS4A4A*	1.308	< 1.00E-04
CSF2RB	2.110	< 1.00E-04	*KCTD12*	1.268	< 1.00E-04
TRBV20-1	1.741	< 1.00E-04	*MAFB*	1.253	< 1.00E-04
TICAM2	1.315	< 1.00E-04	*SERPINF1*	1.243	< 1.00E-04
C12orf59	1.265	< 1.00E-04	*TRBV20-1*	0.915	< 1.00E-04
LOC100130633	1.129	< 1.00E-04	*TICAM2*	0.735	< 1.00E-04
GBP1P1	1.108	< 1.00E-04	*LOC100130633*	0.610	< 1.00E-04
LOC729839	0.725	< 1.00E-04	*A2M-AS1*	0.503	< 1.00E-04
A2M-AS1	0.598	< 1.00E-04	*LOC729839*	0.340	< 1.00E-04
LOC645139	0.525	< 1.00E-04	*GBP1P1*	0.267	< 1.00E-04
LINC00460	0.487	< 1.00E-04	*LPP-AS2*	0.247	< 1.00E-04
TRBV7-8	0.474	< 1.00E-04	*LOC643072*	0.233	< 1.00E-04
LINC00294	0.294	< 1.00E-04	*LOC100131825*	0.230	< 1.00E-04
ZNF731P	0.285	< 1.00E-04	*STEAP1B*	0.215	< 1.00E-04
RPL18AP3	0.263	< 1.00E-04	*LOC100130458*	0.203	< 1.00E-04
LOC100130458	0.252	< 1.00E-04	*MUC5AC*	0.200	< 1.00E-04
LOC100288675	0.241	< 1.00E-04	*LINC00294*	0.197	< 1.00E-04
LOC100129637	0.238	< 1.00E-04	*LOC644456*	0.175	< 1.00E-04
LINC00284	0.191	< 1.00E-04	*LOC440311*	0.120	< 1.00E-04
LINC00582	0.184	< 1.00E-04	*LOC100132707*	0.120	< 1.00E-04
FLJ36848	0.180	< 1.00E-04	*DGCR10*	0.107	< 1.00E-04
TRBV7-3	0.171	< 1.00E-04	*LOC100131754*	0.090	< 1.00E-04
TMPO-AS1	0.167	< 1.00E-04	*LOC645139*	0.065	< 1.00E-04
LOC100288525	0.115	< 1.00E-04	*LOC100288418*	0.050	< 1.00E-04
LOC644589	0.100	< 1.00E-04	*TRBV7-8*	0.040	< 1.00E-04
MUC5AC	0.094	< 1.00E-04	*LOC100130111*	0.033	< 1.00E-04
LOC645739	0.081	< 1.00E-04	*LINC00284*	0.033	< 1.00E-04
LOC145474	0.057	< 1.00E-04	*ITPK1-AS1*	0.033	< 1.00E-04
FLJ37786	0.045	< 1.00E-04	*LOC145474*	0.013	< 1.00E-04
LPP-AS2	0.041	< 1.00E-04	*FLJ16779*	0.010	< 1.00E-04
LOC100132707	0.032	< 1.00E-04	*CDRT15L2*	-0.010	< 1.00E-04
ERLEC1P1	0.026	< 1.00E-04	*HCG11*	-0.013	< 1.00E-04
LOC100131754	0.026	< 1.00E-04	*TRIM78P*	-0.020	< 1.00E-04
LOC644456	0.021	< 1.00E-04	*LOC401463*	-0.020	< 1.00E-04
BLOC1S5	0.015	< 1.00E-04	*LOC100131261*	-0.020	< 1.00E-04
LOC643072	0.012	< 1.00E-04	*LOC100288675*	-0.025	< 1.00E-04
LOC151121	0.004	< 1.00E-04	*ANKRD34C*	-0.035	< 1.00E-04
DGCR10	-0.006	< 1.00E-04	*LOC100288578*	-0.040	< 1.00E-04
PAN3-AS1	-0.013	< 1.00E-04	*LINC00293*	-0.047	< 1.00E-04
SH3D20	-0.037	< 1.00E-04	*LOC100289058*	-0.050	< 1.00E-04
C6orf147	-0.039	< 1.00E-04	*RPL18AP3*	-0.060	< 1.00E-04
LOC339535	-0.055	< 1.00E-04	*HMGB3P30*	-0.060	< 1.00E-04
LOC100287598	-0.057	< 1.00E-04	*LOC340017*	-0.067	< 1.00E-04

C10orf112	-0.064	< 1.00E-04	*PAN3-AS1*	-0.073	< 1.00E-04
ITPK1-AS1	-0.065	< 1.00E-04	*LOC285205*	-0.073	< 1.00E-04
ANKRD34C	-0.069	< 1.00E-04	*LOC201651*	-0.077	< 1.00E-04
CDRT15L2	-0.077	< 1.00E-04	*LOC284632*	-0.093	< 1.00E-04
KCNU1	-0.093	< 1.00E-04	*LOC144438*	-0.095	< 1.00E-04
ZNF891	-0.100	< 1.00E-04	*LOC286367*	-0.100	< 1.00E-04
LOC440311	-0.100	< 1.00E-04	*LOC148824*	-0.103	< 1.00E-04
LOC100216545	-0.106	< 1.00E-04	*MUC19*	-0.105	< 1.00E-04
LOC255167	-0.106	< 1.00E-04	*C10orf112*	-0.107	< 1.00E-04
LINC00323	-0.118	< 1.00E-04	*XGPY2*	-0.110	< 1.00E-04
OSTM1-AS1	-0.136	< 1.00E-04	*LOC284551*	-0.113	< 1.00E-04
LOC116437	-0.138	< 1.00E-04	*KRTAP10-12*	-0.120	< 1.00E-04
C13orf41	-0.143	< 1.00E-04	*LOC100287825*	-0.125	< 1.00E-04
ZRANB2-AS1	-0.145	< 1.00E-04	*C13orf41*	-0.125	< 1.00E-04
LOC100289058	-0.146	< 1.00E-04	*LOC100216545*	-0.130	< 1.00E-04
LOC201651	-0.146	< 1.00E-04	*LOC100288152*	-0.133	< 1.00E-04
LINC00493	-0.147	< 1.00E-04	*FAM181A-AS1*	-0.133	< 1.00E-04
ST7-OT4	-0.152	< 1.00E-04	*LOC100127886*	-0.135	< 1.00E-04
LOC148824	-0.154	< 1.00E-04	*DCLRE1A*	-0.593	< 1.00E-04
LOC100131261	-0.159	< 1.00E-04	*ACVR2B*	-0.598	< 1.00E-04
LOC100288418	-0.164	< 1.00E-04	*VAPB*	-0.600	< 1.00E-04
LOC150577	-0.165	< 1.00E-04	*TGS1*	-0.600	< 1.00E-04
KIAA0141	-0.813	< 1.00E-04	*SETD1A*	-0.600	< 1.00E-04
SCAMP1	-0.819	< 1.00E-04	*ESPL1*	-0.600	< 1.00E-04
CAMK2N2	-0.831	< 1.00E-04	*UBAP2L*	-0.608	< 1.00E-04
HAND1	-0.844	< 1.00E-04	*CCNB2*	-0.610	< 1.00E-04
SNX21	-0.846	< 1.00E-04	*TBRG4*	-0.615	< 1.00E-04
PEX11G	-0.849	< 1.00E-04	*PSRC1*	-0.618	< 1.00E-04
MAGI2	-0.851	< 1.00E-04	*KIF20B*	-0.618	< 1.00E-04
ZNF251	-0.852	< 1.00E-04	*TIMELESS*	-0.620	< 1.00E-04
ACVR2B	-0.859	< 1.00E-04	*CABLES1*	-0.620	< 1.00E-04
SYNGR1	-0.859	< 1.00E-04	*CCDC34*	-0.623	< 1.00E-04
GTF2IRD1	-0.861	< 1.00E-04	*GINS4*	-0.625	< 1.00E-04
IGSF8	-0.868	< 1.00E-04	*CDC25A*	-0.625	< 1.00E-04
C3orf15	-0.878	< 1.00E-04	*KIF14*	-0.630	< 1.00E-04
CCDC114	-0.891	< 1.00E-04	*C12orf52*	-0.630	< 1.00E-04
FAM222A	-0.893	< 1.00E-04	*EIF2B4*	-0.633	< 1.00E-04
MTL5	-0.893	< 1.00E-04	*TICRR*	-0.633	< 1.00E-04
MAFG-AS1	-0.895	< 1.00E-04	*FAXC*	-0.633	< 1.00E-04
LOC81691	-0.900	< 1.00E-04	*C16orf13*	-0.633	< 1.00E-04
CCDC149	-0.902	< 1.00E-04	*POLR1C*	-0.640	< 1.00E-04
FLJ22184	-0.902	< 1.00E-04	*KIF18A*	-0.643	< 1.00E-04
TRPT1	-0.906	< 1.00E-04	*CCNF*	-0.643	< 1.00E-04
PIGQ	-0.908	< 1.00E-04	*MAFG-AS1*	-0.647	< 1.00E-04
SLC25A2	-0.912	< 1.00E-04	*C1orf135*	-0.648	< 1.00E-04
PLA2G6	-0.917	< 1.00E-04	*C16orf59*	-0.648	< 1.00E-04
SHF	-0.924	< 1.00E-04	*CDC45*	-0.650	< 1.00E-04
PPP1R16A	-0.927	< 1.00E-04	*KIFC2*	-0.653	< 1.00E-04
ROGDI	-0.927	< 1.00E-04	*C11orf83*	-0.657	< 1.00E-04
MRPL43	-0.930	< 1.00E-04	*PPFIA4*	-0.658	< 1.00E-04
FAM195A	-0.933	< 1.00E-04	*FAM20B*	-0.658	< 1.00E-04

NPEPL1	-0.938	< 1.00E-04	*FBXO44*	-0.660	< 1.00E-04
CACNB3	-0.941	< 1.00E-04	*ZNF74*	-0.663	< 1.00E-04
ITGA10	-0.949	< 1.00E-04	*TTC26*	-0.663	< 1.00E-04
TRIM45	-0.951	< 1.00E-04	*ZNF251*	-0.663	< 1.00E-04
TRMT10A	-0.954	< 1.00E-04	*ZNF696*	-0.668	< 1.00E-04
KIFC2	-0.956	< 1.00E-04	*ZC2HC1C*	-0.668	< 1.00E-04
ZC2HC1C	-0.970	< 1.00E-04	*RPP21*	-0.670	< 1.00E-04
ACY1	-0.979	< 1.00E-04	*LRRC27*	-0.670	< 1.00E-04
WDR96	-0.981	< 1.00E-04	*WDR34*	-0.673	< 1.00E-04
TMEM67	-0.986	< 1.00E-04	*WHSC1*	-0.688	< 1.00E-04
NUDT12	-1.000	< 1.00E-04	*GTPBP3*	-0.693	< 1.00E-04
FP588	-1.012	< 1.00E-04	*ZNF3*	-0.705	< 1.00E-04
IFT88	-1.012	< 1.00E-04	*CDC25C*	-0.705	< 1.00E-04
USP30	-1.025	< 1.00E-04	*ARHGAP39*	-0.707	< 1.00E-04
TMEM120A	-1.025	< 1.00E-04	*URB2*	-0.713	< 1.00E-04
CACNA1D	-1.050	< 1.00E-04	*RAD54L*	-0.718	< 1.00E-04
LINC00467	-1.064	< 1.00E-04	*WHSC2*	-0.728	< 1.00E-04
DYNLRB2	-1.075	< 1.00E-04	*ATAT1*	-0.730	< 1.00E-04
NUDT16L1	-1.086	< 1.00E-04	*RNFT2*	-0.738	< 1.00E-04
MAGI1	-1.088	< 1.00E-04	*GTF2IRD1*	-0.745	< 1.00E-04
LRRC27	-1.145	< 1.00E-04	*LOC81691*	-0.755	< 1.00E-04
KLC4	-1.147	< 1.00E-04	*SPIRE2*	-0.757	< 1.00E-04
CTSF	-1.178	< 1.00E-04	*RECQL4*	-0.773	< 1.00E-04
GARNL3	-1.189	< 1.00E-04	*TMEM106C*	-0.803	< 1.00E-04
MTERFD3	-1.190	< 1.00E-04	*C1orf162*	1.223	1.00E-04
CABLES1	-1.230	< 1.00E-04	*F13A1*	1.213	1.00E-04
LCP2	2.237	1.00E-04	*FCGR2B*	1.163	1.00E-04
FAM26F	2.219	1.00E-04	*DNAJA3*	-0.598	1.00E-04
TAGAP	2.111	1.00E-04	*PECAM1*	1.273	3.00E-04
CCL8	1.998	1.00E-04	*RNASE6*	1.240	3.00E-04
ADAMDEC1	1.961	1.00E-04	*DPP4*	0.958	3.00E-04
FBXO44	-0.823	1.00E-04	*TTI1*	-0.588	3.00E-04
RSPH1	-0.845	1.00E-04	*C3orf15*	-0.597	3.00E-04
TNFSF13B	2.125	2.00E-04	*C9orf100*	-0.607	3.00E-04
VCAM1	1.985	2.00E-04	*APOO*	-0.643	3.00E-04
SPEF2	-0.890	2.00E-04	*CSF1R*	1.273	4.00E-04
MMEL1	-0.904	2.00E-04	*GGTA1P*	1.127	4.00E-04
NLRC5	2.081	3.00E-04	*PTGER4*	1.063	4.00E-04
TFEC	2.022	3.00E-04	*ALDH1A1*	1.023	4.00E-04
CTSC	1.998	3.00E-04	*ZNF731P*	0.090	4.00E-04
LAMP3	1.995	3.00E-04	*LOC728095*	0.027	4.00E-04
MIR155HG	1.962	3.00E-04	*LOC645739*	-0.020	4.00E-04
ZBED2	1.658	3.00E-04	*CHL1-AS2*	-0.033	4.00E-04
TMEM129	-0.821	3.00E-04	*LOC91450*	-0.057	4.00E-04
LRRC8E	-0.898	3.00E-04	*LOC90784*	-0.067	4.00E-04
SAMD9L	2.320	4.00E-04	*FLJ37786*	-0.070	4.00E-04
PIK3AP1	1.942	4.00E-04	*BLOC1S5*	-0.070	4.00E-04
C16orf54	1.838	4.00E-04	*STL*	-0.077	4.00E-04
CXCL13	1.699	4.00E-04	*LOC730236*	-0.083	4.00E-04
FDCSP	1.271	4.00E-04	*LOC729570*	-0.090	4.00E-04
LOC91450	0.313	4.00E-04	*HIATL2*	-0.105	4.00E-04

OIP5-AS1	0.122	4.00E-04	*LINC00460*	-0.107	4.00E-04
EPR1	0.046	4.00E-04	*TGIF2LY*	-0.115	4.00E-04
LOC728095	0.019	4.00E-04	*GMNC*	-0.130	4.00E-04
LOC729683	0.005	4.00E-04	*SLC1A3*	0.958	5.00E-04
LOC90784	-0.028	4.00E-04	*NBPF3*	-0.610	5.00E-04
C6orf114	-0.061	4.00E-04	*MPEG1*	1.217	6.00E-04
LOC728142	-0.070	4.00E-04	*GIMAP6*	1.203	6.00E-04
HIATL2	-0.081	4.00E-04	*CFHR2*	0.888	6.00E-04
SNHG4	-0.092	4.00E-04	*UNG*	-0.653	6.00E-04
HCG2P7	-0.133	4.00E-04	*CXCL12*	1.198	7.00E-04
TGIF2LY	-0.134	4.00E-04	*SFRP2*	1.030	7.00E-04
LOC554207	-0.145	4.00E-04	*MS4A6A*	1.305	8.00E-04
ZFP2	-0.883	4.00E-04	*CPVL*	1.085	8.00E-04
CARD16	2.109	5.00E-04	*EPB41L3*	1.053	8.00E-04
FAM172A	-0.856	6.00E-04	*PTPRO*	0.990	8.00E-04
MPEG1	2.075	7.00E-04	*DSCC1*	-0.638	8.00E-04
MNDA	2.066	7.00E-04	*LGMN*	1.053	9.00E-04
GZMB	1.954	7.00E-04	*C1QC*	1.150	1.00E-03
ICOS	1.908	7.00E-04	*C12orf59*	0.563	1.00E-03
APOBEC3A	1.591	8.00E-04	*ZNF239*	-0.620	1.00E-03
PPFIA3	-0.839	8.00E-04	*MITF*	0.975	1.10E-03
PARD6G	-0.846	8.00E-04	*CAMK2N2*	-0.583	1.10E-03
NLRC3	1.923	9.00E-04	*SERPING1*	1.180	1.20E-03
STAMBPL1	1.884	9.00E-04	*GNG2*	1.093	1.20E-03
EGFL6	1.558	9.00E-04	*LHFPL2*	1.068	1.20E-03
CLEC4C	0.889	9.00E-04	*CCNB1*	-0.595	1.20E-03
BIRC3	2.055	1.00E-03	*FAM69A*	0.998	1.30E-03
AIM2	1.932	1.00E-03	*USP13*	-0.590	1.30E-03
SEL1L3	1.669	1.00E-03	*CLEC2B*	1.105	1.40E-03
IGLL3P	1.373	1.00E-03	*VSIG4*	1.170	1.50E-03
CYTIP	1.973	1.10E-03	*CNRIP1*	1.143	1.50E-03
CMPK2	1.953	1.10E-03	*FAM43A*	0.773	1.50E-03
IDO1	1.907	1.10E-03	*WIPF1*	1.213	1.60E-03
PLAC8	1.701	1.10E-03	*TLR4*	1.183	1.60E-03
RAB36	-0.807	1.10E-03	*GPR155*	0.990	1.60E-03
REEP6	-0.815	1.10E-03	*BASP1*	0.915	1.60E-03
SAMHD1	1.887	1.20E-03	*MME*	0.785	1.60E-03
HCP5	1.892	1.30E-03	*MTL5*	-0.578	1.60E-03
STPG1	-0.819	1.30E-03	*LRRC14*	-0.578	1.60E-03
SAMSN1	2.014	1.40E-03	*DOCK11*	1.193	1.70E-03
APOBEC3G	1.982	1.40E-03	*MNDA*	1.173	1.70E-03
CCR5	1.980	1.40E-03	*ENTPD1*	1.110	1.70E-03
PTGER4	1.665	1.40E-03	*ABCA1*	1.098	1.70E-03
MCOLN2	1.546	1.40E-03	*RCCD1*	-0.583	1.70E-03
ERAP2	1.265	1.40E-03	*SRSF12*	-0.593	1.70E-03
RRN3P2	0.970	1.40E-03	*MZT2B*	-0.620	1.70E-03
GNLY	1.755	1.50E-03	*CD53*	1.220	1.80E-03
CRTAM	1.672	1.50E-03	*CH25H*	0.930	1.80E-03
PLA2G7	1.570	1.50E-03	*JPH1*	-0.583	1.80E-03
HTRA4	1.120	1.50E-03	*DGCR14*	-0.605	1.80E-03
TMEM163	0.682	1.50E-03	*MCM7*	-0.638	1.90E-03

GIMAP5	2.058	1.60E-03	*DAB2*	1.218	2.00E-03
PTPRO	1.515	1.60E-03	*IL10RA*	1.213	2.00E-03
TNFRSF17	1.345	1.60E-03	*ADRB2*	0.805	2.00E-03
C10orf57	-0.836	1.60E-03	*NRN1*	0.670	2.00E-03
SLC1A3	1.250	1.70E-03	*SYCP3*	-0.533	2.00E-03
CD53	2.087	1.80E-03	*FLAD1*	-0.595	2.00E-03
CD38	1.715	1.80E-03	*RAP1GAP*	-0.670	2.00E-03
KIAA0226L	1.416	1.80E-03	*DNA2*	-0.685	2.00E-03
AGR3	-0.841	1.80E-03	*MRPS26*	-0.743	2.00E-03
TAPT1	-0.870	1.80E-03	*DOCK8*	1.153	2.10E-03
CYBB	2.021	1.90E-03	*GPNMB*	1.020	2.10E-03
MMP16	-0.807	1.90E-03	*ENPP2*	0.990	2.10E-03
ERGIC1	-0.814	1.90E-03	*TMEM200A*	0.867	2.10E-03
ZNF213	-0.859	1.90E-03	*COL6A6*	0.700	2.10E-03
DOCK8	1.983	2.00E-03	*PIAS2*	-0.573	2.10E-03
GZMA	1.924	2.00E-03	*CASKIN1*	-0.587	2.10E-03
LINC00277	-0.883	2.00E-03	*QTRT1*	-0.588	2.10E-03
CELF5	-0.919	2.00E-03	*RRP15*	-0.590	2.10E-03
RAP1GAP	-0.957	2.00E-03	*RAVER1*	-0.600	2.10E-03
NEDD4L	-1.069	2.00E-03	*WDR52*	-0.605	2.10E-03
TIGIT	1.666	2.10E-03	*CENPM*	-0.618	2.10E-03
C7orf29	0.683	2.10E-03	*PAXIP1*	-0.623	2.10E-03
GSTM3	-0.763	2.10E-03	*SBNO1*	-0.660	2.10E-03
C1orf21	-0.810	2.10E-03	*OVOL2*	-0.713	2.10E-03
RNF180	-0.816	2.10E-03	*CTSK*	1.170	2.20E-03
OVOL2	-0.836	2.10E-03	*GIMAP7*	1.103	2.20E-03
ATP6V0E2	-0.885	2.10E-03	*LPHN1*	-0.593	2.20E-03
EFCAB4A	-0.891	2.10E-03	*PPP1R16A*	-0.593	2.20E-03
CASKIN1	-0.915	2.10E-03	*PANK1*	-0.617	2.20E-03
SERINC5	-0.997	2.10E-03	*RAD18*	-0.627	2.20E-03
RASSF4	1.855	2.20E-03	*FBXO46*	-0.628	2.20E-03
IGJ	1.485	2.20E-03	*PMS2P1*	-0.643	2.20E-03
FUT4	1.273	2.20E-03	*NRTN*	-0.643	2.20E-03
CCND2	1.069	2.20E-03	*ESRP1*	-0.643	2.20E-03
IDO2	0.925	2.20E-03	*PACSIN3*	-0.655	2.20E-03
FAM196A	-0.698	2.20E-03	*FILIP1L*	1.118	2.30E-03
GNG3	-0.773	2.20E-03	*C1QB*	1.095	2.30E-03
WDR52	-0.797	2.20E-03	*PLA2G7*	0.850	2.30E-03
LPPR2	-0.818	2.20E-03	*ADIPOQ*	0.655	2.30E-03
GABRD	-0.832	2.20E-03	*FAM196A*	-0.493	2.30E-03
ZSWIM5	-0.908	2.20E-03	*RTTN*	-0.587	2.30E-03
MORN4	-0.962	2.20E-03	*UNC45A*	-0.595	2.30E-03
FLJ37453	-1.010	2.20E-03	*KIF11*	-0.608	2.30E-03
RAP2B	1.273	2.30E-03	*ZNF713*	-0.613	2.30E-03
ERICH1	1.006	2.30E-03	*SEPT3*	-0.663	2.30E-03
C14orf39	-0.671	2.30E-03	*C1QA*	1.108	2.40E-03
CCDC110	-0.784	2.30E-03	*PDPN*	0.995	2.40E-03
MLH3	-0.794	2.30E-03	*PLTP*	0.983	2.40E-03
C17orf104	-0.794	2.30E-03	*TBX20*	-0.540	2.40E-03
PACSIN3	-0.799	2.30E-03	*ECI1*	-0.585	2.40E-03
ZNF425	-0.814	2.30E-03	*C20orf20*	-0.590	2.40E-03

RABL5	-0.816	2.30E-03	*ASPM*	-0.598	2.40E-03
NPDC1	-0.832	2.30E-03	*FANCB*	-0.600	2.40E-03
TSTD1	-0.873	2.30E-03	*WDR66*	-0.627	2.40E-03
ATP8B1	-0.919	2.30E-03	*PLCH1*	-0.643	2.40E-03
EPN2	-1.004	2.30E-03	*ADAP2*	1.123	2.50E-03
GUSBP1	-1.010	2.30E-03	*NRP1*	1.068	2.50E-03
C7orf41	-1.024	2.30E-03	*S1PR1*	1.028	2.50E-03
STAT1	2.045	2.40E-03	*HYAL3*	-0.583	2.50E-03
HLA-DRB6	1.612	2.40E-03	*MYO19*	-0.588	2.50E-03
GUSBP11	1.408	2.40E-03	*ZNF7*	-0.593	2.50E-03
APOL4	1.211	2.40E-03	*CDCA3*	-0.595	2.50E-03
TBX20	-0.629	2.40E-03	*NFYA*	-0.600	2.50E-03
KLHL10	-0.691	2.40E-03	*PPAT*	-0.618	2.50E-03
WDR66	-0.769	2.40E-03	*DTL*	-0.655	2.50E-03
MIPEP	-0.787	2.40E-03	*NVL*	-0.668	2.50E-03
CERS4	-0.792	2.40E-03	*ILF3*	-0.678	2.50E-03
DECR2	-0.799	2.40E-03	*ANKRD54*	-0.713	2.50E-03
TCTN1	-0.824	2.40E-03	*INTS7*	-0.723	2.50E-03
ACADSB	-0.826	2.40E-03	*C10orf2*	-0.728	2.50E-03
SREBF1	-0.832	2.40E-03	*RAD54B*	-0.753	2.50E-03
RND2	-0.844	2.40E-03	*POP1*	-0.755	2.50E-03
DOCK6	-0.855	2.40E-03	*C1orf172*	-0.830	2.50E-03
KCNS2	-0.859	2.40E-03	*RCC1*	-0.835	2.50E-03
ASIC1	-0.917	2.40E-03	*CFI*	1.010	2.60E-03
PRRT3	-0.936	2.40E-03	*CCND2*	0.680	2.60E-03
MARVELD2	-1.113	2.40E-03	*KCTD4*	-0.530	2.60E-03
TTTY10	-0.707	2.50E-03	*TDRD5*	-0.570	2.60E-03
FSIP1	-0.759	2.50E-03	*ZNF473*	-0.578	2.60E-03
DGKD	-0.796	2.50E-03	*MAGOHB*	-0.588	2.60E-03
ACVR1B	-0.797	2.50E-03	*CLUAP1*	-0.593	2.60E-03
RTN2	-0.813	2.50E-03	*CACNB3*	-0.608	2.60E-03
TTLL1	-0.818	2.50E-03	*SBK1*	-0.610	2.60E-03
KDM4B	-0.837	2.50E-03	*POLA1*	-0.628	2.60E-03
KCTD1	-0.840	2.50E-03	*NPM3*	-0.635	2.60E-03
CLUL1	-0.851	2.50E-03	*TMEM201*	-0.643	2.60E-03
WWC2-AS2	-0.854	2.50E-03	*CSNK1E*	-0.658	2.60E-03
LONRF2	-1.349	2.50E-03	*SOX12*	-0.670	2.60E-03
SRGN	2.075	2.60E-03	*MCM4*	-0.685	2.60E-03
GIMAP2	2.033	2.60E-03	*ARHGEF19*	-0.693	2.60E-03
KLRK1	1.813	2.60E-03	*WDR35*	-0.793	2.60E-03
IL21R	1.812	2.60E-03	*RNASE1*	1.003	2.70E-03
PYHIN1	1.773	2.60E-03	*FMO1*	0.963	2.70E-03
ANKRD22	1.693	2.60E-03	*LINC00341*	0.925	2.70E-03
AZGP1	-0.746	2.60E-03	*SHF*	-0.553	2.70E-03
STARD6	-0.748	2.60E-03	*TFAP2D*	-0.573	2.70E-03
TFAP2A	-0.817	2.60E-03	*BZRAP1*	-0.580	2.70E-03
RTN4RL1	-0.822	2.60E-03	*UBE3D*	-0.587	2.70E-03
SOX6	-0.834	2.60E-03	*SLC25A10*	-0.600	2.70E-03
BCAM	-0.842	2.60E-03	*LOC400752*	-0.617	2.70E-03
GSS	-0.859	2.60E-03	*CABYR*	-0.618	2.70E-03
CLUAP1	-0.865	2.60E-03	*TXLNG*	-0.620	2.70E-03

C1orf194	-0.869	2.60E-03	*MTBP*	-0.637	2.70E-03
ANKS1B	-0.899	2.60E-03	*FLVCR1*	-0.637	2.70E-03
PCP2	-0.965	2.60E-03	*LYSMD1*	-0.643	2.70E-03
SSH3	-0.995	2.60E-03	*SCRIB*	-0.645	2.70E-03
SPATA6	-1.073	2.60E-03	*NENF*	-0.660	2.70E-03
WDR35	-1.080	2.60E-03	*KRTCAP3*	-0.677	2.70E-03
LOC401052	-1.136	2.60E-03	*CCHCR1*	-0.690	2.70E-03
APOL1	1.924	2.70E-03	*BCL7A*	-0.733	2.70E-03
SLC7A7	1.912	2.70E-03	*SLC19A1*	-0.735	2.70E-03
HLA-DRB1	1.882	2.70E-03	*NR2C2AP*	-0.817	2.70E-03
PTPN22	1.863	2.70E-03	*STARD6*	-0.483	2.80E-03
LOC96610	1.180	2.70E-03	*C22orf29*	-0.545	2.80E-03
DNASE1	-0.708	2.70E-03	*SOX6*	-0.570	2.80E-03
IFNA21	-0.770	2.70E-03	*MCMDC2*	-0.570	2.80E-03
LOC400752	-0.829	2.70E-03	*SLMO1*	-0.578	2.80E-03
UCN	-0.840	2.70E-03	*HSPA4L*	-0.580	2.80E-03
TACC2	-0.844	2.70E-03	*GRHL2*	-0.583	2.80E-03
DIP2C	-0.887	2.70E-03	*KIFC1*	-0.590	2.80E-03
TMEM145	-0.888	2.70E-03	*SNRPD1*	-0.593	2.80E-03
SLC25A29	-0.896	2.70E-03	*SLC39A3*	-0.597	2.80E-03
RAB26	-0.904	2.70E-03	*CATSPER3*	-0.597	2.80E-03
TTC39A	-0.906	2.70E-03	*ANKRD23*	-0.597	2.80E-03
RGL2	-0.943	2.70E-03	*C10orf35*	-0.600	2.80E-03
DNAAF3	-0.996	2.70E-03	*GCAT*	-0.608	2.80E-03
LMF1	-1.017	2.70E-03	*PRDX2*	-0.610	2.80E-03
PPP1R13B	-1.039	2.70E-03	*SAC3D1*	-0.613	2.80E-03
LOC113230	-1.048	2.70E-03	*USP21*	-0.618	2.80E-03
MMAB	-1.140	2.70E-03	*SGOL1*	-0.627	2.80E-03
IL2RB	1.926	2.80E-03	*KIF22*	-0.635	2.80E-03
GCET2	1.471	2.80E-03	*KIAA1549*	-0.640	2.80E-03
OLR1	1.251	2.80E-03	*ANAPC7*	-0.640	2.80E-03
RAB42	0.716	2.80E-03	*FANCG*	-0.648	2.80E-03
METTL20	-0.743	2.80E-03	*FEM1A*	-0.650	2.80E-03
XRRA1	-0.768	2.80E-03	*NEK2*	-0.653	2.80E-03
PPFIA4	-0.771	2.80E-03	*C1orf112*	-0.653	2.80E-03
GRTP1	-0.776	2.80E-03	*C17orf76-AS1*	-0.653	2.80E-03
CPLX1	-0.791	2.80E-03	*POLE*	-0.658	2.80E-03
MGC3771	-0.796	2.80E-03	*E2F1*	-0.673	2.80E-03
ZNF527	-0.801	2.80E-03	*BOLA1*	-0.705	2.80E-03
PRRG2	-0.809	2.80E-03	*AGBL5*	-0.748	2.80E-03
NPNT	-0.812	2.80E-03	*ZEB2*	1.160	2.90E-03
GOLGA2P5	-0.814	2.80E-03	*SLFN11*	0.987	2.90E-03
GCAT	-0.826	2.80E-03	*CPXM1*	0.817	2.90E-03
DCXR	-0.827	2.80E-03	*CHST7*	0.680	2.90E-03
LOC338799	-0.832	2.80E-03	*C3orf80*	0.487	2.90E-03
FBRSL1	-0.832	2.80E-03	*RASD2*	-0.540	2.90E-03
ZNF687	-0.843	2.80E-03	*ATP6V1E2*	-0.543	2.90E-03
NECAB3	-0.843	2.80E-03	*FKBP4*	-0.593	2.90E-03
KIAA1549	-0.847	2.80E-03	*C1orf210*	-0.597	2.90E-03
PIK3R2	-0.852	2.80E-03	*TTLL12*	-0.610	2.90E-03
KIAA0556	-0.854	2.80E-03	*DPCD*	-0.613	2.90E-03

UNC5CL	-0.874	2.80E-03	*ATAD2*	-0.623	2.90E-03
GEMIN8	-0.887	2.80E-03	*WDR43*	-0.625	2.90E-03
ZNF395	-0.893	2.80E-03	*ZNF24*	-0.628	2.90E-03
PLEKHA6	-0.914	2.80E-03	*FLJ37453*	-0.657	2.90E-03
CADM2	-0.930	2.80E-03	*VAX2*	-0.665	2.90E-03
CATSPER3	-0.938	2.80E-03	*ZMYND19*	-0.687	2.90E-03
BBS1	-0.944	2.80E-03	*MCAT*	-0.688	2.90E-03
MCMDC2	-0.947	2.80E-03	*DOM3Z*	-0.775	2.90E-03
KIAA1429	-1.001	2.80E-03	*LAIR1*	1.183	3.00E-03
CHADL	-1.003	2.80E-03	*PCSK5*	0.788	3.00E-03
SPATA18	-1.017	2.80E-03	*LYG2*	-0.537	3.00E-03
FAAH	-1.083	2.80E-03	*CYB5R2*	-0.540	3.00E-03
PTPRC	2.053	2.90E-03	*SLC25A2*	-0.560	3.00E-03
MLKL	1.984	2.90E-03	*HES6*	-0.563	3.00E-03
ACSL5	1.605	2.90E-03	*ZNF16*	-0.568	3.00E-03
ASPHD2	1.100	2.90E-03	*C1orf111*	-0.573	3.00E-03
FAM70A	0.714	2.90E-03	*KLHL17*	-0.577	3.00E-03
HOXC10	-0.675	2.90E-03	*MORC2*	-0.578	3.00E-03
TEX33	-0.699	2.90E-03	*POLR2H*	-0.580	3.00E-03
WBP2NL	-0.707	2.90E-03	*MIF*	-0.580	3.00E-03
KAZN	-0.751	2.90E-03	*GCDH*	-0.585	3.00E-03
FAM131A	-0.782	2.90E-03	*MLF1IP*	-0.593	3.00E-03
LRP2BP	-0.787	2.90E-03	*PHKA1*	-0.595	3.00E-03
ADARB2	-0.792	2.90E-03	*DICER1-AS1*	-0.597	3.00E-03
USP40	-0.802	2.90E-03	*PARPBP*	-0.600	3.00E-03
CCNB3	-0.806	2.90E-03	*NAA25*	-0.603	3.00E-03
DMRTB1	-0.809	2.90E-03	*TIMM44*	-0.615	3.00E-03
SLC22A17	-0.810	2.90E-03	*LIN9*	-0.617	3.00E-03
ASB14	-0.820	2.90E-03	*CKAP2L*	-0.617	3.00E-03
DNASE1L2	-0.827	2.90E-03	*TOP1MT*	-0.623	3.00E-03
C2orf77	-0.836	2.90E-03	*AZI1*	-0.635	3.00E-03
IGF1R	-0.839	2.90E-03	*CIT*	-0.638	3.00E-03
C20orf96	-0.860	2.90E-03	*OR7E24*	-0.655	3.00E-03
ARHGAP33	-0.864	2.90E-03	*TMEM97*	-0.660	3.00E-03
GKAP1	-0.867	2.90E-03	*GINS2*	-0.675	3.00E-03
CMBL	-0.884	2.90E-03	*MGC27345*	-0.677	3.00E-03
LCA5L	-0.885	2.90E-03	*MYEF2*	-0.683	3.00E-03
SPRYD7	-0.890	2.90E-03	*TROAP*	-0.690	3.00E-03
FAM174B	-0.940	2.90E-03	*SNRPE*	-0.720	3.00E-03
LRRC73	-1.008	2.90E-03	*GPR34*	0.987	3.10E-03
TMEM99	-1.067	2.90E-03	*ACP5*	0.890	3.10E-03
VPS37D	-1.190	2.90E-03	*CLUL1*	-0.513	3.10E-03
LYZ	1.723	3.00E-03	*ZSCAN20*	-0.513	3.10E-03
PLCL2	1.540	3.00E-03	*SDR9C7*	-0.517	3.10E-03
PLCXD2	1.003	3.00E-03	*TFDP2*	-0.538	3.10E-03
ANKRD36BP2	0.869	3.00E-03	*FHDC1*	-0.553	3.10E-03
ZNF713	-0.697	3.00E-03	*SLC5A8*	-0.557	3.10E-03
FAM228A	-0.698	3.00E-03	*FAM131A*	-0.558	3.10E-03
LYG2	-0.754	3.00E-03	*XRCC6BP1*	-0.567	3.10E-03
C6orf225	-0.794	3.00E-03	*RPRML*	-0.570	3.10E-03
PHKA1	-0.798	3.00E-03	*ABCA3*	-0.575	3.10E-03

LPHN1	-0.807	3.00E-03	*SUDS3*	-0.577	3.10E-03
THSD4	-0.809	3.00E-03	*F12*	-0.583	3.10E-03
MAST2	-0.816	3.00E-03	*LIN54*	-0.587	3.10E-03
NAT6	-0.818	3.00E-03	*DHTKD1*	-0.590	3.10E-03
SPPL3	-0.826	3.00E-03	*C12orf73*	-0.590	3.10E-03
RDH13	-0.827	3.00E-03	*HELLS*	-0.597	3.10E-03
SMCR7	-0.828	3.00E-03	*TCEB2*	-0.598	3.10E-03
SLC22A5	-0.831	3.00E-03	*ELAVL1*	-0.603	3.10E-03
UBN2	-0.839	3.00E-03	*STRA13*	-0.605	3.10E-03
FAM46B	-0.839	3.00E-03	*PHLPP1*	-0.610	3.10E-03
YBX2	-0.852	3.00E-03	*PRKCZ*	-0.618	3.10E-03
SHISA4	-0.887	3.00E-03	*GTF2H4*	-0.620	3.10E-03
MAGI3	-0.921	3.00E-03	*DNAH14*	-0.630	3.10E-03
SNRPE	-0.930	3.00E-03	*ZNF497*	-0.637	3.10E-03
JMJD8	-0.954	3.00E-03	*KIF15*	-0.640	3.10E-03
DICER1-AS1	-0.976	3.00E-03	*INTS3*	-0.650	3.10E-03
LRRC56	-0.987	3.00E-03	*TRAP1*	-0.670	3.10E-03
GOLT1A	-0.992	3.00E-03	*HMGXB4*	-0.670	3.10E-03
PRPF40B	-0.992	3.00E-03	*EXOSC2*	-0.683	3.10E-03
MYEF2	-1.098	3.00E-03	*PRR22*	-0.693	3.10E-03
RGS18	1.756	3.10E-03	*SFXN4*	-0.703	3.10E-03
NPL	1.730	3.10E-03	*CHDH*	-0.717	3.10E-03
LYSMD2	1.555	3.10E-03	*DHRS13*	-0.720	3.10E-03
MMP7	1.085	3.10E-03	*SLA*	1.178	3.20E-03
RAB9BP1	-0.659	3.10E-03	*SFRP4*	1.060	3.20E-03
HBZ	-0.690	3.10E-03	*ARL4C*	0.838	3.20E-03
CCDC148	-0.789	3.10E-03	*PCDHGC4*	-0.550	3.20E-03
C1orf226	-0.797	3.10E-03	*ACTR3B*	-0.563	3.20E-03
MZT2B	-0.798	3.10E-03	*ZNF326*	-0.580	3.20E-03
HKR1	-0.817	3.10E-03	*KHK*	-0.590	3.20E-03
ABCA3	-0.828	3.10E-03	*ZNF397*	-0.593	3.20E-03
BAIAP2	-0.832	3.10E-03	*ALKBH2*	-0.603	3.20E-03
PEX19	-0.844	3.10E-03	*FANCI*	-0.620	3.20E-03
PTOV1	-0.860	3.10E-03	*TSEN54*	-0.627	3.20E-03
SERINC2	-0.867	3.10E-03	*LIG1*	-0.678	3.20E-03
ZNF497	-0.869	3.10E-03	*SAYSD1*	-0.718	3.20E-03
TTC18	-0.874	3.10E-03	*CD320*	-0.720	3.20E-03
TBX3	-0.879	3.10E-03	*PSMC3IP*	-0.725	3.20E-03
LRRC43	-0.894	3.10E-03	*GIMAP4*	1.180	3.30E-03
BNIPL	-0.908	3.10E-03	*GALM*	0.867	3.30E-03
NR2E3	-0.909	3.10E-03	*FABP4*	0.700	3.30E-03
SERTAD4	-0.920	3.10E-03	*GPR37*	-0.460	3.30E-03
CASZ1	-0.923	3.10E-03	*DYDC2*	-0.513	3.30E-03
MAPT	-0.938	3.10E-03	*C2orf70*	-0.523	3.30E-03
RAB40C	-0.946	3.10E-03	*PAQR9*	-0.533	3.30E-03
DHRS13	-0.948	3.10E-03	*SERPINB11*	-0.540	3.30E-03
GAS8	-0.958	3.10E-03	*ANKS6*	-0.553	3.30E-03
WDR90	-1.074	3.10E-03	*FLJ25328*	-0.570	3.30E-03
CHDH	-1.110	3.10E-03	*ABCB8*	-0.585	3.30E-03
EFHC1	-1.148	3.10E-03	*PTCHD2*	-0.587	3.30E-03
LPAR5	1.203	3.20E-03	*TSFM*	-0.590	3.30E-03

COX7B2	0.347	3.20E-03	*POC1A*	-0.597	3.30E-03
HOXC4	-0.696	3.20E-03	*CDT1*	-0.600	3.30E-03
PBOV1	-0.800	3.20E-03	*STMN1*	-0.613	3.30E-03
SHANK2	-0.811	3.20E-03	*WEE1*	-0.615	3.30E-03
ZNF853	-0.813	3.20E-03	*PAK6*	-0.615	3.30E-03
CDKL3	-0.848	3.20E-03	*MORN2*	-0.627	3.30E-03
VPS13D	-0.865	3.20E-03	*CACYBP*	-0.628	3.30E-03
FBLL1	-0.875	3.20E-03	*SNHG11*	-0.630	3.30E-03
FBXL16	-0.927	3.20E-03	*HOOK1*	-0.650	3.30E-03
MIPOL1	-0.971	3.20E-03	*CENPV*	-0.653	3.30E-03
PSMB9	2.098	3.30E-03	*TIGD1*	-0.680	3.30E-03
SERPINB9	1.548	3.30E-03	*POLD2*	-0.765	3.30E-03
SPATA9	-0.633	3.30E-03	*NT5C3L*	-0.860	3.30E-03
FOXA1	-0.733	3.30E-03	*NCF2*	1.155	3.40E-03
WDPCP	-0.743	3.30E-03	*LCP2*	1.145	3.40E-03
MSI2	-0.752	3.30E-03	*GPR65*	1.123	3.40E-03
RPS6KA6	-0.753	3.30E-03	*CARD6*	1.083	3.40E-03
ANKRD35	-0.759	3.30E-03	*NPL*	0.968	3.40E-03
NPHP1	-0.767	3.30E-03	*MCTP1*	0.908	3.40E-03
SLC16A5	-0.785	3.30E-03	*HIST1H2BO*	-0.465	3.40E-03
RNF112	-0.794	3.30E-03	*SIAH3*	-0.487	3.40E-03
MST1P9	-0.795	3.30E-03	*ZNF511*	-0.580	3.40E-03
TMEM141	-0.800	3.30E-03	*ACBD6*	-0.580	3.40E-03
FAM210B	-0.802	3.30E-03	*CTPS2*	-0.588	3.40E-03
PLA2G12A	-0.805	3.30E-03	*STRN*	-0.590	3.40E-03
ABCB8	-0.824	3.30E-03	*KIF12*	-0.593	3.40E-03
DRD2	-0.832	3.30E-03	*PUF60*	-0.598	3.40E-03
COL28A1	-0.838	3.30E-03	*TMEM54*	-0.600	3.40E-03
RUNDC1	-0.840	3.30E-03	*LOC100128292*	-0.600	3.40E-03
FLJ25328	-0.854	3.30E-03	*NPEPL1*	-0.605	3.40E-03
TRIQK	-0.866	3.30E-03	*NR2F6*	-0.608	3.40E-03
TBC1D24	-0.870	3.30E-03	*UBAP2*	-0.610	3.40E-03
EPB41L5	-0.924	3.30E-03	*DYX1C1*	-0.620	3.40E-03
PLIN5	-0.951	3.30E-03	*C1orf51*	-0.633	3.40E-03
KIAA1244	-0.957	3.30E-03	*IMMP1L*	-0.637	3.40E-03
KIAA1467	-0.960	3.30E-03	*GNAZ*	-0.640	3.40E-03
SMIM1	-0.998	3.30E-03	*GCFC2*	-0.643	3.40E-03
CTDSPL	-1.032	3.30E-03	*STRBP*	-0.653	3.40E-03
NT5C3L	-1.046	3.30E-03	*TMEM18*	-0.667	3.40E-03
SNHG11	-1.095	3.30E-03	*IPO9*	-0.670	3.40E-03
TCEA3	-1.096	3.30E-03	*MDH1B*	-0.677	3.40E-03
ERBB3	-1.099	3.30E-03	*TTC28-AS1*	-0.720	3.40E-03
SUGT1P3	-1.130	3.30E-03	*PDGFRL*	0.848	3.50E-03
KLHL6	1.793	3.40E-03	*FAM24B*	-0.490	3.50E-03
LOC654433	0.419	3.40E-03	*C1orf226*	-0.520	3.50E-03
HOXC13	-0.707	3.40E-03	*ZP4*	-0.537	3.50E-03
FAM184B	-0.714	3.40E-03	*ZNF165*	-0.550	3.50E-03
PRSS35	-0.738	3.40E-03	*PCGF1*	-0.555	3.50E-03
LCE1B	-0.751	3.40E-03	*FANCE*	-0.563	3.50E-03
INPP5A	-0.785	3.40E-03	*COBL*	-0.563	3.50E-03
LOC728431	-0.786	3.40E-03	*FAM222A*	-0.567	3.50E-03

RAB17	-0.794	3.40E-03	*RTKN*	-0.583	3.50E-03
TMEM161B	-0.798	3.40E-03	*WDR90*	-0.587	3.50E-03
C6orf136	-0.799	3.40E-03	*RFC5*	-0.628	3.50E-03
PXMP4	-0.809	3.40E-03	*MZT2A*	-0.658	3.50E-03
HOXC11	-0.810	3.40E-03	*CENPF*	-0.673	3.50E-03
PRR22	-0.813	3.40E-03	*CEP85*	-0.678	3.50E-03
SPRED2	-0.851	3.40E-03	*ZNF250*	-0.683	3.50E-03
ARHGEF26	-0.872	3.40E-03	*FBXO41*	-0.685	3.50E-03
HSBP1L1	-0.885	3.40E-03	*NRF1*	-0.688	3.50E-03
KCTD6	-0.885	3.40E-03	*GIMAP8*	1.093	3.60E-03
ZNF709	-0.886	3.40E-03	*AKAP12*	0.940	3.60E-03
FLJ10661	-0.902	3.40E-03	*EML6*	-0.513	3.60E-03
FLJ90757	-0.905	3.40E-03	*CHRAC1*	-0.530	3.60E-03
MDH1B	-0.921	3.40E-03	*WDPCP*	-0.533	3.60E-03
TLE2	-0.942	3.40E-03	*WNK3*	-0.573	3.60E-03
HSD11B1L	-1.010	3.40E-03	*TIGD3*	-0.580	3.60E-03
KIF12	-1.073	3.40E-03	*VPS37D*	-0.583	3.60E-03
PARP14	2.040	3.50E-03	*NDUFAF4*	-0.590	3.60E-03
BTN2A2	1.569	3.50E-03	*PFDN6*	-0.593	3.60E-03
TMEM200A	1.277	3.50E-03	*TMEM143*	-0.607	3.60E-03
EMB	1.177	3.50E-03	*LSM4*	-0.608	3.60E-03
FAM46C	1.041	3.50E-03	*PSTK*	-0.623	3.60E-03
HOMER2	-0.669	3.50E-03	*C8orf82*	-0.627	3.60E-03
WDR17	-0.728	3.50E-03	*ZNF396*	-0.643	3.60E-03
AP4M1	-0.734	3.50E-03	*WDR60*	-0.648	3.60E-03
NOXRED1	-0.735	3.50E-03	*TRAIP*	-0.648	3.60E-03
C1orf100	-0.737	3.50E-03	*KIAA0895*	-0.653	3.60E-03
FGFR3	-0.787	3.50E-03	*GSTO2*	-0.690	3.60E-03
ACSS2	-0.791	3.50E-03	*MBLAC1*	-0.697	3.60E-03
ZNF3	-0.794	3.50E-03	*C16orf55*	-0.697	3.60E-03
SERTAD4-AS1	-0.825	3.50E-03	*CNKSR1*	-0.790	3.60E-03
ALDH6A1	-0.852	3.50E-03	*C1R*	1.185	3.70E-03
ZNF177	-0.858	3.50E-03	*C2*	1.073	3.70E-03
BTBD9	-0.864	3.50E-03	*HLA-DRB1*	1.038	3.70E-03
ZNF491	-0.868	3.50E-03	*VEGFC*	1.028	3.70E-03
SLC25A35	-0.870	3.50E-03	*CELF2*	0.968	3.70E-03
CCDC151	-0.899	3.50E-03	*C16orf54*	0.843	3.70E-03
C17orf28	-0.903	3.50E-03	*FAM70A*	0.505	3.70E-03
LOC100129794	-1.147	3.50E-03	*LRRC19*	-0.438	3.70E-03
CREB3L4	-1.290	3.50E-03	*RPS6KA6*	-0.520	3.70E-03
ST8SIA4	1.759	3.60E-03	*MFSD4*	-0.540	3.70E-03
SAMD3	1.642	3.60E-03	*COL28A1*	-0.540	3.70E-03
CXorf61	0.446	3.60E-03	*TDH*	-0.553	3.70E-03
FXYD4	-0.704	3.60E-03	*LOC440356*	-0.563	3.70E-03
RPH3AL	-0.707	3.60E-03	*MIPOL1*	-0.573	3.70E-03
OR7E24	-0.734	3.60E-03	*PGAP2*	-0.583	3.70E-03
NALCN	-0.735	3.60E-03	*SNX22*	-0.583	3.70E-03
CHST10	-0.790	3.60E-03	*LOC284889*	-0.583	3.70E-03
DUSP8	-0.800	3.60E-03	*AFG3L2*	-0.593	3.70E-03
SOGA1	-0.811	3.60E-03	*KIF2C*	-0.605	3.70E-03
TMEM9	-0.812	3.60E-03	*ARL9*	-0.617	3.70E-03

LRRC48	-0.817	3.60E-03	*PXMP2*	-0.635	3.70E-03
ADPRHL1	-0.838	3.60E-03	*DVL1*	-0.643	3.70E-03
ADCY9	-0.865	3.60E-03	*ZNF554*	-0.737	3.70E-03
POMT2	-0.870	3.60E-03	*CD93*	1.123	3.80E-03
LAMB2P1	-0.886	3.60E-03	*CECR1*	1.000	3.80E-03
ZCWPW2	-0.889	3.60E-03	*PLCL2*	0.858	3.80E-03
KRT18	-0.898	3.60E-03	*CDC14C*	-0.410	3.80E-03
GRK4	-0.962	3.60E-03	*C7orf13*	-0.467	3.80E-03
RHOD	-0.974	3.60E-03	*HIST1H2BB*	-0.475	3.80E-03
GSTO2	-1.046	3.60E-03	*TFAP2C*	-0.508	3.80E-03
WDR60	-1.144	3.60E-03	*TBL1X*	-0.510	3.80E-03
ZNF396	-1.278	3.60E-03	*BOLL*	-0.543	3.80E-03
GIMAP7	1.843	3.70E-03	*WDYHV1*	-0.560	3.80E-03
SLFN11	1.665	3.70E-03	*CECR5*	-0.565	3.80E-03
KLHDC7B	1.660	3.70E-03	*36892*	-0.577	3.80E-03
LAIR2	0.990	3.70E-03	*VPS72*	-0.580	3.80E-03
IDI2	-0.623	3.70E-03	*SCML2*	-0.595	3.80E-03
PLA2G10	-0.631	3.70E-03	*ZNF692*	-0.695	3.80E-03
LETM2	-0.680	3.70E-03	*C17orf69*	-0.723	3.80E-03
ETNK2	-0.681	3.70E-03	*FAM53A*	-0.727	3.80E-03
FHDC1	-0.732	3.70E-03	*FSTL1*	1.200	3.90E-03
MACROD2	-0.750	3.70E-03	*GREM1*	0.868	3.90E-03
PAK6	-0.755	3.70E-03	*ST3GAL5*	0.828	3.90E-03
MPP7	-0.759	3.70E-03	*CYP4Z2P*	0.253	3.90E-03
DYX1C1	-0.775	3.70E-03	*STK33*	-0.500	3.90E-03
CNNM2	-0.781	3.70E-03	*IBA57*	-0.537	3.90E-03
SH3RF1	-0.792	3.70E-03	*REPIN1*	-0.538	3.90E-03
WDR31	-0.809	3.70E-03	*TSNARE1*	-0.550	3.90E-03
SRCIN1	-0.818	3.70E-03	*CELSR2*	-0.573	3.90E-03
CRNDE	-0.843	3.70E-03	*GATAD2B*	-0.573	3.90E-03
LOC284440	-0.846	3.70E-03	*TMEM9*	-0.577	3.90E-03
DVL1	-0.852	3.70E-03	*FAM189B*	-0.623	3.90E-03
MKS1	-0.853	3.70E-03	*IL17RB*	-0.660	3.90E-03
VPS52	-0.870	3.70E-03	*SNHG15*	-0.687	3.90E-03
CCDC106	-0.879	3.70E-03	*GIMAP1*	1.083	4.00E-03
C1orf88	-0.956	3.70E-03	*C5orf62*	0.833	4.00E-03
LOC145837	-0.971	3.70E-03	*SLC22A3*	0.465	4.00E-03
ZNF554	-1.018	3.70E-03	*LDHC*	-0.495	4.00E-03
LILRB2	1.949	3.80E-03	*PPIL6*	-0.510	4.00E-03
SLAMF8	1.945	3.80E-03	*RNF8*	-0.530	4.00E-03
GCH1	1.847	3.80E-03	*HSBP1L1*	-0.553	4.00E-03
RNF145	1.256	3.80E-03	*IQGAP3*	-0.567	4.00E-03
SUCNR1	1.060	3.80E-03	*ASIC1*	-0.573	4.00E-03
PDE4DIP	0.864	3.80E-03	*DUS4L*	-0.615	4.00E-03
LRRC19	-0.587	3.80E-03	*ASNS*	-0.525	4.10E-03
PDZK1	-0.659	3.80E-03	*DSCR6*	-0.538	4.10E-03
RERG	-0.749	3.80E-03	*PLD6*	-0.543	4.10E-03
C17orf76-AS1	-0.769	3.80E-03	*C8orf49*	-0.573	4.10E-03
GNAZ	-0.783	3.80E-03	*POLE2*	-0.580	4.10E-03
FMO5	-0.785	3.80E-03	*TADA1*	-0.587	4.10E-03
STH	-0.810	3.80E-03	*CHAF1A*	-0.638	4.10E-03

MPP2	-0.818	3.80E-03	*EVI2B*	1.190	4.20E-03
KLHL26	-0.819	3.80E-03	*LY86*	1.150	4.20E-03
NOP14-AS1	-0.829	3.80E-03	*AP1S2*	0.998	4.20E-03
HHAT	-0.853	3.80E-03	*SERTAD4*	-0.503	4.20E-03
C17orf69	-0.917	3.80E-03	*GLB1L2*	-0.523	4.20E-03
C2orf63	-0.965	3.80E-03	*FKBPL*	-0.545	4.20E-03
ZNF692	-1.056	3.80E-03	*RBFA*	-0.573	4.20E-03
IRF8	1.873	3.90E-03	*OR52D1*	-0.590	4.20E-03
CLEC12A	1.444	3.90E-03	*SEPT5*	-0.600	4.20E-03
PCED1B	1.393	3.90E-03	*SSX2IP*	-0.640	4.20E-03
CTTN	-0.730	3.90E-03	*APITD1*	-0.645	4.20E-03
CYB5D1	-0.737	3.90E-03	*HLA-DMB*	1.118	4.30E-03
C20orf152	-0.743	3.90E-03	*PRKCH*	0.905	4.30E-03
CASC1	-0.744	3.90E-03	*MEOX2*	0.725	4.30E-03
ZNF596	-0.757	3.90E-03	*C22orf23*	-0.517	4.30E-03
C16orf45	-0.773	3.90E-03	*UPP2*	-0.547	4.30E-03
CSMD1	-0.780	3.90E-03	*SOGA1*	-0.547	4.30E-03
RING1	-0.814	3.90E-03	*NOP14-AS1*	-0.560	4.30E-03
LOC100132356	-0.924	3.90E-03	*CDK20*	-0.560	4.30E-03
KIAA1324	-0.933	3.90E-03	*SMIM1*	-0.573	4.30E-03
BBS5	-0.938	3.90E-03	*LOC100287482*	-0.640	4.30E-03
CC2D2A	-1.094	3.90E-03	*HLA-DMA*	1.110	4.40E-03
RSPO3	1.315	4.00E-03	*LAT2*	1.025	4.40E-03
NLRP7	0.682	4.00E-03	*STAMBPL1*	0.907	4.40E-03
RASEF	-0.715	4.00E-03	*NDN*	0.875	4.40E-03
AQP11	-0.729	4.00E-03	*LMO2*	0.835	4.40E-03
FAM71F1	-0.745	4.00E-03	*RAB36*	-0.530	4.40E-03
PC	-0.752	4.00E-03	*FLJ10661*	-0.530	4.40E-03
MYB	-0.771	4.00E-03	*TMEM241*	-0.557	4.40E-03
TMEM134	-0.807	4.00E-03	*WBSCR22*	-0.570	4.40E-03
SLC25A4	-0.819	4.00E-03	*AARS2*	-0.570	4.40E-03
C9orf152	-0.908	4.00E-03	*SEMA4F*	-0.578	4.40E-03
PLEKHH1	-0.912	4.00E-03	*USP39*	-0.653	4.40E-03
LOC284023	-0.913	4.00E-03	*TYROBP*	1.193	4.50E-03
ZHX3	-0.914	4.00E-03	*NCKAP1L*	1.178	4.50E-03
TMEM8B	-1.017	4.00E-03	*ITGAM*	1.125	4.50E-03
CLEC4A	1.922	4.10E-03	*HCLS1*	1.120	4.50E-03
GIMAP1	1.874	4.10E-03	*CPA3*	0.890	4.50E-03
CTLA4	1.760	4.10E-03	*STEAP1*	0.793	4.50E-03
RASSF5	1.755	4.10E-03	*SIX2*	-0.503	4.50E-03
ZNF319	1.076	4.10E-03	*SLC41A3*	-0.523	4.50E-03
NUDT4	-0.664	4.10E-03	*WDR73*	-0.548	4.50E-03
RAB15	-0.674	4.10E-03	*NDUFAF6*	-0.553	4.50E-03
KBTBD10	-0.752	4.10E-03	*ERBB3*	-0.573	4.50E-03
COQ7	-0.778	4.10E-03	*ZNF777*	-0.640	4.50E-03
CDAN1	-0.778	4.10E-03	*TMEM68*	-0.657	4.50E-03
SPIRE2	-0.782	4.10E-03	*KIF9*	-0.677	4.50E-03
CACNA2D2	-0.788	4.10E-03	*FAM83H*	-0.697	4.50E-03
ZNRF3	-0.788	4.10E-03	*LPAR5*	0.683	4.60E-03
MST1	-0.845	4.10E-03	*RAB9BP1*	-0.445	4.60E-03
WNK4	-0.867	4.10E-03	*MYO1H*	-0.453	4.60E-03

PLAC8L1	-0.882	4.10E-03	*ZSWIM3*	-0.470	4.60E-03
CASP1	2.007	4.20E-03	*MANEAL*	-0.507	4.60E-03
HLA-DQB1	1.740	4.20E-03	*CRCP*	-0.523	4.60E-03
CLEC2D	1.525	4.20E-03	*SNAP47*	-0.563	4.60E-03
SELE	0.996	4.20E-03	*HHEX*	0.730	4.70E-03
GCNT1	0.708	4.20E-03	*TFAP2A*	-0.508	4.70E-03
RSPO2	-0.606	4.20E-03	*SIM1*	-0.520	4.70E-03
GLS2	-0.737	4.20E-03	*SLC25A17*	-0.545	4.70E-03
ZNF563	-0.751	4.20E-03	*KIF3A*	-0.565	4.70E-03
C7orf63	-0.763	4.20E-03	*POLR2D*	-0.580	4.70E-03
RHOB	-0.765	4.20E-03	*LPXN*	1.058	4.80E-03
ZNF704	-0.776	4.20E-03	*CHL1*	0.638	4.80E-03
TMEM143	-0.800	4.20E-03	*GATC*	-0.470	4.80E-03
SEMA4F	-0.869	4.20E-03	*HFE2*	-0.530	4.80E-03
TJP3	-0.912	4.20E-03	*NT5C1A*	-0.540	4.80E-03
SEPT5	-0.968	4.20E-03	*LETM1*	-0.568	4.80E-03
ARHGAP25	1.919	4.30E-03	*CENPH*	-0.577	4.80E-03
APOC1	1.182	4.30E-03	*SURF2*	-0.580	4.80E-03
HOXC9	-0.631	4.30E-03	*CCR1*	1.138	4.90E-03
LCN12	-0.700	4.30E-03	*HTRA4*	0.597	4.90E-03
THNSL2	-0.707	4.30E-03	*PNPLA1*	-0.490	4.90E-03
BNIP3	-0.716	4.30E-03	*C17orf100*	-0.510	4.90E-03
ARHGAP32	-0.771	4.30E-03	*MTMR12*	-0.520	4.90E-03
LOC93622	-0.783	4.30E-03	*HPDL*	-0.550	4.90E-03
ATAT1	-0.797	4.30E-03	*FOXK2*	-0.558	4.90E-03
OSCP1	-0.871	4.30E-03	*NUDT16L1*	-0.567	4.90E-03
CPVL	1.560	4.40E-03	*DOCK10*	1.033	5.00E-03
TDO2	1.047	4.40E-03	*EPDR1*	0.703	5.00E-03
ARMC4	-0.652	4.40E-03	*PPARG*	0.610	5.00E-03
NBPF3	-0.732	4.40E-03	*UBE2U*	-0.403	5.00E-03
NMNAT3	-0.774	4.40E-03	*NEDD4L*	-0.518	5.00E-03
PPP1R36	-0.881	4.40E-03	*C3orf67*	-0.537	5.00E-03
FPR3	1.936	4.50E-03	*NMNAT3*	-0.550	5.00E-03
CD3D	1.892	4.50E-03	*ZNF687*	-0.557	5.00E-03
ZNF740	-0.688	4.50E-03	*RUVBL2*	-0.683	5.00E-03
MGC16275	-0.688	4.50E-03	*DOCK2*	1.105	5.10E-03
SIX2	-0.690	4.50E-03	*EVI2A*	1.090	5.10E-03
DACH2	-0.700	4.50E-03	*SGK1*	0.905	5.10E-03
PPP2R5A	-0.711	4.50E-03	*P2RY14*	0.803	5.10E-03
LMAN2L	-0.745	4.50E-03	*BTBD11*	0.363	5.10E-03
PIAS3	-0.752	4.50E-03	*HIST1H3F*	-0.485	5.10E-03
BMPR1B	-0.753	4.50E-03	*NUBP2*	-0.575	5.10E-03
ULK2	-0.767	4.50E-03	*SYCE3*	-0.583	5.10E-03
PHLPP1	-0.771	4.50E-03	*HLA-DPA1*	1.100	5.20E-03
DAK	-0.783	4.50E-03	*PTPRE*	1.053	5.20E-03
C20orf132	-0.788	4.50E-03	*RARRES2*	0.945	5.20E-03
FAM83H	-0.867	4.50E-03	*ADRA2A*	0.780	5.20E-03
ZBTB42	-0.893	4.50E-03	*FAM105A*	0.725	5.20E-03
TMEM68	-1.007	4.50E-03	*EGFL6*	0.648	5.20E-03
KIF9	-1.018	4.50E-03	*LOC148709*	-0.513	5.20E-03
GIMAP4	1.956	4.60E-03	*MLF1*	-0.530	5.20E-03

FLVCR2	1.302	4.60E-03	*TCTEX1D2*	-0.537	5.20E-03
LAG3	1.301	4.60E-03	*FAF1*	-0.545	5.20E-03
ADA	1.219	4.60E-03	*PAM16*	-0.745	5.20E-03
SLC39A8	0.933	4.60E-03	*IRF8*	1.013	5.30E-03
C5orf55	-0.706	4.60E-03	*APBB1IP*	0.993	5.30E-03
TBR1	-0.724	4.60E-03	*AKR1B1*	0.850	5.30E-03
NCKIPSD	-0.745	4.60E-03	*FIGLA*	-0.477	5.30E-03
ZG16B	-0.749	4.60E-03	*LOC728819*	-0.503	5.30E-03
CTBP2	-0.757	4.60E-03	*ST3GAL4*	-0.513	5.30E-03
UNC119B	-0.762	4.60E-03	*GJA10*	-0.533	5.30E-03
ABCC8	-0.771	4.60E-03	*ATP6V0E2*	-0.553	5.30E-03
ACAP3	-0.795	4.60E-03	*ADIG*	-0.567	5.30E-03
CCDC24	-0.820	4.60E-03	*SCARF1*	0.813	5.40E-03
CD48	1.926	4.70E-03	*PRR5L*	0.643	5.40E-03
GALM	1.325	4.70E-03	*C17orf104*	-0.447	5.40E-03
DHRS2	-0.609	4.70E-03	*POLB*	-0.498	5.40E-03
C22orf23	-0.693	4.70E-03	*DTNB*	-0.535	5.40E-03
ADAL	-0.713	4.70E-03	*TARBP1*	-0.555	5.40E-03
ZDHHC11	-0.731	4.70E-03	*CHERP*	-0.570	5.40E-03
FAM219B	-0.762	4.70E-03	*IL33*	0.773	5.50E-03
FBXL2	-0.769	4.70E-03	*VWDE*	-0.487	5.50E-03
ZNF768	-0.779	4.70E-03	*PLCB4*	-0.490	5.50E-03
C6orf170	-0.782	4.70E-03	*ZNF678*	-0.527	5.50E-03
ATP1A1OS	-0.794	4.70E-03	*NAP1L4*	-0.553	5.50E-03
EPB41L4A-AS1	-0.810	4.70E-03	*COL6A3*	1.045	5.60E-03
CTSS	2.062	4.80E-03	*SLC7A7*	1.043	5.60E-03
TRIM69	1.245	4.80E-03	*LUM*	1.035	5.60E-03
TOX2	0.843	4.80E-03	*TMEM213*	-0.417	5.60E-03
USP9Y	-0.554	4.80E-03	*ALMS1*	-0.578	5.60E-03
SCGB2A1	-0.610	4.80E-03	*SH2B3*	1.050	5.70E-03
ULBP3	-0.611	4.80E-03	*EMILIN2*	0.927	5.70E-03
HEATR4	-0.648	4.80E-03	*SRG7*	-0.433	5.70E-03
CGNL1	-0.708	4.80E-03	*ELL3*	-0.495	5.70E-03
RGNEF	-0.760	4.80E-03	*HIST1H2AB*	-0.500	5.70E-03
DTX3L	1.987	4.90E-03	*CCDC11*	-0.517	5.70E-03
HLA-DMB	1.913	4.90E-03	*NCAPG*	-0.580	5.70E-03
C1QC	1.811	4.90E-03	*MPP1*	0.833	5.80E-03
CCL2	1.759	4.90E-03	*TXNDC5*	0.770	5.80E-03
BATF2	1.661	4.90E-03	*LPL*	0.633	5.80E-03
LOC643733	1.330	4.90E-03	*CLEC4C*	0.387	5.80E-03
L3MBTL4	0.534	4.90E-03	*KATNAL2*	-0.480	5.80E-03
ZNF16	-0.696	4.90E-03	*CHRM3*	-0.483	5.80E-03
DOK7	-0.717	4.90E-03	*CHTOP*	-0.545	5.80E-03
FEM1A	-0.755	4.90E-03	*PLAC8L1*	-0.563	5.80E-03
CXXC11	-0.789	4.90E-03	*ITGB3BP*	-0.575	5.80E-03
LOC100271722	-0.987	4.90E-03	*BLM*	-0.580	5.80E-03
TAP1	2.020	5.00E-03	*IGJ*	0.755	5.90E-03
TNFAIP3	1.975	5.00E-03	*HOPX*	0.598	5.90E-03
PIM2	1.475	5.00E-03	*COX7B2*	0.190	5.90E-03
LOC439949	1.169	5.00E-03	*TCP10L*	-0.490	5.90E-03
GPRIN3	0.835	5.00E-03	*C1orf204*	-0.513	5.90E-03

UBE2U	-0.542	5.00E-03	*PDZD4*	-0.563	5.90E-03
GPC4	-0.601	5.00E-03	*DPYD*	1.120	6.00E-03
TAS2R5	-0.660	5.00E-03	*TSHZ3*	1.007	6.00E-03
C17orf58	-0.741	5.00E-03	*AOC3*	0.875	6.00E-03
EPB41L1	-0.754	5.00E-03	*SLC47A1*	0.408	6.00E-03
PDPK1	-0.760	5.00E-03	*CAMKMT*	-0.490	6.00E-03
PCGF2	-0.786	5.00E-03	*TMEM161A*	-0.575	6.00E-03
SYCE3	-0.876	5.00E-03	*MIS18A*	-0.575	6.00E-03
HLA-DRA	1.930	5.10E-03	*CRISPLD2*	1.005	6.10E-03
BTLA	1.457	5.10E-03	*CYSLTR1*	0.870	6.10E-03
POU2AF1	1.423	5.10E-03	*SMOC2*	0.683	6.10E-03
CLECL1	1.207	5.10E-03	*LOC96610*	0.650	6.10E-03
PALD1	0.851	5.10E-03	*HIST1H2AM*	-0.495	6.10E-03
IGLL1	0.572	5.10E-03	*PHF16*	-0.515	6.10E-03
RPL27A	-0.670	5.10E-03	*MST1P9*	-0.525	6.10E-03
PMS2P1	-0.688	5.10E-03	*TMEM52*	-0.537	6.10E-03
C3orf67	-0.738	5.10E-03	*LAPTM5*	1.155	6.20E-03
WDR78	-0.768	5.10E-03	*SLCO2B1*	1.080	6.20E-03
GNA11	-0.774	5.10E-03	*ELTD1*	0.993	6.20E-03
MARCH4	-0.779	5.10E-03	*CHRDL1*	0.765	6.20E-03
ATF7	-0.866	5.10E-03	*PTGS1*	0.743	6.20E-03
C5orf20	1.314	5.20E-03	*SLC35F3*	-0.417	6.20E-03
TXNDC5	1.291	5.20E-03	*ZDHHC23*	-0.507	6.20E-03
MMP12	1.136	5.20E-03	*GDEP*	-0.510	6.20E-03
LOC284801	-0.554	5.20E-03	*TIAL1*	-0.523	6.20E-03
C1orf51	-0.627	5.20E-03	*RHPN1-AS1*	-0.543	6.20E-03
FBXO36	-0.671	5.20E-03	*PPM1E*	-0.545	6.20E-03
EFR3B	-0.674	5.20E-03	*LILRB2*	1.088	6.30E-03
LGALS2	1.379	5.30E-03	*ANTXR2*	1.087	6.30E-03
PIWIL4	0.889	5.30E-03	*E2F5*	-0.520	6.30E-03
CHST2	0.819	5.30E-03	*LRFN4*	-0.550	6.30E-03
GTSF1	0.681	5.30E-03	*TMEM8B*	-0.570	6.30E-03
ANO5	-0.690	5.30E-03	*PRSS35*	-0.450	6.40E-03
NSUN7	-0.713	5.30E-03	*CNGA4*	-0.470	6.40E-03
ENPP1	-0.724	5.30E-03	*HILPDA*	-0.475	6.40E-03
REEP1	-0.771	5.30E-03	*ZNF643*	-0.515	6.40E-03
PBX1	-0.791	5.30E-03	*ZNF544*	-0.515	6.40E-03
C1orf162	1.855	5.40E-03	*LRRC73*	-0.533	6.40E-03
HPSE	1.295	5.40E-03	*TAF4*	-0.555	6.40E-03
CCDC71L	1.167	5.40E-03	*FAM181A*	-0.563	6.40E-03
NFE2L3	1.052	5.40E-03	*C7orf55*	-0.570	6.40E-03
PPP1R14C	0.808	5.40E-03	*NT5E*	0.888	6.50E-03
IL12A	0.435	5.40E-03	*LAYN*	0.887	6.50E-03
CST1	0.431	5.40E-03	*TMCC3*	0.660	6.50E-03
TNNT1	-0.660	5.40E-03	*IL31RA*	-0.490	6.50E-03
SPAG17	-0.661	5.40E-03	*CCSAP*	-0.493	6.50E-03
SHPK	-0.697	5.40E-03	*SOX4*	-0.513	6.50E-03
LYPD6	-0.707	5.40E-03	*RHEB*	-0.515	6.50E-03
OTUD7B	-0.734	5.40E-03	*LOC401588*	-0.517	6.50E-03
RAI1	-0.764	5.40E-03	*TM6SF1*	0.948	6.60E-03
RIBC1	-0.776	5.40E-03	*ITM2A*	0.830	6.60E-03

RAB11FIP4	-0.787	5.40E-03	*RAP2B*	0.610	6.60E-03
GPR65	1.882	5.50E-03	*HSD17B11*	1.078	6.70E-03
C1QB	1.837	5.50E-03	*CTSL1*	0.975	6.70E-03
EOMES	1.207	5.50E-03	*MILR1*	0.760	6.70E-03
C3orf80	0.476	5.50E-03	*RSPO3*	0.733	6.70E-03
DCD	-0.659	5.50E-03	*C18orf54*	-0.563	6.70E-03
DLX1	-0.674	5.50E-03	*SAMSN1*	1.020	6.80E-03
PLD6	-0.699	5.50E-03	*CFH*	0.973	6.80E-03
CAMK2N1	-0.706	5.50E-03	*TBXAS1*	0.958	6.80E-03
C1QTNF4	-0.752	5.50E-03	*SUPV3L1*	-0.540	6.80E-03
TMEM101	-0.771	5.50E-03	*AKAP1*	-0.563	6.80E-03
PYROXD2	-0.775	5.50E-03	*IGFBP6*	1.005	6.90E-03
KCTD3	-0.795	5.50E-03	*TFEC*	0.928	6.90E-03
IL18R1	1.314	5.60E-03	*GPX3*	0.805	6.90E-03
C15orf48	1.263	5.60E-03	*BBS5*	-0.437	6.90E-03
RASGEF1A	0.517	5.60E-03	*ARHGEF26*	-0.468	6.90E-03
MYO1H	-0.593	5.60E-03	*AS3MT*	-0.540	6.90E-03
LOC284889	-0.692	5.60E-03	*SRGN*	1.155	7.00E-03
C20orf26	-0.709	5.60E-03	*COL15A1*	0.923	7.00E-03
FAM200B	-0.766	5.60E-03	*LXN*	0.808	7.00E-03
EARS2	-0.775	5.60E-03	*WBP2NL*	-0.427	7.00E-03
LOC646762	-0.885	5.60E-03	*LINC00461*	-0.487	7.00E-03
SH3KBP1	1.377	5.70E-03	*INTS8*	-0.535	7.00E-03
NCKAP5	-0.621	5.70E-03	*CEP78*	-0.553	7.00E-03
C11orf88	-0.639	5.70E-03	*BRCA1*	-0.570	7.00E-03
COPG2	-0.669	5.70E-03	*KCNK5*	-0.445	7.10E-03
CRCP	-0.678	5.70E-03	*SUGT1P3*	-0.453	7.10E-03
PDE8B	-0.697	5.70E-03	*CABLES2*	-0.500	7.10E-03
LNX1	-0.736	5.70E-03	*COL3A1*	1.058	7.20E-03
PIAS4	-0.783	5.70E-03	*LOC441666*	-0.407	7.20E-03
C1QA	1.840	5.80E-03	*ZSWIM1*	-0.483	7.20E-03
CLEC7A	1.781	5.80E-03	*ZNF706*	-0.493	7.20E-03
TIFAB	1.654	5.80E-03	*ITGB2*	1.133	7.30E-03
TMEM51	1.100	5.80E-03	*DSE*	1.095	7.30E-03
TNIP3	0.912	5.80E-03	*FLI1*	1.055	7.30E-03
LIPG	0.829	5.80E-03	*SDPR*	0.755	7.30E-03
SYTL5	-0.551	5.80E-03	*CECR7*	-0.463	7.30E-03
SYBU	-0.680	5.80E-03	*ATP8B5P*	-0.473	7.30E-03
PPIP5K1	-0.696	5.80E-03	*LRRC1*	-0.498	7.30E-03
ZMYND12	-0.747	5.80E-03	*TMEM67*	-0.547	7.30E-03
C17orf108	-0.794	5.80E-03	*DIEXF*	-0.548	7.30E-03
ADCY6	-0.795	5.80E-03	*HRAS*	-0.575	7.30E-03
CD300LF	1.781	5.90E-03	*PDGFRA*	1.020	7.40E-03
THEMIS	1.553	5.90E-03	*KLHL6*	0.920	7.40E-03
CCL18	1.474	5.90E-03	*OLR1*	0.670	7.40E-03
CMTM8	-0.661	5.90E-03	*ID3*	0.613	7.40E-03
WBSCR27	-0.728	5.90E-03	*MEOX1*	0.543	7.40E-03
FAM53A	-0.740	5.90E-03	*C1orf56*	-0.468	7.40E-03
HERC5	1.601	6.00E-03	*C1S*	1.110	7.50E-03
P2RY14	1.253	6.00E-03	*HLA-DPB1*	0.983	7.50E-03
MMP1	1.094	6.00E-03	*VCAN*	0.950	7.50E-03

NUAK2	0.954	6.00E-03	*POSTN*	0.925	7.50E-03
ST6GALNAC5	0.946	6.00E-03	*ZNF423*	0.753	7.50E-03
GRIK4	-0.631	6.00E-03	*PCED1B*	0.723	7.50E-03
IRX5	-0.642	6.00E-03	*MMD*	0.690	7.50E-03
PRKAA2	-0.701	6.00E-03	*PDLIM1*	0.673	7.50E-03
IQCH	-0.710	6.00E-03	*HIST1H3B*	-0.493	7.50E-03
RIMS1	-0.730	6.00E-03	*FRAT2*	-0.525	7.50E-03
SUDS3	-0.761	6.00E-03	*ARHGAP15*	1.005	7.60E-03
EPN3	-0.761	6.00E-03	*FCGR3B*	0.773	7.60E-03
ST6GAL1	1.190	6.10E-03	*FLT1*	0.720	7.60E-03
COL22A1	0.884	6.10E-03	*PIP4K2A*	0.708	7.60E-03
DISP1	-0.690	6.10E-03	*PPAPDC1A*	0.663	7.60E-03
ENPP5	-0.715	6.10E-03	*TNFSF4*	0.655	7.60E-03
GPR98	-0.741	6.10E-03	*ANGPT2*	0.535	7.60E-03
XAF1	1.893	6.20E-03	*UMODL1*	-0.420	7.60E-03
LPXN	1.821	6.20E-03	*FAM81A*	-0.467	7.60E-03
RSAD2	1.763	6.20E-03	*QRICH2*	-0.480	7.60E-03
CAPZA3	-0.515	6.20E-03	*RAVER2*	-0.493	7.60E-03
ADAM32	-0.620	6.20E-03	*PPOX*	-0.558	7.60E-03
MGC2752	-0.695	6.20E-03	*CFD*	0.850	7.70E-03
FDXR	-0.724	6.20E-03	*NLRC3*	0.797	7.70E-03
BCDIN3D	-0.726	6.20E-03	*RGCC*	0.655	7.70E-03
SPDEF	-0.808	6.20E-03	*CASZ1*	-0.545	7.70E-03
FGL2	1.878	6.30E-03	*IFFO1*	1.028	7.80E-03
WARS	1.844	6.30E-03	*VGLL3*	0.953	7.80E-03
IL4I1	1.779	6.30E-03	*MFAP5*	0.870	7.80E-03
ALOX5	1.773	6.30E-03	*SOX7*	0.507	7.80E-03
TCF7	1.074	6.30E-03	*RNF32*	-0.438	7.80E-03
ATP1B4	-0.612	6.30E-03	*KAZN*	-0.488	7.80E-03
ABHD1	-0.725	6.30E-03	*TRPS1*	-0.495	7.80E-03
PROM2	-0.752	6.30E-03	*RIBC2*	-0.513	7.80E-03
TRAT1	1.610	6.40E-03	*ATG4D*	-0.557	7.80E-03
P2RX5	1.073	6.40E-03	*KRBA1*	-0.573	7.80E-03
KLRC3	0.571	6.40E-03	*DCN*	1.195	7.90E-03
C16orf55	-0.720	6.40E-03	*SUCNR1*	0.593	7.90E-03
ZNF20	-0.743	6.40E-03	*HIST3H3*	-0.448	7.90E-03
SEMA4D	1.543	6.50E-03	*MCF2L-AS1*	-0.505	7.90E-03
PCDHB13	-0.546	6.50E-03	*DLEU1*	-0.543	7.90E-03
ZNF77	-0.649	6.50E-03	*MKS1*	-0.553	7.90E-03
SLC25A27	-0.779	6.50E-03	*SIGLEC1*	1.063	8.00E-03
CSAD	-0.796	6.50E-03	*ARHGAP25*	1.048	8.00E-03
AS3MT	-0.959	6.50E-03	*TCF4*	0.978	8.00E-03
CD2	1.920	6.60E-03	*CMTM3*	0.970	8.00E-03
COL6A6	0.783	6.60E-03	*GAS1*	0.888	8.00E-03
CHRM3	-0.634	6.60E-03	*HLA-DRB6*	0.795	8.00E-03
RFESD	-0.712	6.60E-03	*CRTAM*	0.770	8.00E-03
WHSC2	-0.778	6.60E-03	*TRMT12*	-0.490	8.00E-03
TRIM22	2.018	6.70E-03	*PTPRC*	1.118	8.10E-03
HLA-DPA1	1.858	6.70E-03	*OLFML3*	1.033	8.10E-03
CD96	1.696	6.70E-03	*HAVCR2*	1.007	8.10E-03
NR1H3	1.271	6.70E-03	*SLC9A9*	0.940	8.10E-03

BCL11B	1.155	6.70E-03	*RHOXF1*	-0.463	8.10E-03
TRABD2A	0.834	6.70E-03	*CEP41*	-0.540	8.10E-03
HIST3H3	-0.618	6.70E-03	*CTSC*	0.948	8.20E-03
FLJ33630	-0.678	6.70E-03	*ANK2*	0.858	8.20E-03
PTGR2	-0.744	6.70E-03	*CCDC69*	0.790	8.20E-03
NRBP2	-0.764	6.70E-03	*RBP4*	0.468	8.20E-03
DHRS4-AS1	-0.782	6.70E-03	*STPG1*	-0.410	8.20E-03
ULK1	-0.801	6.70E-03	*POLH*	-0.470	8.20E-03
EMILIN2	1.581	6.80E-03	*FCER1G*	1.100	8.30E-03
SYK	1.465	6.80E-03	*G0S2*	0.730	8.30E-03
SLC2A5	1.043	6.80E-03	*APOC1*	0.653	8.30E-03
P2RY1	-0.591	6.80E-03	*TIGD4*	-0.493	8.30E-03
ZNF555	-0.656	6.80E-03	*SEC14L5*	-0.500	8.30E-03
EEF1A2	-0.699	6.80E-03	*MAP7*	-0.510	8.30E-03
GIMAP6	1.719	6.90E-03	*GTDC2*	-0.513	8.30E-03
RCSD1	1.645	6.90E-03	*ARL8A*	-0.517	8.30E-03
CLIC2	1.607	6.90E-03	*FYN*	0.915	8.40E-03
GPR155	1.460	6.90E-03	*PPAP2B*	0.883	8.40E-03
FKBP11	1.190	6.90E-03	*MAN1C1*	0.853	8.40E-03
SLITRK4	-0.539	6.90E-03	*FOXO1*	0.845	8.40E-03
CYP4X1	-0.633	6.90E-03	*DACT1*	0.715	8.40E-03
STK16	-0.672	6.90E-03	*PLAC8*	0.698	8.40E-03
BCL7A	-0.719	6.90E-03	*CXCR7*	0.633	8.40E-03
ZNF609	-0.779	6.90E-03	*MAEL*	0.257	8.40E-03
CD274	1.555	7.00E-03	*FSD1L*	-0.463	8.40E-03
ATP6V1B1	-0.633	7.00E-03	*BTBD6*	-0.483	8.40E-03
PPP1R3C	-0.737	7.00E-03	*SLC4A3*	-0.525	8.40E-03
HOXC8	-0.744	7.00E-03	*MMAB*	-0.530	8.40E-03
CD8A	1.762	7.10E-03	*SEPT7P2*	-0.540	8.40E-03
STAT4	1.705	7.10E-03	*ITGA4*	0.918	8.50E-03
SELL	1.680	7.10E-03	*GAL3ST4*	0.645	8.50E-03
DAPP1	1.582	7.10E-03	*XIST*	0.383	8.50E-03
ARHGAP39	-0.731	7.10E-03	*UTY*	-0.393	8.50E-03
BTN3A2	1.808	7.20E-03	*C6orf118*	-0.470	8.50E-03
GAL3ST4	0.977	7.20E-03	*DNMT3A*	-0.493	8.50E-03
CR1L	0.863	7.20E-03	*DAND5*	-0.517	8.50E-03
BHLHE41	0.826	7.20E-03	*NT5M*	-0.558	8.50E-03
SPINK13	-0.574	7.20E-03	*TLR7*	0.928	8.60E-03
HIST1H1T	-0.628	7.20E-03	*MMRN1*	0.598	8.60E-03
CCDC11	-0.688	7.20E-03	*TAS2R9*	-0.465	8.60E-03
SSR4P1	-0.689	7.20E-03	*RFESD*	-0.503	8.60E-03
BAG3	-0.744	7.20E-03	*MORN4*	-0.507	8.60E-03
POLE	-0.799	7.20E-03	*THNSL1*	-0.525	8.60E-03
EVI2B	1.928	7.30E-03	*SPON1*	0.893	8.70E-03
TNFAIP8	1.714	7.30E-03	*PDGFD*	0.780	8.70E-03
MB21D1	1.350	7.30E-03	*ERICH1*	0.503	8.70E-03
MAB21L3	-0.553	7.30E-03	*ULBP1*	-0.393	8.70E-03
GPR111	-0.603	7.30E-03	*DEPDC4*	-0.477	8.70E-03
CAB39L	-0.623	7.30E-03	*EPSTI1*	0.990	8.80E-03
LEFTY1	-0.630	7.30E-03	*ERG*	0.840	8.80E-03
MAP3K12	-0.761	7.30E-03	*CGREF1*	-0.473	8.80E-03

GPR174	0.937	7.40E-03	*C1orf87*	-0.490	8.80E-03
HORMAD1	0.398	7.40E-03	*BNIP3*	-0.493	8.80E-03
TMEM63C	-0.691	7.40E-03	*CDH24*	-0.523	8.80E-03
LOH12CR2	-0.723	7.40E-03	*GPR116*	0.863	8.90E-03
DSTYK	-0.734	7.40E-03	*CD1D*	0.670	8.90E-03
TXNRD2	-0.748	7.40E-03	*XG*	0.490	8.90E-03
PHKA2	-0.765	7.40E-03	*TBX15*	0.463	8.90E-03
SNX10	1.619	7.50E-03	*SAMHD1*	0.908	9.00E-03
SLC22A3	0.472	7.50E-03	*IL18*	0.653	9.00E-03
SLC44A5	-0.572	7.50E-03	*SPP1*	0.583	9.00E-03
KATNAL2	-0.616	7.50E-03	*FLVCR1-AS1*	-0.430	9.00E-03
ZACN	-0.627	7.50E-03	*NOL10*	-0.518	9.00E-03
PER2	-0.699	7.50E-03	*HOOK2*	-0.525	9.00E-03
IFI44L	1.822	7.60E-03	*TMEM61*	-0.530	9.00E-03
ITK	1.694	7.60E-03	*PAQR6*	-0.533	9.00E-03
CXorf21	1.297	7.60E-03	*ZNF133*	-0.555	9.00E-03
CXorf65	1.272	7.60E-03	*TMUB1*	-0.573	9.00E-03
CHST11	1.213	7.60E-03	*LPAR6*	0.945	9.10E-03
BBOX1	0.722	7.60E-03	*RASSF5*	0.873	9.10E-03
FAM227B	-0.507	7.60E-03	*CTSZ*	0.833	9.10E-03
FSIP2	-0.599	7.60E-03	*RSPO2*	-0.397	9.10E-03
SLAMF7	1.718	7.70E-03	*PHF13*	-0.490	9.10E-03
SCML4	1.363	7.70E-03	*DLX6*	-0.503	9.10E-03
EPHA7	-0.658	7.70E-03	*GPSM1*	-0.553	9.10E-03
PHF13	-0.691	7.70E-03	*CLIC2*	0.878	9.20E-03
AFF3	-0.703	7.70E-03	*TMEM37*	0.763	9.20E-03
INTU	-0.714	7.70E-03	*CNKSR2*	-0.458	9.20E-03
LATS1	-0.716	7.70E-03	*SNORA74A*	-0.537	9.20E-03
RRNAD1	-0.778	7.70E-03	*FAP*	1.003	9.30E-03
DOCK10	1.715	7.80E-03	*LYZ*	0.850	9.30E-03
BCL2A1	1.699	7.80E-03	*KIAA0226L*	0.635	9.30E-03
IFNG	1.314	7.80E-03	*BHLHE41*	0.563	9.30E-03
HIST1H2BA	-0.475	7.80E-03	*HOMER2*	-0.400	9.30E-03
ANXA9	-0.696	7.80E-03	*SIPA1L3*	-0.465	9.30E-03
FBXO15	-0.728	7.80E-03	*DLG3*	-0.498	9.30E-03
SLC19A2	-0.738	7.80E-03	*SETDB1*	-0.543	9.30E-03
ZFYVE21	-0.745	7.80E-03	*SEBOX*	-0.563	9.30E-03
BRF1	-0.803	7.80E-03	*CTSB*	1.150	9.40E-03
IFI16	1.933	7.90E-03	*CD86*	1.068	9.40E-03
FYB	1.910	7.90E-03	*PIK3AP1*	0.883	9.40E-03
LAT2	1.716	7.90E-03	*NEFH*	0.338	9.40E-03
PCDH20	-0.574	7.90E-03	*MGC16275*	-0.417	9.40E-03
TTC28-AS1	-0.648	7.90E-03	*DACH2*	-0.463	9.40E-03
MAPKBP1	-0.752	7.90E-03	*HIST1H1E*	-0.468	9.40E-03
HLA-DMA	1.852	8.00E-03	*MARCH1*	0.930	9.50E-03
IFI27	1.605	8.00E-03	*APOE*	0.813	9.50E-03
IGFLR1	1.563	8.00E-03	*LOC643733*	0.690	9.50E-03
EAF2	1.214	8.00E-03	*CST1*	0.273	9.50E-03
FAM105B	1.028	8.00E-03	*TMPRSS12*	-0.470	9.50E-03
SLC17A8	-0.600	8.00E-03	*C19orf52*	-0.517	9.50E-03
H19	-0.604	8.00E-03	*TNFRSF1B*	1.050	9.60E-03

SYCP3	-0.610	8.00E-03	*HLA-DRA*	1.003	9.60E-03
IQCF5	-0.692	8.00E-03	*P2RY13*	0.968	9.60E-03
BTBD17	-0.722	8.00E-03	*STAT4*	0.915	9.60E-03
DCTN2	-0.741	8.00E-03	*ERAP2*	0.573	9.60E-03
IL10RA	1.942	8.10E-03	*LAMP5*	0.465	9.60E-03
ARHGAP9	1.884	8.10E-03	*ZNF607*	-0.490	9.60E-03
PAQR9	-0.609	8.10E-03	*GPANK1*	-0.490	9.60E-03
CLVS1	-0.674	8.10E-03	*TSTD1*	-0.510	9.60E-03
PHYHD1	-0.693	8.10E-03	*NEIL3*	-0.555	9.60E-03
GSTCD	-0.734	8.10E-03	*EZH2*	-0.570	9.60E-03
ANKRD23	-0.747	8.10E-03	*LINC00476*	-0.447	9.70E-03
PLCD3	-0.755	8.10E-03	*C6orf136*	-0.540	9.70E-03
PAG1	1.312	8.20E-03	*NUDT14*	-0.543	9.70E-03
PLBD1	1.131	8.20E-03	*BTK*	0.980	9.80E-03
PSTPIP2	0.947	8.20E-03	*C1orf54*	0.873	9.80E-03
KCNN2	-0.566	8.20E-03	*SNX18*	0.467	9.80E-03
C4orf47	-0.645	8.20E-03	*TBC1D22B*	-0.463	9.80E-03
HCN2	-0.761	8.20E-03	*LOC100129794*	-0.480	9.80E-03
PAM16	-0.789	8.20E-03	*DPH2*	-0.565	9.80E-03
AOAH	1.725	8.30E-03	*C5AR1*	0.923	9.90E-03
NAPSB	1.659	8.30E-03	*HLA-DQB1*	0.868	9.90E-03
HCG26	1.449	8.30E-03	*MAP3K5*	0.833	9.90E-03
MICB	1.338	8.30E-03	*HTR2B*	0.585	9.90E-03
TEX14	-0.605	8.30E-03	*KCTD1*	-0.427	9.90E-03
FAM59A	-0.656	8.30E-03	*CCDC147*	-0.487	9.90E-03
C6orf226	-0.748	8.30E-03	*POLR3H*	-0.557	9.90E-03
PTCHD2	-0.784	8.30E-03	*IGSF6*	0.960	1.00E-02
HLA-F	1.985	8.40E-03	*GTSF1*	0.387	1.00E-02
IFIT3	1.910	8.40E-03	*LRRC8E*	-0.485	1.00E-02
NCF2	1.882	8.40E-03	*DHRS9*	0.753	1.01E-02
MERTK	0.768	8.40E-03	*CXorf59*	-0.500	1.01E-02
CXCL5	0.717	8.40E-03	*BDH1*	-0.523	1.01E-02
C1orf150	-0.499	8.40E-03	*CCDC58*	-0.533	1.01E-02
CCDC7	-0.562	8.40E-03	*LPAR1*	0.840	1.02E-02
FAM149A	-0.605	8.40E-03	*CA8*	-0.390	1.02E-02
METTL22	-0.652	8.40E-03	*GPRIN2*	-0.493	1.02E-02
GREB1	-0.657	8.40E-03	*TCEA3*	-0.503	1.02E-02
STK32C	-0.729	8.40E-03	*CLEC4A*	0.983	1.03E-02
PPM1E	-0.729	8.40E-03	*TEX26*	-0.433	1.03E-02
VIPR1	-0.752	8.40E-03	*SERTAD4-AS1*	-0.450	1.03E-02
ATXN2	-0.756	8.40E-03	*ESYT3*	-0.490	1.03E-02
ARHGEF37	-0.758	8.40E-03	*ZNF398*	-0.497	1.03E-02
C8orf82	-0.780	8.40E-03	*NID2*	0.863	1.04E-02
PLK5	-0.804	8.40E-03	*SLC16A7*	0.610	1.04E-02
UBE2L6	1.910	8.50E-03	*LOC283481*	-0.480	1.04E-02
PPP2R2D	-0.594	8.50E-03	*ZNRF3*	-0.500	1.04E-02
MFAP3L	-0.598	8.50E-03	*OTUB1*	-0.568	1.04E-02
ICA1L	-0.670	8.50E-03	*EXO1*	-0.575	1.04E-02
ADAMTS13	-0.799	8.50E-03	*SYNE1*	0.935	1.05E-02
LAPTM5	1.948	8.60E-03	*KIAA1462*	0.843	1.05E-02
CCR2	1.804	8.60E-03	*LMO4*	-0.395	1.05E-02

ITGA4	1.582	8.60E-03	*SPAG17*	-0.447	1.05E-02
COL4A4	0.899	8.60E-03	*CYP39A1*	-0.450	1.05E-02
AGPHD1	-0.636	8.60E-03	*HSF2BP*	-0.470	1.05E-02
IQCF1	-0.662	8.60E-03	*VARS2*	-0.533	1.05E-02
IQCK	-0.682	8.60E-03	*LDB2*	0.910	1.06E-02
SMAD9	-0.688	8.60E-03	*RGS18*	0.810	1.06E-02
PPP2R2C	-0.711	8.60E-03	*BCAT1*	0.653	1.06E-02
DOCK2	1.791	8.70E-03	*HOXA9*	0.225	1.06E-02
TAP2	1.652	8.70E-03	*ZBTB3*	-0.457	1.06E-02
GPR171	1.567	8.70E-03	*FAM71F1*	-0.510	1.06E-02
APOBEC3F	0.890	8.70E-03	*ZDHHC16*	-0.530	1.06E-02
KLRC4	0.505	8.70E-03	*ABI3BP*	0.850	1.07E-02
CDC14C	-0.482	8.70E-03	*PARP8*	0.805	1.07E-02
DNALI1	-0.643	8.70E-03	*SLC2A5*	0.615	1.07E-02
IGSF22	-0.670	8.70E-03	*KLHDC10*	-0.440	1.07E-02
NME5	-0.704	8.70E-03	*NOXRED1*	-0.447	1.07E-02
ZNF19	-0.715	8.70E-03	*FLJ30679*	-0.490	1.07E-02
RNASE6	1.738	8.80E-03	*ABCB9*	-0.533	1.07E-02
MX2	1.686	8.80E-03	*CXXC11*	-0.557	1.07E-02
FCGR2B	1.540	8.80E-03	*HEG1*	1.030	1.08E-02
STAP1	1.288	8.80E-03	*PDK4*	0.740	1.08E-02
TBL1X	-0.616	8.80E-03	*RALBP1*	-0.430	1.08E-02
CLGN	-0.623	8.80E-03	*UNC80*	-0.490	1.08E-02
ICA1	-0.631	8.80E-03	*LOC388796*	-0.513	1.08E-02
CCDC125	-0.655	8.80E-03	*BZW2*	-0.528	1.08E-02
LOC728485	-0.763	8.80E-03	*EPAS1*	0.990	1.09E-02
CELF2	1.451	8.90E-03	*ST8SIA4*	0.840	1.09E-02
RABGAP1L	1.446	8.90E-03	*ZSWIM2*	-0.430	1.09E-02
CD163L1	0.562	8.90E-03	*TMEM74*	-0.457	1.09E-02
LAMA1	0.472	8.90E-03	*SLC6A8*	-0.508	1.09E-02
C10orf113	0.254	8.90E-03	*DPT*	0.988	1.10E-02
UGT1A6	-0.599	8.90E-03	*PEAR1*	0.763	1.10E-02
SCGB1D2	-0.618	8.90E-03	*SEL1L3*	0.645	1.10E-02
RTTN	-0.711	8.90E-03	*FREM1*	0.460	1.10E-02
TLR1	1.791	9.00E-03	*TAS2R8*	-0.493	1.10E-02
ADRB2	0.827	9.00E-03	*ZMYND12*	-0.510	1.10E-02
IL20RA	-0.634	9.00E-03	*DSTYK*	-0.515	1.10E-02
KIAA0319	-0.635	9.00E-03	*TAF2*	-0.565	1.10E-02
CCDC30	-0.661	9.00E-03	*CCDC80*	1.070	1.11E-02
CNKSR1	-0.681	9.00E-03	*C3*	0.883	1.11E-02
TMEM54	-0.712	9.00E-03	*LIMA1*	0.860	1.11E-02
UPP2	-0.721	9.00E-03	*EHD4*	0.850	1.11E-02
ELMO3	-0.732	9.00E-03	*FLJ40852*	-0.453	1.11E-02
LETM1	-0.755	9.00E-03	*TCHP*	-0.483	1.11E-02
LAP3	1.875	9.10E-03	*LONRF2*	-0.503	1.11E-02
HLA-DPB1	1.632	9.10E-03	*FANCF*	-0.528	1.11E-02
RUNX3	1.489	9.10E-03	*IFI16*	1.078	1.12E-02
TMEM176B	1.185	9.10E-03	*FTL*	0.948	1.12E-02
FAM105A	0.940	9.10E-03	*BEND7*	-0.437	1.12E-02
PPIL6	-0.570	9.10E-03	*FAM84B*	-0.450	1.12E-02
YBEY	-0.650	9.10E-03	*EBF1*	0.863	1.13E-02

ZNF76	-0.755	9.10E-03	*FPR3*	0.993	1.14E-02
EMR2	1.382	9.20E-03	*ROR2*	0.595	1.14E-02
GNG2	1.355	9.20E-03	*LEP*	0.510	1.14E-02
PNOC	1.058	9.20E-03	*RBBP9*	-0.425	1.14E-02
ZDHHC15	-0.576	9.20E-03	*ZNF252P-AS1*	-0.507	1.14E-02
SERPINB11	-0.600	9.20E-03	*C11orf49*	-0.518	1.14E-02
PIEZO2	-0.618	9.20E-03	*OLFML1*	0.978	1.15E-02
SLC4A3	-0.711	9.20E-03	*ALOX5AP*	0.950	1.15E-02
HCLS1	1.853	9.30E-03	*ADCY7*	0.898	1.15E-02
SIGLEC10	1.624	9.30E-03	*CXorf21*	0.743	1.15E-02
GGTA1P	1.249	9.30E-03	*NEK6*	0.680	1.15E-02
CD226	1.128	9.30E-03	*ZNF77*	-0.430	1.15E-02
RSPH4A	-0.580	9.30E-03	*GJA9*	-0.475	1.15E-02
LOC728819	-0.618	9.30E-03	*ANO8*	-0.517	1.15E-02
TNNI3	-0.667	9.30E-03	*POLG2*	-0.538	1.15E-02
DTWD2	-0.690	9.30E-03	*SPARCL1*	0.938	1.16E-02
TIGD3	-0.730	9.30E-03	*HSD11B1*	0.640	1.16E-02
IFI44	1.870	9.40E-03	*MMP3*	0.485	1.16E-02
LACC1	1.351	9.40E-03	*EYA3*	-0.435	1.16E-02
ELMO1	1.057	9.40E-03	*GRTP1*	-0.470	1.16E-02
ALDH1A1	0.894	9.40E-03	*PRUNE*	-0.498	1.16E-02
CACNG1	-0.754	9.40E-03	*SLC25A15*	-0.520	1.16E-02
ARHGAP15	1.716	9.50E-03	*TXNL4A*	-0.543	1.16E-02
MS4A4A	1.663	9.50E-03	*PUS7*	-0.568	1.16E-02
SMAP2	1.382	9.50E-03	*LBH*	0.680	1.17E-02
LY75	1.346	9.50E-03	*ADRBK2*	0.578	1.17E-02
CCL13	1.345	9.50E-03	*TRIM6*	0.513	1.17E-02
COQ6	-0.657	9.50E-03	*TRABD2A*	0.483	1.17E-02
UBE2D4	-0.706	9.50E-03	*RANBP17*	-0.428	1.17E-02
LGMN	1.295	9.60E-03	*CD14*	1.040	1.18E-02
RGS1	1.207	9.60E-03	*CCL8*	0.723	1.18E-02
RASGRP3	1.191	9.60E-03	*IQGAP2*	0.628	1.18E-02
HOTAIR	-0.489	9.60E-03	*FAM176C*	0.420	1.18E-02
UTY	-0.512	9.60E-03	*ELOVL4*	-0.440	1.18E-02
VSIG10	-0.621	9.60E-03	*PROKR2*	-0.500	1.18E-02
PRF1	1.734	9.70E-03	*NNMT*	0.955	1.19E-02
RTP4	1.657	9.70E-03	*SYT11*	0.780	1.19E-02
ARL4C	1.090	9.70E-03	*ITGBL1*	0.763	1.19E-02
TMEM241	-0.586	9.70E-03	*HOXB2*	0.373	1.19E-02
LGR6	-0.591	9.70E-03	*XPNPEP3*	-0.425	1.19E-02
GREB1L	-0.698	9.70E-03	*CYBB*	1.030	1.20E-02
IKZF1	1.814	9.80E-03	*ZNF490*	-0.457	1.20E-02
SP140L	1.377	9.80E-03	*BSPRY*	-0.488	1.20E-02
FMNL2	1.131	9.80E-03	*SPC25*	-0.568	1.21E-02
GFI1	1.120	9.80E-03	*SAPCD1*	-0.580	1.21E-02
CASP8	1.118	9.80E-03	*GPR183*	0.870	1.22E-02
HLA-DQB2	1.044	9.80E-03	*PCLO*	-0.530	1.22E-02
ROBO1	0.997	9.80E-03	*IL7R*	0.895	1.23E-02
KCNRG	-0.529	9.80E-03	*CYTIP*	0.895	1.23E-02
ABHD12B	-0.530	9.80E-03	*PROS1*	0.878	1.23E-02
ARSG	-0.570	9.80E-03	*ABCA6*	0.843	1.23E-02

ARHGEF5	-0.607	9.80E-03	*CHSY3*	0.667	1.23E-02
FNTB	-0.639	9.80E-03	*KLC4*	-0.527	1.23E-02
TMEM18	-0.655	9.80E-03	*LSP1*	0.993	1.24E-02
MYO5C	-0.681	9.80E-03	*ADAM12*	0.925	1.24E-02
TP53TG1	-0.772	9.80E-03	*TOX2*	0.430	1.24E-02
CYB5D2	-0.780	9.80E-03	*C2orf15*	-0.453	1.24E-02
OAS2	1.794	9.90E-03	*B4GALT3*	-0.473	1.24E-02
IL18BP	1.581	9.90E-03	*FAM198B*	0.868	1.25E-02
LOC285548	-0.590	9.90E-03	*CD28*	0.793	1.25E-02
RADIL	-0.655	9.90E-03	*ALDH2*	0.793	1.25E-02
CMTM4	-0.661	9.90E-03	*TIE1*	0.658	1.25E-02
C16orf92	-0.733	9.90E-03	*MTMR7*	-0.403	1.25E-02
PARP15	1.164	1.00E-02	*REG1A*	-0.420	1.25E-02
ST3GAL5	0.995	1.00E-02	*NSUN4*	-0.440	1.25E-02
DDX3Y	-0.507	1.00E-02	*MLLT4-AS1*	-0.540	1.25E-02
FAM84B	-0.627	1.00E-02	*CDH5*	0.795	1.26E-02
CD163	1.740	1.01E-02	*VCAM1*	0.778	1.26E-02
CXCR6	1.710	1.01E-02	*ST6GAL1*	0.623	1.26E-02
CECR1	1.510	1.01E-02	*CAV1*	0.938	1.27E-02
TRIM14	1.244	1.01E-02	*EMR2*	0.780	1.27E-02
DUSP2	0.934	1.01E-02	*TDRD9*	0.213	1.27E-02
TNFSF4	0.879	1.01E-02	*SFT2D3*	-0.470	1.27E-02
LRRK1	0.879	1.01E-02	*KPTN*	-0.488	1.27E-02
KLRF1	0.695	1.01E-02	*SMG7*	-0.513	1.27E-02
CLDN23	0.634	1.01E-02	*CD4*	1.028	1.29E-02
SLC35F3	-0.512	1.01E-02	*CLEC12A*	0.663	1.29E-02
ZNF34	-0.628	1.01E-02	*PLIN2*	0.583	1.29E-02
ANO10	-0.655	1.01E-02	*UNC5CL*	-0.473	1.29E-02
FHIT	-0.661	1.01E-02	*HOXC11*	-0.488	1.29E-02
TRAK1	-0.693	1.01E-02	*SALL3*	-0.503	1.29E-02
IFT43	-0.745	1.01E-02	*C7orf53*	-0.513	1.29E-02
MIRLET7BHG	-0.750	1.01E-02	*ZNF653*	-0.517	1.29E-02
SLA	1.885	1.02E-02	*AXL*	1.013	1.30E-02
SP140	1.625	1.02E-02	*SYCP2*	-0.410	1.30E-02
GIMAP8	1.610	1.02E-02	*LINC00338*	-0.475	1.30E-02
IRX3	-0.587	1.02E-02	*FAM161A*	-0.553	1.30E-02
ZBTB7C	-0.618	1.02E-02	*PAFAH1B3*	-0.565	1.30E-02
GPHN	-0.665	1.02E-02	*LRMP*	0.848	1.31E-02
SEPT6	1.344	1.03E-02	*GM2A*	0.845	1.31E-02
TMCC3	0.851	1.03E-02	*GFPT2*	0.835	1.31E-02
PRR5L	0.749	1.03E-02	*JAM3*	0.770	1.31E-02
FBXO16	-0.599	1.03E-02	*CXCL14*	0.548	1.31E-02
FAM120AOS	-0.601	1.03E-02	*ELAVL2*	-0.495	1.31E-02
SOX12	-0.748	1.03E-02	*GPSM2*	-0.505	1.31E-02
ITGAM	1.796	1.04E-02	*MYL6B*	-0.543	1.31E-02
GVINP1	1.701	1.04E-02	*GIMAP5*	0.995	1.32E-02
RAB27A	1.447	1.04E-02	*CSF2RB*	0.905	1.32E-02
SYNGAP1	-0.773	1.04E-02	*CD200*	0.620	1.32E-02
CCR1	1.834	1.05E-02	*EOMES*	0.580	1.32E-02
GAB3	1.531	1.05E-02	*LANCL2*	-0.458	1.32E-02
GZMH	1.489	1.05E-02	*DHCR7*	-0.483	1.32E-02

PRKCH	1.184	1.05E-02	*LOC728485*	-0.537	1.32E-02
CSTA	1.098	1.05E-02	*LY96*	1.098	1.33E-02
RARRES1	0.982	1.05E-02	*LRRK2*	0.837	1.33E-02
TRAF3	0.887	1.05E-02	*LOC339524*	0.780	1.33E-02
ANKDD1A	0.781	1.05E-02	*ACSL5*	0.690	1.33E-02
C8orf47	0.389	1.05E-02	*KDR*	0.648	1.33E-02
FONG	0.341	1.05E-02	*CPM*	0.548	1.33E-02
SCGB1D1	-0.589	1.05E-02	*HIST1H4F*	-0.383	1.33E-02
COBL	-0.633	1.05E-02	*XYLB*	-0.440	1.33E-02
C5	-0.668	1.05E-02	*RABL5*	-0.470	1.33E-02
DPCD	-0.709	1.05E-02	*GMFG*	0.985	1.34E-02
FAM98C	-0.766	1.05E-02	*PRRX1*	0.900	1.34E-02
ARNTL2	1.362	1.06E-02	*SUSD1*	0.687	1.34E-02
IFNAR2	1.048	1.06E-02	*APOL4*	0.517	1.34E-02
TESPA1	0.972	1.06E-02	*LRRCC1*	-0.513	1.34E-02
MUC15	0.502	1.06E-02	*NOP56*	-0.573	1.34E-02
PKHD1L1	0.372	1.06E-02	*MXRA5*	0.903	1.35E-02
MAGEA4	0.315	1.06E-02	*PTPN22*	0.848	1.35E-02
EML6	-0.573	1.06E-02	*DARC*	0.810	1.35E-02
SNORD8	-0.595	1.06E-02	*CYP1B1*	0.703	1.35E-02
FAM222A-AS1	-0.621	1.06E-02	*CXCL13*	0.545	1.35E-02
SFT2D3	-0.648	1.06E-02	*C10orf12*	-0.435	1.35E-02
KAZALD1	-0.671	1.06E-02	*HIST1H4G*	-0.460	1.35E-02
MFSD3	-0.762	1.06E-02	*THAP9*	-0.513	1.35E-02
ISG20	1.639	1.07E-02	*HAUS8*	-0.537	1.35E-02
CXCR4	1.316	1.07E-02	*FHL1*	0.778	1.36E-02
LRRC6	-0.536	1.07E-02	*HPGDS*	0.678	1.36E-02
RBM20	-0.548	1.07E-02	*NR1H3*	0.648	1.36E-02
CSPG5	-0.617	1.07E-02	*IGDCC4*	0.547	1.36E-02
GDPD3	-0.624	1.07E-02	*IGLL3P*	0.525	1.36E-02
ULK4	-0.643	1.07E-02	*ABCB1*	0.495	1.36E-02
GPR18	1.320	1.08E-02	*ZSCAN5A*	-0.423	1.36E-02
ERP27	0.598	1.08E-02	*KIAA2022*	-0.463	1.36E-02
PROM1	0.502	1.08E-02	*KSR2*	-0.467	1.36E-02
SETD3	-0.578	1.08E-02	*FP588*	-0.553	1.36E-02
LDHC	-0.582	1.08E-02	*SLAMF8*	0.985	1.37E-02
TUBG2	-0.655	1.08E-02	*GPRIN3*	0.453	1.37E-02
KISS1R	-0.661	1.08E-02	*IQCD*	-0.463	1.37E-02
ATP9A	-0.737	1.08E-02	*CLVS1*	-0.470	1.37E-02
CD1D	0.939	1.09E-02	*ZNF491*	-0.477	1.37E-02
ALPK2	0.875	1.09E-02	*PYCRL*	-0.555	1.37E-02
ST8SIA1	0.641	1.09E-02	*CCR5*	0.938	1.38E-02
RGMB	-0.586	1.09E-02	*ALOX5*	0.893	1.38E-02
CAPN8	-0.597	1.09E-02	*C1orf100*	-0.467	1.38E-02
SLC41A3	-0.639	1.09E-02	*SNRPC*	-0.563	1.38E-02
NPAS2	-0.645	1.09E-02	*OXLD1*	-0.563	1.38E-02
CACNB2	-0.679	1.09E-02	*HIST1H2BI*	-0.405	1.39E-02
LYZL4	-0.741	1.09E-02	*GLS2*	-0.465	1.39E-02
ZNF397	-0.748	1.09E-02	*FAHD2A*	-0.513	1.39E-02
FLJ45983	-0.769	1.09E-02	*CUX2*	0.293	1.40E-02
CD247	1.611	1.10E-02	*HIST1H3A*	-0.393	1.40E-02

CFHR2	0.785	1.10E-02	*LCN12*	-0.413	1.40E-02
TIMD4	0.624	1.10E-02	*C3orf37*	-0.473	1.40E-02
ARHGAP36	-0.586	1.10E-02	*ARHGAP30*	1.020	1.41E-02
DDAH1	-0.643	1.10E-02	*CD68*	0.935	1.41E-02
C11orf52	-0.714	1.10E-02	*SERPINB9*	0.678	1.41E-02
SLC26A11	-0.733	1.10E-02	*KL*	0.455	1.41E-02
C2	1.637	1.11E-02	*TRAF3IP1*	-0.438	1.41E-02
TLR3	1.365	1.11E-02	*AIF1*	1.133	1.42E-02
STYK1	0.588	1.11E-02	*CCRL2*	0.783	1.42E-02
ZNF541	-0.682	1.11E-02	*PLXNC1*	0.780	1.42E-02
PSMB8	1.924	1.12E-02	*PLXDC2*	0.777	1.42E-02
IGSF6	1.640	1.12E-02	*ABCA9*	0.670	1.42E-02
LBH	1.068	1.12E-02	*GEMIN8*	-0.450	1.42E-02
SLC48A1	-0.714	1.12E-02	*TM4SF18*	0.583	1.43E-02
CUL7	-0.770	1.12E-02	*KLHL34*	-0.423	1.43E-02
TMEM71	1.015	1.13E-02	*HYLS1*	-0.490	1.43E-02
PLEKHG1	0.850	1.13E-02	*FXN*	-0.503	1.43E-02
HHEX	0.824	1.13E-02	*ADNP2*	-0.503	1.43E-02
SHROOM2	-0.633	1.13E-02	*CHRNA5*	-0.505	1.43E-02
ENPP7	-0.741	1.13E-02	*SEPT6*	0.763	1.44E-02
LHFPL2	1.291	1.14E-02	*SYK*	0.730	1.44E-02
CD83	1.284	1.14E-02	*FAM26F*	0.713	1.44E-02
MSL3	0.736	1.14E-02	*MARVELD2*	-0.453	1.44E-02
MFHAS1	0.679	1.14E-02	*COL5A2*	0.960	1.45E-02
FAM176C	0.548	1.14E-02	*FNDC1*	0.653	1.45E-02
EPYC	0.468	1.14E-02	*ELMO1*	0.608	1.45E-02
ACTRT3	0.430	1.14E-02	*SMC1B*	-0.397	1.45E-02
TCTN2	-0.564	1.14E-02	*LOC100271722*	-0.467	1.45E-02
CPO	-0.568	1.14E-02	*NSUN5P2*	-0.565	1.45E-02
CCDC147	-0.653	1.14E-02	*PTGDS*	0.863	1.46E-02
SOGA3	-0.687	1.14E-02	*TOR4A*	0.678	1.46E-02
PRLR	-0.705	1.14E-02	*ART3*	-0.375	1.46E-02
COX16	-0.713	1.14E-02	*WDR5*	-0.553	1.46E-02
UNC79	-0.726	1.14E-02	*GLIPR1*	1.018	1.47E-02
ARAP2	1.571	1.15E-02	*OLFML2B*	0.838	1.47E-02
PVRIG	1.502	1.15E-02	*BTNL9*	0.313	1.47E-02
LAX1	1.295	1.15E-02	*ST20*	-0.408	1.47E-02
TMEM45B	-0.625	1.15E-02	*HIST1H4E*	-0.408	1.47E-02
ASTN2	-0.632	1.15E-02	*IGSF3*	-0.443	1.47E-02
ANO8	-0.708	1.15E-02	*EPS8L2*	-0.498	1.47E-02
BTN3A1	1.794	1.16E-02	*PYHIN1*	0.680	1.48E-02
APOL6	1.749	1.16E-02	*MN1*	0.593	1.48E-02
PIK3CD	1.625	1.16E-02	*MSI2*	-0.400	1.48E-02
NFKBIE	1.514	1.16E-02	*ARNT2*	-0.415	1.48E-02
SLAMF1	1.429	1.16E-02	*IRF6*	-0.443	1.48E-02
MAF	1.064	1.16E-02	*FAM222A-AS1*	-0.447	1.48E-02
SPP1	0.689	1.16E-02	*PPCDC*	-0.473	1.48E-02
TLR8	1.706	1.17E-02	*CCNE2*	-0.543	1.48E-02
HLA-DOB	1.571	1.17E-02	*FBN1*	1.053	1.49E-02
SLC12A8	0.486	1.17E-02	*TXNIP*	0.918	1.49E-02
LOC283174	-0.655	1.17E-02	*VWF*	0.893	1.49E-02

CTNNBIP1	-0.714	1.17E-02	*DNMT3B*	-0.498	1.49E-02
NENF	-0.726	1.17E-02	*DNAJC27*	-0.517	1.49E-02
TAL2	-0.744	1.17E-02	*FOLR2*	1.088	1.50E-02
SIRPG	1.411	1.18E-02	*AOAH*	0.913	1.50E-02
PARP8	1.248	1.18E-02	*PAPLN*	0.597	1.50E-02
TPST2	0.974	1.18E-02	*NIM1*	-0.430	1.50E-02
HSD11B1	0.920	1.18E-02	*PCYT2*	-0.538	1.50E-02
CCDC83	-0.506	1.18E-02	*ECM2*	0.948	1.51E-02
DNMBP	-0.582	1.18E-02	*TREM2*	0.838	1.51E-02
MS4A6A	1.908	1.19E-02	*CCL4*	0.828	1.51E-02
APOE	1.258	1.19E-02	*TSPAN7*	0.673	1.51E-02
RASGRP1	1.046	1.19E-02	*RTN1*	0.533	1.51E-02
MT1H	0.576	1.19E-02	*MYB*	-0.475	1.51E-02
UNC13B	-0.609	1.19E-02	*LYRM2*	-0.480	1.51E-02
FLJ25758	-0.667	1.19E-02	*NTHL1*	-0.565	1.51E-02
GTDC2	-0.668	1.19E-02	*APOL1*	0.868	1.52E-02
PPM1K	1.159	1.20E-02	*DPYSL3*	0.803	1.52E-02
PLA1A	0.821	1.20E-02	*TMEM119*	0.777	1.52E-02
DPY19L2	-0.595	1.20E-02	*P2RY12*	0.453	1.52E-02
DNAH12	-0.666	1.20E-02	*PRRT3*	-0.450	1.52E-02
ALAD	-0.678	1.20E-02	*APOA1BP*	-0.560	1.52E-02
PROKR2	-0.686	1.20E-02	*RCSD1*	0.800	1.53E-02
DNAL4	-0.693	1.20E-02	*USF1*	-0.377	1.53E-02
CHD6	-0.777	1.20E-02	*SPINK13*	-0.380	1.53E-02
ADCY7	1.538	1.21E-02	*CASP4*	1.000	1.54E-02
OAS3	1.495	1.21E-02	*ARHGAP9*	0.983	1.54E-02
P2RY10	1.385	1.21E-02	*FERMT3*	0.923	1.54E-02
ACP5	1.101	1.21E-02	*SAMD3*	0.630	1.54E-02
PTPDC1	-0.653	1.21E-02	*RGS13*	0.360	1.54E-02
MILR1	1.045	1.22E-02	*AMICA1*	0.857	1.55E-02
BLNK	0.888	1.22E-02	*PPP1R16B*	0.738	1.55E-02
C15orf32	-0.586	1.22E-02	*SLC2A3*	0.818	1.56E-02
ALDH1L1	-0.608	1.22E-02	*ESAM*	0.677	1.56E-02
ALX1	-0.612	1.22E-02	*EMB*	0.580	1.56E-02
ARHGAP35	-0.698	1.22E-02	*RGL1*	0.923	1.57E-02
RFTN1	1.436	1.23E-02	*IL18BP*	0.830	1.57E-02
NOD2	1.148	1.23E-02	*ITIH5*	0.743	1.57E-02
ANKRD45	0.365	1.23E-02	*DYNC1I1*	-0.455	1.57E-02
ZNF287	-0.649	1.23E-02	*RNF133*	-0.463	1.57E-02
C14orf132	-0.751	1.23E-02	*PPP1R13B*	-0.490	1.57E-02
FCER1G	1.847	1.24E-02	*C5orf20*	0.620	1.58E-02
GZMK	1.622	1.24E-02	*HOXA5*	0.353	1.58E-02
NKG7	1.528	1.24E-02	*KLHL23*	-0.500	1.58E-02
KBTBD8	1.211	1.24E-02	*EARS2*	-0.520	1.58E-02
MZB1	1.206	1.24E-02	*IL1R1*	0.850	1.59E-02
PODXL	0.678	1.24E-02	*FAM78A*	0.807	1.59E-02
HES1	-0.557	1.24E-02	*MMRN2*	0.675	1.59E-02
PEX11A	-0.615	1.24E-02	*ANGPTL1*	0.663	1.59E-02
TMCO5A	-0.697	1.24E-02	*IGSF10*	0.537	1.59E-02
ERBB4	-0.699	1.24E-02	*RAB12*	-0.393	1.59E-02
KIF3A	-0.721	1.24E-02	*ZSCAN2*	-0.465	1.59E-02

PARP12	1.711	1.25E-02	*GLT8D2*	1.000	1.60E-02
LTB	1.685	1.25E-02	*KLF2*	0.728	1.60E-02
EGR2	1.043	1.25E-02	*ZNF319*	0.497	1.60E-02
C14orf37	-0.564	1.25E-02	*GPR133*	0.427	1.60E-02
ZNF445	-0.674	1.25E-02	*ZNF620*	-0.370	1.60E-02
TTC8	-0.731	1.25E-02	*TACC2*	-0.463	1.60E-02
MAGED2	-0.751	1.25E-02	*ZNF709*	-0.500	1.60E-02
CD52	1.660	1.26E-02	*TH1L*	-0.548	1.60E-02
IL27RA	1.256	1.26E-02	*PLEKHO2*	1.078	1.61E-02
IQCE	-0.645	1.26E-02	*THBD*	0.740	1.61E-02
LCK	1.637	1.27E-02	*VWA5A*	0.670	1.61E-02
NT5C3	0.862	1.27E-02	*FAM46C*	0.440	1.61E-02
A2ML1	0.587	1.27E-02	*MAB21L1*	0.410	1.61E-02
RGS13	0.498	1.27E-02	*GZMA*	0.760	1.62E-02
DYNC1I1	-0.637	1.27E-02	*RRN3P2*	0.360	1.62E-02
ARL8A	-0.669	1.27E-02	*CD163L1*	0.343	1.62E-02
C3AR1	1.784	1.28E-02	*GLYATL2*	0.240	1.62E-02
CYSLTR1	1.202	1.28E-02	*BCL11A*	-0.483	1.62E-02
IL31RA	-0.612	1.28E-02	*RCBTB2*	0.913	1.63E-02
SPON2	-0.661	1.28E-02	*TACC1*	0.863	1.63E-02
FBXO9	-0.667	1.28E-02	*FOXN3*	0.805	1.63E-02
LEAP2	-0.684	1.28E-02	*MMP13*	0.435	1.63E-02
DTX3	-0.783	1.28E-02	*C10orf113*	0.117	1.63E-02
SOD2	1.625	1.29E-02	*WDR88*	-0.420	1.63E-02
CD3G	1.443	1.29E-02	*C5orf55*	-0.420	1.63E-02
CPNE8	1.022	1.29E-02	*HRASLS*	-0.383	1.64E-02
CPNE5	0.807	1.29E-02	*BTBD17*	-0.497	1.64E-02
CHST9-AS1	-0.524	1.29E-02	*RCOR2*	-0.540	1.64E-02
OSBPL6	-0.535	1.29E-02	*BIN2*	0.935	1.65E-02
RIC8B	-0.608	1.29E-02	*PCDHB8*	-0.348	1.66E-02
RGS17	-0.625	1.29E-02	*FXYD4*	-0.417	1.66E-02
HLA-A	1.840	1.30E-02	*ZBTB42*	-0.497	1.66E-02
TNFRSF1B	1.743	1.30E-02	*CD52*	0.905	1.67E-02
MPP1	1.041	1.30E-02	*NEXN*	0.833	1.67E-02
PIP4K2A	0.902	1.30E-02	*OLA1*	-0.498	1.67E-02
SAYSD1	-0.620	1.30E-02	*MLKL*	0.863	1.68E-02
KANK4	-0.621	1.30E-02	*FOXP4*	-0.510	1.68E-02
LRTOMT	-0.644	1.30E-02	*MYO1F*	1.055	1.69E-02
ESRRG	-0.696	1.30E-02	*TGFBI*	0.878	1.69E-02
TLR7	1.427	1.31E-02	*LILRB5*	0.695	1.69E-02
P2RY6	1.194	1.31E-02	*TNFRSF17*	0.453	1.69E-02
MAL	0.734	1.31E-02	*CD300A*	0.965	1.70E-02
HIST1H2BB	-0.515	1.31E-02	*GNG11*	0.818	1.70E-02
SLITRK6	-0.547	1.31E-02	*HOXA10*	0.190	1.70E-02
FLJ38379	-0.604	1.31E-02	*HOXC13*	-0.418	1.70E-02
WDFY3-AS2	-0.672	1.31E-02	*CTDSPL*	-0.435	1.70E-02
IFITM1	1.591	1.32E-02	*OR7C1*	-0.515	1.70E-02
HERC6	1.541	1.32E-02	*PSMG3*	-0.557	1.70E-02
UBE2E3	0.928	1.32E-02	*TNFSF13B*	0.923	1.71E-02
RAB33A	0.745	1.32E-02	*OGN*	0.708	1.71E-02
NAGS	-0.614	1.32E-02	*TESPA1*	0.547	1.71E-02

KIAA0895	-0.660	1.32E-02	*SHROOM2*	-0.430	1.71E-02
GGT7	-0.732	1.32E-02	*RPL32P3*	-0.497	1.71E-02
DNAH7	-0.744	1.32E-02	*GIMAP2*	0.977	1.72E-02
TMC8	1.681	1.33E-02	*GUSBP11*	0.573	1.72E-02
ARSE	-0.578	1.33E-02	*CTBP2*	-0.450	1.72E-02
MANEAL	-0.610	1.33E-02	*SPPL3*	-0.487	1.72E-02
ZNF483	-0.643	1.33E-02	*CSRP2BP*	-0.490	1.72E-02
FAM116B	-0.763	1.33E-02	*GVINP1*	0.855	1.73E-02
ANKRD44	1.109	1.34E-02	*TMEM176B*	0.665	1.73E-02
BCAT1	0.872	1.34E-02	*AADAC*	0.358	1.73E-02
SLFN13	0.476	1.34E-02	*NKAPP1*	-0.410	1.73E-02
LIN28B	0.371	1.34E-02	*MLLT4*	-0.465	1.73E-02
FAM189A1	-0.659	1.34E-02	*RBM14*	-0.533	1.73E-02
RAC2	1.840	1.35E-02	*COL1A2*	0.970	1.74E-02
IL6R	0.996	1.35E-02	*CEP112*	0.683	1.74E-02
CXCR2	0.828	1.35E-02	*CD80*	0.663	1.74E-02
FAM179A	0.646	1.35E-02	*SH3RF3*	0.573	1.74E-02
MTSS1	-0.573	1.35E-02	*ANKDD1A*	0.490	1.74E-02
C19orf18	-0.628	1.35E-02	*C14orf39*	-0.353	1.74E-02
RALGPS1	-0.756	1.35E-02	*DNAL4*	-0.485	1.74E-02
IFIT2	1.669	1.36E-02	*C11orf45*	0.210	1.75E-02
IL7R	1.508	1.36E-02	*ZNF750*	-0.375	1.75E-02
RFX5	1.165	1.36E-02	*VCX*	-0.400	1.75E-02
TMEM156	0.768	1.36E-02	*DDX49*	-0.563	1.75E-02
PIH1D2	-0.569	1.36E-02	*ARHGDIB*	0.883	1.76E-02
C1orf56	-0.570	1.36E-02	*SOX17*	0.675	1.76E-02
SLC22A23	-0.687	1.36E-02	*SLC6A15*	-0.475	1.76E-02
IFIH1	1.855	1.37E-02	*PYROXD2*	-0.490	1.76E-02
PLEKHA2	0.890	1.37E-02	*TIMM13*	-0.543	1.76E-02
SCD5	0.588	1.37E-02	*HTRA1*	1.038	1.77E-02
TDRD5	-0.594	1.37E-02	*TMEM173*	0.830	1.77E-02
SLC1A2	-0.655	1.37E-02	*C11orf96*	0.663	1.77E-02
HOOK2	-0.687	1.37E-02	*CCDC23*	-0.440	1.77E-02
LOC643923	-0.760	1.37E-02	*CHRNB3*	-0.525	1.77E-02
AMICA1	1.503	1.38E-02	*SGPP1*	0.818	1.78E-02
TRAF1	1.371	1.38E-02	*SLFN5*	0.780	1.78E-02
FAM69A	1.128	1.38E-02	*SLC39A8*	0.463	1.78E-02
GPR114	1.086	1.38E-02	*ZSWIM5*	-0.460	1.78E-02
MYO5B	-0.620	1.38E-02	*SNHG10*	-0.497	1.78E-02
SOX13	-0.682	1.38E-02	*SASH3*	1.043	1.79E-02
ZFHX4	-0.684	1.38E-02	*TNFAIP8*	0.845	1.79E-02
DDR1	-0.731	1.38E-02	*MGLL*	0.695	1.79E-02
ALOX5AP	1.573	1.39E-02	*LMCD1*	0.685	1.79E-02
FAM107B	0.843	1.39E-02	*SOCS2*	0.380	1.79E-02
DAPK1	0.809	1.39E-02	*KCTD14*	-0.400	1.79E-02
LYPD1	0.561	1.39E-02	*C20orf96*	-0.437	1.79E-02
C6orf164	-0.589	1.39E-02	*PLK4*	-0.565	1.79E-02
VAV3	-0.594	1.39E-02	*NCF4*	0.985	1.80E-02
CASC2	-0.679	1.39E-02	*GBP3*	0.830	1.80E-02
CD74	1.769	1.40E-02	*SEMA5A*	0.778	1.80E-02
SAMD9	1.760	1.40E-02	*CLMP*	0.623	1.80E-02

P2RY13	1.557	1.40E-02	*IL18R1*	0.620	1.80E-02
LTF	0.638	1.40E-02	*MECOM*	0.420	1.80E-02
TMED6	-0.491	1.40E-02	*FUT4*	0.415	1.80E-02
MX1	1.593	1.41E-02	*DNASE1*	-0.408	1.80E-02
FAM65B	1.244	1.41E-02	*LOC646762*	-0.423	1.80E-02
AMPD3	1.023	1.41E-02	*RIC8B*	-0.430	1.80E-02
KIAA0125	1.003	1.41E-02	*RRP8*	-0.483	1.80E-02
ACSL6	0.406	1.41E-02	*EIF2B5*	-0.505	1.80E-02
RNF133	-0.634	1.41E-02	*SNX29*	0.747	1.81E-02
POMT1	-0.711	1.41E-02	*KLRF1*	0.415	1.81E-02
ITGAL	1.722	1.42E-02	*PDC*	-0.420	1.81E-02
FAM78A	1.386	1.42E-02	*OIP5*	-0.568	1.81E-02
SLC26A9	0.458	1.42E-02	*PCOLCE*	0.950	1.82E-02
ZNF490	-0.596	1.42E-02	*EMCN*	0.815	1.82E-02
LRRC17	-0.654	1.42E-02	*RAI2*	0.620	1.82E-02
CARKD	-0.715	1.42E-02	*GJA5*	0.608	1.82E-02
NOL3	-0.729	1.42E-02	*GUCY1B3*	0.600	1.82E-02
BTN3A3	1.896	1.43E-02	*GJB2*	0.397	1.82E-02
ZNF479	-0.542	1.43E-02	*CD74*	0.963	1.83E-02
C17orf78	-0.588	1.43E-02	*DRAM1*	0.940	1.83E-02
SETD4	-0.630	1.43E-02	*VAV1*	0.860	1.83E-02
MUM1	-0.705	1.43E-02	*FBXO22*	-0.505	1.83E-02
SHC2	-0.756	1.43E-02	*TNPO2*	-0.533	1.83E-02
FERMT3	1.638	1.44E-02	*RFTN1*	0.773	1.85E-02
CCR7	1.497	1.44E-02	*STAT5A*	0.735	1.85E-02
CD24	0.590	1.44E-02	*SPOCK1*	0.705	1.85E-02
DEFB1	0.571	1.44E-02	*SNTB2*	0.673	1.85E-02
ACSS3	-0.569	1.44E-02	*TDO2*	0.428	1.85E-02
LOC283588	-0.638	1.44E-02	*SCG2*	0.408	1.85E-02
TADA2B	-0.646	1.44E-02	*PCK1*	0.288	1.85E-02
PLEK	1.825	1.45E-02	*AAR2*	-0.460	1.85E-02
EVI2A	1.672	1.45E-02	*SPATA25*	-0.493	1.85E-02
CLEC2B	1.406	1.45E-02	*MMACHC*	-0.508	1.85E-02
APOBEC3C	1.114	1.45E-02	*SPARC*	1.010	1.86E-02
AKR1B1	1.054	1.45E-02	*EPB41L2*	0.680	1.86E-02
CDK20	-0.628	1.45E-02	*ADAMDEC1*	0.575	1.86E-02
CES4A	-0.710	1.45E-02	*LHFP*	0.943	1.87E-02
LOC441204	-0.764	1.45E-02	*PPAP2A*	0.815	1.87E-02
TEX264	-0.789	1.45E-02	*HS3ST2*	0.675	1.87E-02
LINC00189	-0.462	1.46E-02	*PARP15*	0.580	1.87E-02
AGR2	-0.536	1.46E-02	*CDO1*	0.560	1.87E-02
APOOL	-0.606	1.46E-02	*SIX4*	-0.423	1.87E-02
MAP2K5	-0.662	1.46E-02	*C18orf56*	-0.510	1.87E-02
CDC42SE2	1.668	1.47E-02	*NOM1*	-0.527	1.87E-02
PATL2	1.339	1.47E-02	*GPRIN1*	-0.560	1.87E-02
SLC9A7	0.592	1.47E-02	*UGT8*	-0.408	1.88E-02
PPEF1	-0.578	1.47E-02	*DIP2A*	-0.420	1.88E-02
HIST1H4G	-0.614	1.47E-02	*LOC144486*	-0.453	1.88E-02
DLG2	-0.649	1.47E-02	*FLJ25758*	-0.477	1.88E-02
ZBTB45	-0.702	1.47E-02	*INCENP*	-0.510	1.88E-02
SYT9	-0.713	1.47E-02	*DANCR*	-0.523	1.88E-02

ZNF428	-0.726	1.47E-02	*ANGPTL2*	1.020	1.89E-02
C21orf88	-0.750	1.47E-02	*MS4A7*	0.847	1.89E-02
PRKCQ	1.035	1.48E-02	*CTTN*	-0.415	1.89E-02
ADORA2A	0.811	1.48E-02	*FAM84A*	-0.420	1.89E-02
ABCB1	0.605	1.48E-02	*WDR67*	-0.538	1.89E-02
EEPD1	-0.569	1.48E-02	*SRPX2*	0.723	1.90E-02
PHF20	-0.609	1.48E-02	*NOX4*	0.463	1.90E-02
KCNE2	-0.671	1.48E-02	*PMF1*	-0.515	1.90E-02
AMN1	-0.704	1.48E-02	*CDH11*	0.938	1.91E-02
HAVCR2	1.633	1.49E-02	*FCN1*	0.878	1.91E-02
WNT8A	-0.531	1.49E-02	*LYVE1*	0.780	1.91E-02
WDR27	-0.724	1.49E-02	*CD3D*	0.735	1.91E-02
WIPF1	1.761	1.50E-02	*FBXO16*	-0.397	1.91E-02
KLRB1	1.461	1.50E-02	*DFFB*	-0.490	1.91E-02
LRMP	1.327	1.50E-02	*SHANK2*	-0.510	1.91E-02
BANK1	0.933	1.50E-02	*SLC41A2*	0.737	1.92E-02
FCRLA	0.799	1.50E-02	*PARM1*	0.543	1.92E-02
BTBD11	0.304	1.50E-02	*RBM4*	-0.467	1.92E-02
PCDH10	-0.615	1.50E-02	*LOC339352*	-0.517	1.92E-02
UBASH3B	0.858	1.51E-02	*PAQR4*	-0.520	1.92E-02
ORM2	0.360	1.51E-02	*SRPX*	0.845	1.93E-02
ZNF354C	-0.518	1.51E-02	*PHACTR2*	0.760	1.93E-02
CAPS2	-0.525	1.51E-02	*KLRK1*	0.715	1.93E-02
FOXP2	-0.631	1.51E-02	*SHE*	0.687	1.93E-02
SLFN12	1.106	1.52E-02	*PLBD1*	0.578	1.93E-02
FAM20A	0.981	1.52E-02	*AXDND1*	-0.387	1.93E-02
RAB37	0.873	1.52E-02	*ALX1*	-0.428	1.93E-02
SNTN	-0.520	1.52E-02	*ESCO2*	-0.540	1.93E-02
ARMC12	-0.637	1.52E-02	*ECSCR*	0.737	1.94E-02
PLXNB3	-0.713	1.52E-02	*GALNT12*	0.343	1.94E-02
MZT2A	-0.717	1.52E-02	*KLF5*	-0.460	1.94E-02
UBASH3A	1.235	1.53E-02	*FGD2*	0.957	1.95E-02
CCR6	0.628	1.53E-02	*TLR8*	0.888	1.95E-02
HOPX	0.578	1.53E-02	*ABHD6*	0.488	1.95E-02
SDK1	0.491	1.53E-02	*CUZD1*	-0.440	1.95E-02
DUSP15	-0.744	1.53E-02	*BCL9*	-0.463	1.95E-02
NCKAP1L	1.761	1.54E-02	*COQ7*	-0.465	1.95E-02
CST7	1.586	1.54E-02	*PALD1*	0.353	1.96E-02
LILRB4	1.412	1.54E-02	*YBEY*	-0.433	1.96E-02
MIAT	1.238	1.54E-02	*TCF20*	-0.470	1.96E-02
PLCG2	1.147	1.54E-02	*UBXN2B*	-0.478	1.96E-02
SYT11	1.129	1.54E-02	*LOC284023*	-0.480	1.96E-02
LOC100130100	1.033	1.54E-02	*C10orf54*	1.030	1.97E-02
ZSWIM1	-0.581	1.54E-02	*DDX43*	0.120	1.97E-02
PGLYRP2	-0.602	1.54E-02	*TTTY10*	-0.400	1.97E-02
AUTS2	-0.624	1.54E-02	*GLB1L3*	0.137	1.98E-02
ZNF683	1.431	1.55E-02	*C7orf26*	-0.505	1.98E-02
PAQR8	0.764	1.55E-02	*PIK3CG*	0.740	1.99E-02
MAGEA12	0.345	1.55E-02	*RAB20*	0.605	1.99E-02
GRIK2	-0.635	1.55E-02	*PLEKHA2*	0.568	1.99E-02
C16orf48	-0.719	1.55E-02	*ITGAL*	0.908	2.00E-02

LYN	1.587	1.56E-02	*CCL19*	0.665	2.00E-02
AP1S2	1.202	1.56E-02	*NLRC5*	0.833	2.01E-02
TSPAN33	0.726	1.56E-02	*SELP*	0.820	2.01E-02
IQCG	0.599	1.56E-02	*IGFBP3*	0.625	2.01E-02
CRISP3	-0.458	1.56E-02	*SALL1*	0.213	2.01E-02
CNIH3	-0.502	1.56E-02	*TCAM1P*	-0.360	2.01E-02
CKMT2	-0.559	1.56E-02	*ENDOV*	-0.473	2.01E-02
HILPDA	-0.562	1.56E-02	*MUCL1*	0.287	2.02E-02
UNC80	-0.638	1.56E-02	*ZNF233*	-0.397	2.02E-02
ENDOV	-0.660	1.56E-02	*RAB11FIP4*	-0.483	2.02E-02
ARHGAP30	1.740	1.57E-02	*GNG4*	-0.495	2.02E-02
ETS1	1.410	1.57E-02	*CTSS*	1.055	2.03E-02
EPB41L3	0.962	1.57E-02	*TMEM140*	0.955	2.03E-02
CCL7	0.931	1.57E-02	*FUCA1*	0.905	2.03E-02
LOC148709	-0.540	1.57E-02	*CARD16*	0.857	2.03E-02
NPY1R	-0.549	1.57E-02	*SEMA3G*	0.658	2.03E-02
REPS2	-0.595	1.57E-02	*RASGRP3*	0.628	2.03E-02
TBC1D16	-0.623	1.57E-02	*BST1*	0.625	2.03E-02
ZNF827	-0.641	1.57E-02	*ZNF174*	-0.433	2.03E-02
CYTH2	-0.668	1.57E-02	*ABCA8*	0.745	2.04E-02
AKAP1	-0.716	1.57E-02	*LRRN4CL*	0.740	2.04E-02
GPR84	1.288	1.58E-02	*ICOS*	0.615	2.04E-02
GAPT	0.996	1.58E-02	*TIGIT*	0.530	2.04E-02
ANXA2R	0.699	1.58E-02	*C10orf11*	0.530	2.04E-02
TNFSF15	0.560	1.58E-02	*ZBED2*	0.348	2.04E-02
DNAJA4	-0.503	1.58E-02	*LAMA1*	0.237	2.04E-02
CLSTN2	-0.627	1.58E-02	*CD300LF*	0.847	2.05E-02
PRKCZ	-0.646	1.58E-02	*CMPK2*	0.623	2.05E-02
KIAA1522	-0.697	1.58E-02	*BCL11B*	0.453	2.05E-02
OAS1	1.560	1.59E-02	*PTCD1*	-0.475	2.05E-02
NEK6	0.932	1.59E-02	*MIR600HG*	-0.508	2.05E-02
SLC34A2	0.603	1.59E-02	*SELL*	0.775	2.06E-02
FLJ40852	-0.583	1.59E-02	*GAPT*	0.610	2.06E-02
ST7L	-0.584	1.59E-02	*ERMN*	0.283	2.06E-02
NDNF	-0.602	1.59E-02	*KIAA1328*	-0.387	2.06E-02
TMEM120B	-0.646	1.59E-02	*TULP3*	-0.430	2.06E-02
S100A8	1.095	1.60E-02	*FYB*	0.945	2.07E-02
CXorf38	0.963	1.60E-02	*TGM2*	0.793	2.07E-02
SCG3	-0.513	1.60E-02	*MDFIC*	0.710	2.07E-02
EFCAB6	-0.636	1.60E-02	*GBP5*	0.790	2.08E-02
HLA-C	1.893	1.61E-02	*MAN1A1*	0.738	2.08E-02
APBB1IP	1.419	1.61E-02	*CD84*	0.933	2.09E-02
CD40	1.304	1.61E-02	*JAM2*	0.858	2.09E-02
CMTM7	0.864	1.61E-02	*TLR2*	0.813	2.09E-02
CD200	0.800	1.61E-02	*RHOU*	0.387	2.09E-02
ANKRD30A	-0.559	1.61E-02	*FAM120C*	-0.383	2.09E-02
TRIM21	1.513	1.62E-02	*DTYMK*	-0.565	2.09E-02
C11orf71	0.740	1.62E-02	*MEF2C*	0.778	2.10E-02
IQCD	-0.607	1.62E-02	*FGF7*	0.590	2.10E-02
HSD11B2	-0.614	1.62E-02	*FKBP11*	0.533	2.10E-02
N6AMT1	-0.618	1.62E-02	*ACVR1C*	0.353	2.10E-02

LOC339352	-0.718	1.62E-02	*SDK1*	0.333	2.10E-02
SASH3	1.858	1.63E-02	*ITK*	0.733	2.11E-02
GABRP	0.766	1.63E-02	*ZNF300P1*	0.327	2.11E-02
ARMC3	-0.613	1.63E-02	*EPYC*	0.273	2.11E-02
ABCG5	-0.637	1.63E-02	*IPO4*	-0.553	2.11E-02
MANSC1	-0.666	1.63E-02	*LCP1*	0.858	2.12E-02
HCST	1.611	1.64E-02	*EGFLAM*	0.490	2.12E-02
SLFN5	1.313	1.64E-02	*ANO4*	-0.383	2.12E-02
ICAM2	1.176	1.64E-02	*BTBD9*	-0.467	2.12E-02
OSBPL3	0.949	1.64E-02	*HBZ*	-0.390	2.13E-02
PAPLN	0.797	1.64E-02	*RASSF4*	0.728	2.14E-02
LINC00310	-0.492	1.64E-02	*MIAT*	0.710	2.14E-02
ZNF620	-0.496	1.64E-02	*SPRY1*	0.595	2.14E-02
NEFM	-0.531	1.64E-02	*C9orf84*	0.317	2.14E-02
HOOK1	-0.658	1.64E-02	*MICAL3*	-0.435	2.14E-02
ELP2	-0.690	1.64E-02	*DNAH11*	-0.443	2.14E-02
GMFG	1.641	1.65E-02	*ZNF124*	-0.458	2.14E-02
C17orf97	-0.525	1.65E-02	*CBY1*	-0.470	2.14E-02
NAT8	-0.582	1.65E-02	*DDR1*	-0.500	2.14E-02
NME3	-0.769	1.65E-02	*TAF5*	-0.538	2.14E-02
FAM49A	1.059	1.66E-02	*GAB3*	0.743	2.15E-02
FMO1	1.035	1.66E-02	*PAX6*	-0.365	2.15E-02
SYTL3	0.721	1.66E-02	*ZFP64*	-0.395	2.15E-02
HLA-DQA1	0.639	1.66E-02	*EFNA4*	-0.458	2.15E-02
SLC47A1	0.354	1.66E-02	*PPP1R9A*	-0.473	2.15E-02
NLRP2	0.261	1.66E-02	*SH2D6*	-0.473	2.15E-02
MAEL	0.248	1.66E-02	*CCL5*	0.845	2.16E-02
GDF11	-0.587	1.66E-02	*NABP1*	0.813	2.16E-02
ZNF552	-0.587	1.66E-02	*POU2AF1*	0.500	2.16E-02
TSHZ1	-0.699	1.66E-02	*CCDC149*	-0.443	2.16E-02
OSBPL5	-0.704	1.66E-02	*LOC728537*	-0.467	2.17E-02
POLRMT	-0.770	1.66E-02	*CD96*	0.705	2.19E-02
HVCN1	1.272	1.67E-02	*FAM20A*	0.597	2.19E-02
RASGEF1B	0.627	1.67E-02	*HAO1*	-0.478	2.19E-02
CCL26	0.518	1.67E-02	*PLVAP*	0.718	2.20E-02
IGF2BP2	0.511	1.67E-02	*GCET2*	0.423	2.20E-02
CA5A	-0.577	1.67E-02	*VSIG10*	-0.395	2.20E-02
PRKCB	1.365	1.68E-02	*MEIG1*	-0.403	2.20E-02
LPIN2	0.947	1.68E-02	*NMU*	-0.420	2.20E-02
KCNE3	0.544	1.68E-02	*DSN1*	-0.515	2.20E-02
C20orf202	-0.700	1.68E-02	*AKR1C3*	0.485	2.21E-02
SP100	1.634	1.69E-02	*HBA2*	0.295	2.21E-02
IFIT1	1.436	1.69E-02	*PCDHA5*	-0.405	2.21E-02
CTSL1	1.405	1.69E-02	*DBIL5P*	-0.407	2.21E-02
CD72	1.083	1.69E-02	*TAF6*	-0.483	2.21E-02
TOR4A	0.961	1.69E-02	*TSEN34*	-0.485	2.21E-02
SUSD1	0.953	1.69E-02	*FGR*	0.953	2.22E-02
IRAK2	0.870	1.69E-02	*RAC2*	0.943	2.22E-02
LEF1	0.584	1.69E-02	*STEAP4*	0.518	2.22E-02
MGC39372	0.451	1.69E-02	*FUT11*	0.457	2.22E-02
EGF	-0.478	1.69E-02	*ERP27*	0.333	2.22E-02

MOK	-0.496	1.69E-02	*ZFAT*	-0.367	2.22E-02
IQUB	-0.582	1.69E-02	*SIRT5*	-0.483	2.22E-02
TBC1D19	-0.690	1.69E-02	*HJURP*	-0.553	2.22E-02
MGMT	-0.696	1.69E-02	*ZFPM2*	0.643	2.23E-02
GPR183	1.317	1.70E-02	*ARRDC4*	0.607	2.23E-02
PIK3CG	1.258	1.70E-02	*SCG5*	0.333	2.23E-02
SLC2A6	1.199	1.70E-02	*SOX13*	-0.470	2.23E-02
VAX2	-0.651	1.70E-02	*PTRHD1*	-0.490	2.23E-02
BCORL1	-0.703	1.70E-02	*LMBR1*	-0.510	2.23E-02
BST2	1.296	1.71E-02	*THBS2*	0.803	2.24E-02
TM6SF1	1.227	1.71E-02	*NCEH1*	0.783	2.24E-02
CD28	1.125	1.71E-02	*TMEM220*	0.303	2.24E-02
FAM43A	0.588	1.71E-02	*OGDHL*	-0.430	2.24E-02
HS3ST3A1	0.572	1.71E-02	*MMP2*	0.960	2.25E-02
P2RY12	0.543	1.71E-02	*JAZF1*	0.740	2.25E-02
RAP1GAP2	-0.520	1.71E-02	*CD69*	0.723	2.25E-02
NIPAL3	-0.616	1.71E-02	*BRF2*	-0.428	2.25E-02
SLC29A1	-0.649	1.71E-02	*SLC35D3*	0.103	2.26E-02
ISG15	1.435	1.72E-02	*LOC100288911*	-0.467	2.26E-02
GPR34	1.129	1.72E-02	*FAM111B*	-0.480	2.26E-02
EFHB	-0.500	1.72E-02	*C16orf53*	-0.495	2.26E-02
BRF2	-0.601	1.72E-02	*LOC284440*	-0.500	2.26E-02
BSCL2	-0.713	1.72E-02	*PTCD3*	-0.538	2.26E-02
BTK	1.515	1.73E-02	*SAMD9L*	0.970	2.27E-02
C1orf54	1.202	1.73E-02	*GPR171*	0.650	2.27E-02
UCHL1	0.601	1.73E-02	*ADTRP*	0.305	2.27E-02
MICALCL	-0.585	1.73E-02	*STAP2*	-0.440	2.27E-02
KLHDC9	-0.717	1.73E-02	*NAPB*	-0.463	2.27E-02
PARP9	1.835	1.74E-02	*MARCKS*	0.720	2.28E-02
SH2D2A	1.253	1.74E-02	*TRAM2*	0.685	2.28E-02
SGK1	1.096	1.74E-02	*FAM65B*	0.653	2.28E-02
MAX	0.847	1.74E-02	*CHST11*	0.583	2.28E-02
ADRBK2	0.652	1.74E-02	*DAPK1*	0.500	2.28E-02
LINC00515	-0.521	1.75E-02	*SLC19A3*	0.488	2.28E-02
HOXC6	-0.532	1.75E-02	*IL12B*	0.325	2.28E-02
ZSCAN20	-0.567	1.75E-02	*ICA1L*	-0.417	2.28E-02
KIAA2022	-0.601	1.75E-02	*ZNF695*	-0.423	2.28E-02
GLRB	-0.658	1.75E-02	*CDPF1*	-0.447	2.28E-02
TMEM176A	1.109	1.76E-02	*CD48*	0.843	2.29E-02
ITM2A	0.939	1.76E-02	*AOX1*	0.645	2.29E-02
MAP3K14	0.888	1.76E-02	*TFPI2*	0.430	2.29E-02
MARC2	-0.567	1.76E-02	*SPATS2*	-0.418	2.29E-02
RBP2	-0.618	1.76E-02	*CC2D2A*	-0.440	2.29E-02
NFS1	-0.650	1.76E-02	*BHLHB9*	-0.445	2.29E-02
TMEM106C	-0.677	1.76E-02	*PLEK*	0.943	2.30E-02
TNRC18	-0.731	1.76E-02	*MIR155HG*	0.530	2.30E-02
KLRD1	1.461	1.77E-02	*ARHGEF9*	-0.448	2.30E-02
IL18RAP	1.431	1.77E-02	*ZNF282*	-0.525	2.30E-02
MARCH1	1.333	1.77E-02	*UFSP1*	-0.550	2.30E-02
LRRC8C	1.315	1.77E-02	*SLC6A6*	0.590	2.31E-02
ENPP2	1.031	1.77E-02	*C1orf114*	-0.388	2.31E-02

MMP3	0.513	1.77E-02	*ELK4*	-0.480	2.31E-02
PIGL	-0.563	1.77E-02	*FAM195B*	-0.493	2.31E-02
DIO1	-0.564	1.77E-02	*RAE1*	-0.520	2.31E-02
SLC7A8	-0.609	1.77E-02	*NUP133*	-0.560	2.31E-02
FCRL5	0.879	1.78E-02	*DKK2*	0.393	2.32E-02
KIAA1257	-0.550	1.78E-02	*ORAOV1*	-0.420	2.32E-02
GCOM1	-0.552	1.78E-02	*CPSF7*	-0.493	2.33E-02
CTNND2	-0.589	1.78E-02	*ADA*	0.480	2.34E-02
DET1	-0.663	1.78E-02	*AQPEP*	0.367	2.34E-02
THEMIS2	1.572	1.79E-02	*IDO2*	0.277	2.34E-02
VAV1	1.484	1.79E-02	*PHKA2*	-0.490	2.34E-02
CD27	1.383	1.79E-02	*MFAP4*	0.848	2.35E-02
AKNA	1.326	1.79E-02	*ADH1B*	0.700	2.35E-02
PTGS1	0.875	1.79E-02	*MYCT1*	0.660	2.35E-02
BASP1	0.808	1.79E-02	*MCOLN2*	0.373	2.35E-02
TPRG1	-0.470	1.79E-02	*RGNEF*	-0.415	2.35E-02
FAM120B	-0.603	1.79E-02	*CLEC4F*	-0.450	2.35E-02
FAM124A	-0.653	1.79E-02	*TTLL2*	-0.463	2.35E-02
LAMA5	-0.679	1.79E-02	*PATZ1*	-0.513	2.35E-02
NCF4	1.716	1.80E-02	*DGKE*	-0.400	2.36E-02
IL2RG	1.610	1.80E-02	*CDCA7L*	-0.483	2.36E-02
PTPN7	1.344	1.80E-02	*CILP*	0.838	2.37E-02
PPP1R16B	1.095	1.80E-02	*IL21R*	0.680	2.37E-02
CCDC69	0.992	1.80E-02	*PDCL2*	-0.320	2.37E-02
EPDR1	0.681	1.80E-02	*PIK3R2*	-0.533	2.37E-02
NINJ2	0.587	1.80E-02	*HVCN1*	0.693	2.38E-02
LOC100287628	0.531	1.80E-02	*PROCR*	0.475	2.38E-02
PCDH11X	-0.541	1.80E-02	*FCER1A*	0.375	2.38E-02
LPA	-0.577	1.80E-02	*RAB3C*	0.163	2.38E-02
TRAM1L1	-0.617	1.80E-02	*AP4M1*	-0.455	2.38E-02
SPATA4	-0.666	1.80E-02	*NCOA5*	-0.490	2.38E-02
DRAM1	1.654	1.81E-02	*PILRA*	0.790	2.39E-02
BIN2	1.582	1.81E-02	*CTSH*	0.695	2.39E-02
TNFAIP8L2	1.549	1.81E-02	*COMMD7*	-0.420	2.39E-02
LAT	1.362	1.81E-02	*CENPL*	-0.533	2.39E-02
TRAF3IP3	1.296	1.81E-02	*METTL10*	-0.537	2.39E-02
CAMK2D	1.185	1.81E-02	*LILRB4*	0.750	2.40E-02
ASS1	0.577	1.81E-02	*PNOC*	0.500	2.40E-02
ELMOD1	-0.499	1.81E-02	*GPR63*	-0.423	2.40E-02
CGREF1	-0.572	1.82E-02	*SLC22A16*	-0.430	2.40E-02
DLG5	-0.623	1.82E-02	*RABIF*	-0.455	2.40E-02
RBKS	-0.640	1.82E-02	*NT5C1B*	-0.467	2.40E-02
NAA60	-0.700	1.82E-02	*CDK2*	-0.493	2.40E-02
SPATA7	-0.726	1.82E-02	*C3orf75*	-0.523	2.40E-02
TLE4	0.977	1.83E-02	*WNT2*	0.808	2.41E-02
PLEKHF1	0.933	1.83E-02	*TAF5L*	-0.438	2.41E-02
C19orf12	0.576	1.83E-02	*BNC2*	0.813	2.42E-02
HOXA1	0.303	1.83E-02	*FAM26E*	0.647	2.42E-02
SH2B3	1.437	1.84E-02	*VASH1*	0.633	2.42E-02
CARD6	1.437	1.84E-02	*SLC36A1*	0.445	2.42E-02
MSANTD3	0.919	1.84E-02	*SEC61A2*	-0.418	2.42E-02

PLIN2	0.670	1.84E-02	*LSM14B*	-0.420	2.42E-02
RPS23	-0.635	1.84E-02	*CLCN2*	-0.453	2.42E-02
MANBAL	-0.701	1.84E-02	*ISYNA1*	-0.475	2.42E-02
TCEB2	-0.718	1.84E-02	*PLXDC1*	0.733	2.43E-02
HAGH	-0.738	1.84E-02	*PRSS23*	0.720	2.43E-02
HLA-E	1.990	1.85E-02	*PLEKHA8*	-0.350	2.43E-02
GALNT12	0.456	1.85E-02	*MORN3*	-0.403	2.43E-02
CCDC40	-0.715	1.85E-02	*CCR2*	0.830	2.44E-02
HS3ST1	0.360	1.86E-02	*CHN1*	0.538	2.44E-02
MTMR7	-0.507	1.86E-02	*THBS4*	0.423	2.44E-02
CES3	-0.634	1.86E-02	*KCNE3*	0.350	2.44E-02
EIF2D	-0.702	1.86E-02	*THSD7B*	0.223	2.44E-02
ADPGK	1.073	1.87E-02	*TPRG1*	0.163	2.44E-02
MSX2	-0.456	1.87E-02	*RPP14*	-0.448	2.44E-02
RNF43	-0.588	1.87E-02	*MYO5A*	0.788	2.45E-02
CHIA	-0.697	1.87E-02	*ETS1*	0.743	2.45E-02
STK17A	1.271	1.88E-02	*LIMS1*	0.543	2.45E-02
VSTM1	0.241	1.88E-02	*STIL*	-0.543	2.45E-02
NBR2	-0.588	1.88E-02	*GZMK*	0.788	2.46E-02
B2M	1.846	1.89E-02	*IL15RA*	0.713	2.46E-02
MAFB	1.212	1.89E-02	*CCDC3*	0.613	2.46E-02
CTSZ	1.106	1.89E-02	*TP53I3*	0.600	2.46E-02
AAR2	-0.619	1.89E-02	*C17orf89*	-0.530	2.46E-02
ARSD	-0.676	1.89E-02	*CMKLR1*	0.908	2.47E-02
SPOCK2	1.379	1.90E-02	*EFEMP1*	0.798	2.47E-02
RNF125	0.584	1.90E-02	*ARHGAP18*	0.767	2.47E-02
S100A7	0.333	1.90E-02	*CLECL1*	0.423	2.47E-02
TOM1L2	-0.760	1.90E-02	*ANKS1B*	-0.423	2.47E-02
NABP1	1.419	1.91E-02	*GTPBP5*	-0.467	2.47E-02
RHOH	1.111	1.91E-02	*DLC1*	0.730	2.48E-02
LMO2	0.865	1.91E-02	*NAPSB*	0.720	2.48E-02
CLEC5A	0.786	1.91E-02	*CADM2*	-0.400	2.48E-02
MMP20	0.527	1.91E-02	*SARNP*	-0.440	2.48E-02
PRAME	0.318	1.91E-02	*NOP14*	-0.518	2.48E-02
DDX53	-0.450	1.91E-02	*STK10*	0.920	2.49E-02
CMTM2	-0.638	1.91E-02	*LAMB1*	0.760	2.49E-02
PDZD4	-0.705	1.91E-02	*LGALS3*	0.745	2.49E-02
TMEM173	1.338	1.92E-02	*TOMM34*	-0.478	2.49E-02
FCRL1	0.587	1.92E-02	*GKAP1*	-0.530	2.49E-02
FRMD3	0.415	1.92E-02	*ICAM2*	0.648	2.50E-02
LYSMD1	-0.537	1.92E-02	*FLVCR2*	0.535	2.50E-02
LCP1	1.516	1.93E-02	*HIST1H2BA*	-0.303	2.50E-02
STAT5A	1.113	1.93E-02	*FANCC*	-0.488	2.50E-02
MOB3B	0.641	1.93E-02	*C19orf48*	-0.563	2.50E-02
MUC16	0.485	1.93E-02	*CHST15*	0.643	2.51E-02
JUP	-0.662	1.93E-02	*FMNL3*	0.607	2.51E-02
MARCO	1.121	1.94E-02	*TMEM71*	0.547	2.51E-02
IPCEF1	0.859	1.94E-02	*KLB*	0.273	2.51E-02
OR2L13	-0.452	1.94E-02	*BRD8*	-0.505	2.51E-02
EPHA6	-0.578	1.94E-02	*SMAP2*	0.653	2.52E-02
DZANK1	-0.639	1.94E-02	*PTPRJ*	0.553	2.52E-02

STX1A	-0.694	1.94E-02	*CCR6*	0.370	2.52E-02
SLC9A9	1.332	1.95E-02	*TBR1*	-0.445	2.52E-02
C3	1.293	1.95E-02	*MARCH9*	-0.470	2.52E-02
CD8B	0.971	1.95E-02	*YY1AP1*	-0.485	2.52E-02
KCNA3	0.867	1.95E-02	*NFKBIL1*	-0.545	2.52E-02
IL17RB	-0.533	1.95E-02	*GBP4*	0.703	2.53E-02
ZKSCAN2	-0.542	1.95E-02	*MAX*	0.525	2.53E-02
MYT1L	-0.576	1.95E-02	*RAB3IP*	-0.440	2.53E-02
LMLN	-0.599	1.95E-02	*ARHGAP24*	0.670	2.54E-02
CARD11	1.121	1.96E-02	*CLEC2D*	0.595	2.54E-02
IL1RAPL1	-0.539	1.96E-02	*LEF1*	0.358	2.54E-02
FAM19A2	-0.560	1.96E-02	*FAM188B*	-0.360	2.54E-02
DNM1P46	-0.627	1.96E-02	*C21orf88*	-0.523	2.54E-02
N6AMT2	-0.640	1.96E-02	*HMCN1*	0.600	2.55E-02
DDX58	1.441	1.97E-02	*NR5A2*	0.363	2.55E-02
NAPB	-0.640	1.97E-02	*C18orf62*	-0.463	2.55E-02
ZC2HC1A	-0.647	1.97E-02	*C18orf25*	-0.495	2.55E-02
RNF215	-0.675	1.97E-02	*PIF1*	-0.530	2.55E-02
GPSM1	-0.725	1.97E-02	*GINS1*	-0.550	2.55E-02
AXDND1	-0.517	1.98E-02	*IKZF1*	0.828	2.56E-02
EFCAB12	-0.595	1.98E-02	*FAM101B*	0.453	2.56E-02
CPA1	-0.603	1.98E-02	*SCML4*	0.540	2.57E-02
SFXN2	-0.665	1.98E-02	*IQCF1*	-0.440	2.57E-02
SP110	1.661	1.99E-02	*TTTY12*	-0.490	2.57E-02
CDH3	0.739	1.99E-02	*YAE1D1*	-0.517	2.57E-02
GTDC1	0.721	1.99E-02	*PRR7*	-0.518	2.57E-02
FUT11	0.618	1.99E-02	*F2R*	0.685	2.58E-02
CTXN1	-0.751	1.99E-02	*PLA1A*	0.470	2.58E-02
OXNAD1	1.066	2.00E-02	*TIMP4*	0.388	2.58E-02
PTPRJ	0.824	2.00E-02	*PLEKHH1*	-0.443	2.58E-02
ROPN1B	0.167	2.00E-02	*MPP6*	-0.475	2.58E-02
SH3BP4	-0.585	2.00E-02	*HMOX1*	0.695	2.59E-02
PCGF3	-0.601	2.00E-02	*SGCZ*	-0.367	2.59E-02
FLJ39653	-0.678	2.00E-02	*RND2*	-0.485	2.59E-02
MOSPD3	-0.753	2.00E-02	*MFNG*	0.695	2.60E-02
STK17B	1.610	2.01E-02	*CD247*	0.668	2.60E-02
SLAMF6	1.211	2.01E-02	*TRAT1*	0.570	2.60E-02
ZNF831	0.737	2.01E-02	*CIDEC*	0.498	2.60E-02
B3GNT1	-0.667	2.01E-02	*SEPT8*	0.470	2.60E-02
EGFL8	-0.733	2.01E-02	*AKR1C2*	0.298	2.60E-02
FAS	1.465	2.02E-02	*NLRP2*	0.133	2.60E-02
CCL28	0.344	2.02E-02	*TMEM216*	-0.393	2.60E-02
KLHL32	-0.494	2.02E-02	*KIAA0101*	-0.548	2.60E-02
OTUB2	-0.533	2.02E-02	*ZCCHC24*	0.873	2.61E-02
LRRN2	-0.682	2.02E-02	*GALC*	0.730	2.61E-02
RAB20	0.851	2.03E-02	*LPCAT2*	0.690	2.61E-02
FAXC	-0.572	2.03E-02	*LACC1*	0.657	2.61E-02
ACP6	-0.579	2.03E-02	*FEZ1*	0.625	2.61E-02
HLA-B	1.910	2.04E-02	*FGD5*	0.593	2.61E-02
NMI	1.822	2.04E-02	*FKBP5*	0.420	2.61E-02
IL12RB1	1.380	2.04E-02	*DLL1*	0.367	2.61E-02

FMO3	0.559	2.04E-02	*CCDC152*	0.297	2.61E-02
NMNAT1	-0.577	2.04E-02	*NLGN4X*	0.268	2.61E-02
RPL23AP32	-0.603	2.04E-02	*LOC100128893*	-0.437	2.61E-02
ZFP14	-0.632	2.04E-02	*MED25*	-0.495	2.61E-02
CTSW	1.000	2.05E-02	*THY1*	0.890	2.62E-02
CCL23	0.573	2.05E-02	*TGFB3*	0.783	2.62E-02
DNAJC22	-0.495	2.05E-02	*STX6*	-0.455	2.62E-02
ZNF434	-0.557	2.05E-02	*IFITM2*	0.810	2.63E-02
RPS6KL1	-0.664	2.05E-02	*COLEC12*	0.635	2.63E-02
MMP9	0.960	2.06E-02	*TAC1*	0.230	2.63E-02
AGPAT9	0.596	2.06E-02	*SLC29A2*	-0.500	2.63E-02
RDH10	0.555	2.06E-02	*EMX2*	0.340	2.64E-02
TIGD4	-0.580	2.06E-02	*HLA-DQA1*	0.375	2.65E-02
RILPL1	-0.645	2.06E-02	*LOC285696*	-0.417	2.65E-02
LCMT2	-0.646	2.06E-02	*RMND5A*	-0.475	2.65E-02
TYROBP	1.741	2.07E-02	*THEMIS2*	0.805	2.66E-02
PRDM1	1.467	2.07E-02	*TAGAP*	0.743	2.66E-02
SPIB	1.181	2.07E-02	*CD8A*	0.675	2.66E-02
C11orf75	0.794	2.07E-02	*PLEKHG4*	-0.383	2.66E-02
MARCH9	-0.651	2.07E-02	*SULF1*	0.783	2.67E-02
ITGB2	1.713	2.08E-02	*TAS2R5*	-0.383	2.67E-02
HLA-G	1.428	2.08E-02	*PLAU*	0.758	2.68E-02
AR	-0.565	2.08E-02	*CD226*	0.495	2.68E-02
FAM189A2	-0.601	2.08E-02	*COL4A4*	0.430	2.68E-02
GRHL2	-0.620	2.08E-02	*NCAM2*	0.270	2.68E-02
ENTPD1	1.316	2.09E-02	*TMEM163*	0.147	2.68E-02
CYP1B1	0.939	2.09E-02	*EPS15L1*	-0.415	2.69E-02
CH25H	0.777	2.09E-02	*C15orf61*	-0.477	2.69E-02
DUSP4	-0.539	2.09E-02	*ERI3*	-0.518	2.69E-02
HSD17B8	-0.700	2.09E-02	*SKA3*	-0.547	2.69E-02
VNN2	1.255	2.10E-02	*RAB31*	0.858	2.70E-02
SLA2	1.203	2.10E-02	*PRKCB*	0.700	2.70E-02
IGF2BP3	0.563	2.10E-02	*ANPEP*	0.568	2.70E-02
WFDC2	0.486	2.10E-02	*ABCG5*	-0.450	2.70E-02
NR1I3	-0.549	2.10E-02	*FLRT2*	0.723	2.71E-02
BDNF	-0.554	2.10E-02	*PAG1*	0.593	2.71E-02
C1QTNF3	-0.634	2.10E-02	*IL6R*	0.535	2.71E-02
PLSCR1	1.739	2.11E-02	*KLHL10*	-0.390	2.71E-02
DPP4	0.569	2.11E-02	*SYT4*	-0.430	2.71E-02
CCNA1	0.353	2.11E-02	*EMD*	-0.468	2.71E-02
ZNF621	-0.627	2.11E-02	*NPC2*	0.935	2.72E-02
SELENBP1	-0.640	2.11E-02	*C6orf58*	-0.427	2.72E-02
NFIC	-0.649	2.11E-02	*C5orf64*	-0.440	2.72E-02
LLGL2	-0.653	2.11E-02	*EPHB3*	-0.485	2.72E-02
OASL	1.468	2.12E-02	*PIDD*	-0.550	2.72E-02
CFI	0.972	2.12E-02	*GLIS2*	0.560	2.73E-02
TLR10	0.953	2.12E-02	*KCNIP4*	-0.460	2.73E-02
CLMN	-0.524	2.12E-02	*ATP6V1B2*	0.915	2.74E-02
UGGT2	-0.616	2.12E-02	*PTPRM*	0.818	2.74E-02
ZNF467	-0.722	2.12E-02	*AEBP1*	0.798	2.74E-02
CCRL2	1.115	2.13E-02	*TMC8*	0.763	2.74E-02

PTPLAD2	1.100	2.13E-02	*IL2RB*	0.700	2.74E-02
MUM1L1	-0.530	2.13E-02	*INPP1*	0.590	2.74E-02
ZNF415	-0.583	2.13E-02	*CSGALNACT1*	0.568	2.74E-02
PCBP4	-0.676	2.13E-02	*C2orf74*	0.557	2.74E-02
FBXW9	-0.752	2.13E-02	*ZBTB37*	-0.430	2.74E-02
IFI30	1.696	2.14E-02	*GPRC6A*	-0.483	2.74E-02
LY86	1.563	2.14E-02	*POLQ*	-0.550	2.74E-02
CHN1	0.777	2.14E-02	*CSTA*	0.548	2.75E-02
PRKX	0.609	2.14E-02	*TMEM154*	0.477	2.75E-02
ZCCHC18	0.584	2.14E-02	*ARHGEF5*	-0.383	2.75E-02
BMP2K	1.073	2.15E-02	*TRAF4*	-0.470	2.75E-02
HAPLN3	0.988	2.15E-02	*MED26*	-0.450	2.76E-02
CYP27C1	0.323	2.15E-02	*DDX11*	-0.523	2.77E-02
LIN7A	-0.466	2.15E-02	*ZEB1*	0.878	2.78E-02
SGK2	-0.573	2.15E-02	*IGFBP7*	0.858	2.78E-02
PLEKHG3	-0.603	2.15E-02	*CTHRC1*	0.580	2.78E-02
RAPH1	-0.669	2.15E-02	*GJA1*	0.533	2.78E-02
PTDSS2	-0.699	2.15E-02	*RBKS*	-0.440	2.78E-02
TTC3	-0.778	2.15E-02	*HEPH*	0.535	2.79E-02
ICAM3	1.223	2.16E-02	*SHOX2*	0.363	2.79E-02
APOC2	0.839	2.16E-02	*LINC00467*	-0.430	2.79E-02
NFIX	-0.658	2.16E-02	*NAPEPLD*	-0.500	2.79E-02
SOCS1	0.980	2.17E-02	*UBN2*	-0.517	2.79E-02
TBC1D4	0.656	2.17E-02	*SLIT3*	0.850	2.80E-02
C7orf31	0.610	2.17E-02	*WISP2*	0.758	2.80E-02
SH3BP5L	-0.621	2.17E-02	*CTTNBP2*	0.277	2.80E-02
MUT	-0.758	2.17E-02	*SPECC1*	0.197	2.80E-02
MEOX1	0.437	2.18E-02	*SCGB1D1*	-0.400	2.80E-02
SIGLEC1	1.565	2.19E-02	*SELRC1*	-0.495	2.80E-02
PSME2	1.469	2.19E-02	*NR3C1*	0.965	2.81E-02
NLRP11	-0.464	2.19E-02	*FGFR2*	-0.420	2.81E-02
OR3A2	-0.546	2.19E-02	*ESRRB*	-0.487	2.81E-02
ADCK1	-0.614	2.19E-02	*CELF4*	-0.537	2.81E-02
TNP2	-0.653	2.19E-02	*IL2RG*	0.813	2.82E-02
SNHG8	-0.685	2.19E-02	*KLRB1*	0.693	2.82E-02
DERL3	0.983	2.20E-02	*COX7A1*	0.693	2.82E-02
PDPN	0.883	2.20E-02	*MMP9*	0.585	2.82E-02
TXK	0.648	2.20E-02	*LACE1*	-0.347	2.82E-02
SSBP2	-0.677	2.20E-02	*ZNF35*	-0.390	2.82E-02
TMEM61	-0.682	2.20E-02	*VAMP5*	0.803	2.83E-02
RNF213	1.444	2.21E-02	*PSPH*	-0.448	2.83E-02
C2orf74	0.821	2.21E-02	*SYNPR*	-0.450	2.83E-02
ARHGAP42	0.816	2.21E-02	*FAM64A*	-0.528	2.83E-02
STK11IP	-0.682	2.21E-02	*CD3G*	0.633	2.85E-02
TCEA2	-0.728	2.21E-02	*CCDC43*	-0.500	2.85E-02
HCK	1.694	2.22E-02	*COL1A1*	0.843	2.86E-02
ITGB7	1.338	2.22E-02	*KCNJ8*	0.650	2.86E-02
CD200R1	0.647	2.22E-02	*TDRKH*	-0.400	2.86E-02
CXCL3	0.600	2.22E-02	*BTLA*	0.440	2.87E-02
ZNF862	-0.780	2.22E-02	*HIST1H2AE*	-0.335	2.87E-02
LAIR1	1.622	2.23E-02	*CREB3L4*	-0.473	2.87E-02

DDX60L	1.573	2.23E-02	*KIF23*	-0.543	2.87E-02
DEPDC7	0.439	2.23E-02	*B2M*	0.978	2.88E-02
CBY1	-0.634	2.23E-02	*FRMD4B*	0.568	2.89E-02
GLIS1	-0.700	2.23E-02	*SLC27A5*	-0.510	2.89E-02
CD19	0.998	2.24E-02	*C15orf23*	-0.530	2.89E-02
FMNL3	0.909	2.24E-02	*PCDHGB7*	-0.327	2.90E-02
ACMSD	-0.541	2.24E-02	*NLRP11*	-0.333	2.90E-02
WASL	-0.646	2.24E-02	*HIST1H1D*	-0.405	2.90E-02
ZNF821	-0.718	2.24E-02	*ZFP41*	-0.407	2.90E-02
MDFIC	1.063	2.25E-02	*PP7080*	-0.420	2.90E-02
CD1E	0.764	2.25E-02	*LOC100130557*	-0.470	2.90E-02
PGBD5	0.367	2.25E-02	*GULP1*	0.560	2.91E-02
PDE3A	-0.485	2.25E-02	*FBLN7*	0.373	2.91E-02
SCGB2A2	-0.525	2.25E-02	*NKD2*	0.350	2.91E-02
MRO	-0.583	2.25E-02	*MED20*	-0.428	2.91E-02
GPRC5C	-0.648	2.25E-02	*ZNF707*	-0.470	2.91E-02
PRNT	-0.686	2.25E-02	*EWSR1*	-0.495	2.91E-02
HELB	0.644	2.26E-02	*PCOLCE2*	0.255	2.92E-02
SLC7A6OS	0.510	2.26E-02	*INO80C*	-0.433	2.92E-02
C5orf46	0.509	2.26E-02	*KCNG3*	-0.463	2.92E-02
LYPD6B	-0.491	2.26E-02	*ALDH1A3*	0.423	2.93E-02
WIBG	-0.639	2.26E-02	*NHS*	0.343	2.93E-02
A1BG	-0.659	2.26E-02	*RCAN3*	-0.333	2.93E-02
CELSR3	-0.682	2.26E-02	*ZNF704*	-0.427	2.93E-02
IFI6	1.210	2.27E-02	*LATS1*	-0.438	2.93E-02
VNN1	0.714	2.27E-02	*FAM195A*	-0.540	2.93E-02
PIM1	0.629	2.27E-02	*CMAHP*	0.545	2.94E-02
C1S	1.622	2.28E-02	*PURB*	-0.443	2.94E-02
FGD2	1.609	2.28E-02	*C17orf80*	-0.450	2.94E-02
DENND2D	1.005	2.28E-02	*CCR7*	0.690	2.95E-02
FOXQ1	0.428	2.28E-02	*CX3CR1*	0.538	2.95E-02
UTS2D	0.218	2.28E-02	*GPR18*	0.535	2.95E-02
RIMKLA	-0.696	2.28E-02	*THEMIS*	0.493	2.95E-02
ZC3H12C	1.132	2.29E-02	*PLA2G16*	0.440	2.95E-02
EIF2AK2	1.043	2.29E-02	*RGS9*	-0.388	2.95E-02
ERP44	0.902	2.29E-02	*MT3*	-0.433	2.95E-02
SLC12A2	-0.628	2.29E-02	*ADAM32*	-0.367	2.96E-02
MFNG	1.128	2.30E-02	*MYEOV2*	-0.420	2.96E-02
CCDC19	-0.602	2.30E-02	*NDUFAF5*	-0.463	2.96E-02
ZNF777	-0.623	2.30E-02	*RIMKLA*	-0.493	2.96E-02
ZNF252P-AS1	-0.638	2.30E-02	*TLR1*	0.800	2.97E-02
SBNO1	-0.732	2.30E-02	*EMP1*	0.773	2.97E-02
SLC2A3	1.171	2.31E-02	*P2RY6*	0.603	2.97E-02
RNASE1	0.999	2.31E-02	*BDKRB2*	0.495	2.97E-02
FAR2	0.534	2.31E-02	*IDI2*	-0.370	2.97E-02
C5orf45	-0.686	2.32E-02	*TRMT10A*	-0.487	2.97E-02
KIAA1683	-0.738	2.32E-02	*H1F0*	-0.368	2.98E-02
TNFRSF9	1.244	2.33E-02	*LACTB*	0.877	2.99E-02
CTSH	1.076	2.33E-02	*MX2*	0.698	2.99E-02
ALDH2	0.971	2.33E-02	*TNIK*	0.385	2.99E-02
LGSN	-0.462	2.33E-02	*SLC39A4*	-0.495	2.99E-02

CYB5RL	-0.621	2.33E-02	*MYPOP*	-0.510	2.99E-02
SUOX	-0.645	2.33E-02	*DCST1*	-0.517	2.99E-02
SPRYD3	-0.659	2.33E-02	*SP140*	0.660	3.00E-02
KIAA0195	-0.739	2.33E-02	*LAIR2*	0.323	3.00E-02
ERAP1	1.281	2.34E-02	*C12orf55*	-0.350	3.00E-02
STAR	0.396	2.34E-02	*CCDC169*	-0.437	3.00E-02
MOCOS	0.384	2.34E-02	*YPEL1*	-0.468	3.00E-02
C11orf45	0.247	2.34E-02	*AP1M2*	-0.503	3.00E-02
SYNC	-0.624	2.34E-02	*MAMDC2*	0.417	3.01E-02
KIAA1107	-0.698	2.34E-02	*EGR3*	0.393	3.01E-02
RHBDF1	-0.713	2.34E-02	*COL6A5*	0.213	3.01E-02
SPECC1	0.308	2.35E-02	*IGLL1*	0.205	3.01E-02
ZNF560	0.194	2.35E-02	*TOM1L1*	-0.460	3.01E-02
APOF	-0.499	2.35E-02	*UNC5B*	0.393	3.02E-02
CELSR2	-0.642	2.35E-02	*CLN8*	0.298	3.02E-02
RGS19	1.288	2.36E-02	*HOXB6*	0.130	3.02E-02
RGL4	0.628	2.36E-02	*C18orf21*	-0.470	3.02E-02
ZSWIM2	-0.530	2.37E-02	*FAM136A*	-0.520	3.02E-02
CDHR3	-0.564	2.37E-02	*SMARCC1*	-0.525	3.02E-02
ATP5S	-0.641	2.37E-02	*RCAN2*	0.668	3.03E-02
RASSF2	1.211	2.38E-02	*FMO3*	0.358	3.03E-02
LOC728613	-0.455	2.38E-02	*GSG2*	-0.533	3.03E-02
HOXB5	-0.552	2.38E-02	*KLRG1*	0.540	3.04E-02
CNPY4	-0.588	2.38E-02	*MMEL1*	-0.443	3.04E-02
DSEL	-0.590	2.38E-02	*C20orf132*	-0.453	3.04E-02
MPV17L	-0.704	2.38E-02	*YOD1*	-0.460	3.04E-02
MS4A1	1.160	2.39E-02	*GYPC*	0.653	3.05E-02
OBSL1	-0.666	2.39E-02	*CLIC6*	0.323	3.05E-02
PPOX	-0.699	2.39E-02	*KIAA1383*	-0.360	3.05E-02
LRRK2	1.148	2.40E-02	*OTUD7B*	-0.408	3.05E-02
CR2	0.525	2.40E-02	*SETD4*	-0.418	3.05E-02
KCTD4	-0.511	2.40E-02	*DHX35*	-0.445	3.05E-02
TCP10L	-0.559	2.40E-02	*THEM6*	-0.488	3.05E-02
DFNB31	-0.646	2.40E-02	*MSH5*	-0.510	3.05E-02
DDX60	1.695	2.41E-02	*PMP22*	0.745	3.06E-02
NT5E	0.906	2.41E-02	*CAMK2D*	0.660	3.07E-02
C10orf11	0.627	2.41E-02	*LINC00471*	-0.353	3.07E-02
TM4SF18	0.590	2.41E-02	*TNNT1*	-0.375	3.08E-02
PKNOX1	-0.495	2.41E-02	*TMEM99*	-0.417	3.08E-02
SLC25A21	-0.524	2.41E-02	*HMHA1*	0.793	3.09E-02
RPRML	-0.653	2.41E-02	*IGFBP4*	0.728	3.09E-02
RILPL2	0.943	2.42E-02	*MAGEA4*	0.125	3.09E-02
LINC00476	-0.519	2.42E-02	*SLC46A3*	0.760	3.10E-02
SLC35E2	-0.589	2.42E-02	*COL4A1*	0.755	3.10E-02
NME4	-0.699	2.42E-02	*FYCO1*	0.608	3.10E-02
TMEM140	1.592	2.43E-02	*RASA3*	0.598	3.10E-02
IL4R	1.349	2.43E-02	*APOD*	0.578	3.10E-02
CD47	1.121	2.43E-02	*LTF*	0.343	3.10E-02
ODAM	0.431	2.43E-02	*HEATR4*	-0.357	3.10E-02
MSMB	-0.453	2.43E-02	*DEM1*	-0.380	3.10E-02
FAM20B	-0.651	2.43E-02	*STOM*	0.680	3.11E-02

C16orf53	-0.668	2.43E-02	*CXCL9*	0.645	3.11E-02
NSUN5P2	-0.734	2.43E-02	*BANK1*	0.470	3.11E-02
GPR133	0.464	2.44E-02	*LRRC2*	0.235	3.11E-02
DYDC2	-0.507	2.44E-02	*GOLT1A*	-0.430	3.11E-02
PEBP1	-0.700	2.44E-02	*ZNF621*	-0.437	3.11E-02
SLC7A4	-0.737	2.44E-02	*ATAD5*	-0.533	3.11E-02
REEP2	-0.750	2.44E-02	*RASAL3*	0.810	3.12E-02
ADAM28	0.914	2.45E-02	*MS4A14*	0.487	3.12E-02
TRIM5	0.884	2.45E-02	*MMP12*	0.390	3.12E-02
CTHRC1	0.864	2.45E-02	*TEX12*	-0.313	3.12E-02
DNAJC27	-0.660	2.45E-02	*LGALSL*	-0.373	3.12E-02
SCAMP4	-0.683	2.45E-02	*DCAF4*	-0.390	3.12E-02
C16orf13	-0.764	2.45E-02	*PPP1R3D*	-0.410	3.12E-02
HLA-DOA	1.548	2.46E-02	*APOL3*	0.785	3.13E-02
KLHL11	-0.610	2.46E-02	*CPNE8*	0.560	3.13E-02
IZUMO4	-0.702	2.46E-02	*ETS2*	0.518	3.13E-02
SLC18B1	0.813	2.47E-02	*DCAF17*	-0.518	3.13E-02
SMARCC2	-0.730	2.47E-02	*RPL36*	-0.523	3.13E-02
NR2F6	-0.743	2.47E-02	*PRPF19*	-0.528	3.13E-02
AIF1	1.776	2.48E-02	*DDX60L*	0.813	3.14E-02
TMEM132B	-0.491	2.48E-02	*TNC*	0.655	3.14E-02
RSAD1	-0.669	2.48E-02	*GATM*	0.405	3.14E-02
SH3GL1	-0.708	2.48E-02	*TCF7*	0.358	3.14E-02
TAPBPL	1.124	2.49E-02	*DHX57*	-0.413	3.14E-02
MATN3	-0.523	2.49E-02	*HCST*	0.767	3.15E-02
OBFC1	-0.587	2.49E-02	*PRF1*	0.708	3.15E-02
PAQR6	-0.646	2.49E-02	*ABLIM3*	0.590	3.15E-02
FOXH1	-0.744	2.49E-02	*CD274*	0.567	3.15E-02
POLL	-0.755	2.49E-02	*WDR31*	-0.353	3.15E-02
TTLL3	-0.766	2.49E-02	*CCNB3*	-0.403	3.15E-02
CD300A	1.482	2.50E-02	*TRIM45*	-0.438	3.15E-02
HENMT1	0.505	2.50E-02	*SUGP2*	-0.495	3.15E-02
UBE2QL1	0.387	2.50E-02	*RFC2*	-0.555	3.15E-02
NEURL2	-0.599	2.50E-02	*MAN2A1*	0.670	3.16E-02
BCL9	-0.600	2.50E-02	*TBRG1*	0.627	3.16E-02
SLCO2B1	1.389	2.51E-02	*CR1*	0.618	3.16E-02
CTSL2	0.485	2.51E-02	*IFI27*	0.585	3.16E-02
PCDHB5	-0.467	2.51E-02	*UBD*	0.485	3.16E-02
WDFY4	1.362	2.53E-02	*CLEC5A*	0.455	3.16E-02
LXN	0.851	2.53E-02	*GLDN*	0.287	3.16E-02
INPP4A	0.849	2.53E-02	*CNIH3*	0.235	3.16E-02
PLTP	0.829	2.53E-02	*LCE1E*	-0.450	3.16E-02
TES	0.778	2.53E-02	*TIMM10*	-0.538	3.16E-02
IER5	0.778	2.53E-02	*SYNDIG1*	0.515	3.17E-02
NLGN4Y	-0.485	2.53E-02	*MRPL55*	-0.540	3.17E-02
PCDH9	-0.533	2.53E-02	*PALLD*	0.780	3.18E-02
CKB	-0.561	2.53E-02	*AIM2*	0.620	3.18E-02
CCDC169	-0.601	2.53E-02	*MYO1D*	0.515	3.18E-02
TMC4	-0.685	2.53E-02	*FAAH2*	-0.393	3.18E-02
ZNF598	-0.779	2.53E-02	*HIST1H1A*	-0.468	3.18E-02
LAYN	0.917	2.54E-02	*VIM*	0.885	3.19E-02

VTN	-0.588	2.54E-02	*LATS2*	0.800	3.19E-02
DNASE1L3	0.599	2.55E-02	*HERPUD1*	0.758	3.19E-02
ADTRP	0.382	2.55E-02	*RNF130*	0.675	3.19E-02
ADAMTS17	-0.550	2.55E-02	*CNN2*	0.670	3.19E-02
FRY	-0.660	2.55E-02	*LAMA2*	0.643	3.19E-02
CHST12	0.769	2.56E-02	*ITSN1*	0.600	3.19E-02
IL1R2	0.663	2.56E-02	*LRP2BP*	-0.380	3.19E-02
SPATA17	-0.500	2.56E-02	*CHMP4C*	-0.393	3.19E-02
ATP9B	-0.516	2.56E-02	*APBA2*	-0.398	3.19E-02
KCNJ11	-0.559	2.56E-02	*NXT1*	-0.510	3.19E-02
SUMF2	-0.569	2.56E-02	*DPEP2*	0.740	3.20E-02
CASP4	1.572	2.57E-02	*ITPRIPL2*	0.603	3.20E-02
CCDC109B	1.015	2.57E-02	*CASP8*	0.513	3.20E-02
RTN1	0.537	2.57E-02	*EDNRB*	0.468	3.20E-02
HSF5	0.289	2.57E-02	*TP53RK*	-0.470	3.20E-02
C1orf204	-0.556	2.57E-02	*GOLGA2P5*	-0.473	3.20E-02
SEC14L2	-0.604	2.57E-02	*RABGAP1L*	0.650	3.21E-02
VSIG4	1.221	2.58E-02	*ELMO3*	-0.478	3.21E-02
EPHX3	0.567	2.58E-02	*MACROD1*	-0.508	3.21E-02
HCG27	0.528	2.58E-02	*SNORD114-3*	0.247	3.22E-02
SPR	-0.650	2.58E-02	*NOL4*	-0.403	3.22E-02
FUK	-0.727	2.58E-02	*FN3K*	-0.420	3.22E-02
RASAL3	1.505	2.59E-02	*NCAPD3*	-0.520	3.22E-02
PARVG	1.406	2.59E-02	*PRDM1*	0.750	3.23E-02
SH2D1A	1.229	2.59E-02	*PPM1L*	-0.300	3.23E-02
STK32B	-0.494	2.60E-02	*COX8C*	-0.367	3.23E-02
ANKMY1	-0.558	2.60E-02	*PRDM13*	-0.425	3.23E-02
SARNP	-0.590	2.60E-02	*POU2F1*	-0.453	3.23E-02
MARCKS	1.065	2.61E-02	*HCK*	0.860	3.24E-02
TTC39B	0.506	2.61E-02	*A2M*	0.713	3.24E-02
PHF7	-0.550	2.61E-02	*KLHL29*	0.367	3.24E-02
PVT1	-0.587	2.61E-02	*MSX2*	0.160	3.24E-02
BHLHB9	-0.587	2.61E-02	*IQCH*	-0.400	3.24E-02
ZNF839	-0.650	2.61E-02	*EFNA1*	-0.408	3.24E-02
IFIT5	1.411	2.62E-02	*DDX25*	-0.430	3.24E-02
ETV7	1.270	2.62E-02	*MOB3C*	0.623	3.25E-02
EHD4	1.075	2.62E-02	*BLNK*	0.420	3.25E-02
GPNMB	1.027	2.62E-02	*FCRLA*	0.373	3.25E-02
KRT6B	0.638	2.62E-02	*SMAD9*	-0.423	3.25E-02
RPS4Y1	-0.405	2.62E-02	*ZC3HC1*	-0.490	3.25E-02
EYA1	-0.567	2.62E-02	*KRI1*	-0.530	3.25E-02
REM2	-0.621	2.62E-02	*SP140L*	0.595	3.26E-02
EPS8L2	-0.633	2.62E-02	*RHOH*	0.565	3.26E-02
TBXAS1	1.069	2.63E-02	*LAMP3*	0.475	3.26E-02
ELL3	-0.516	2.63E-02	*FZD3*	-0.440	3.26E-02
HOXC5	-0.535	2.63E-02	*RAB33A*	0.325	3.27E-02
NLRC4	0.982	2.64E-02	*LOC654433*	0.120	3.27E-02
SLC25A53	0.308	2.64E-02	*SNORD8*	-0.367	3.27E-02
ADAM3A	-0.535	2.64E-02	*NELL1*	-0.448	3.27E-02
SLC25A41	-0.590	2.64E-02	*CLPB*	-0.485	3.27E-02
SLC9A1	-0.646	2.64E-02	*EPRS*	-0.495	3.27E-02

PPP1R13L	-0.684	2.64E-02	*SRRT*	-0.543	3.27E-02
SYTL1	-0.693	2.64E-02	*TPP1*	1.010	3.28E-02
SERPING1	1.362	2.65E-02	*SLFN12*	0.575	3.28E-02
ELF4	1.234	2.65E-02	*DFNA5*	0.555	3.28E-02
FNBP1	1.221	2.65E-02	*AQP11*	-0.367	3.28E-02
CHI3L1	0.602	2.65E-02	*LINC00339*	-0.385	3.28E-02
RNF150	0.579	2.65E-02	*MAPKAPK5-AS1*	-0.450	3.28E-02
CDKN2A	0.457	2.65E-02	*PPP1R35*	-0.473	3.28E-02
HOXD4	-0.524	2.65E-02	*ASPN*	0.500	3.29E-02
ARAP3	-0.586	2.65E-02	*ZIC1*	-0.323	3.29E-02
DOK6	-0.600	2.65E-02	*E2F7*	-0.493	3.29E-02
GBA2	-0.623	2.65E-02	*ZNF783*	-0.505	3.29E-02
PDDC1	-0.681	2.65E-02	*FMNL2*	0.533	3.30E-02
KRBA1	-0.754	2.65E-02	*TAF1B*	-0.478	3.30E-02
ZNF446	-0.789	2.65E-02	*VEPH1*	-0.347	3.31E-02
STEAP1	0.574	2.66E-02	*ASB13*	-0.403	3.31E-02
IL7	0.526	2.66E-02	*CCDC77*	-0.487	3.31E-02
PPME1	-0.579	2.66E-02	*CXCL11*	0.438	3.32E-02
MACROD1	-0.708	2.66E-02	*C6orf164*	-0.377	3.32E-02
MYO1F	1.661	2.67E-02	*SPAG5*	-0.533	3.32E-02
DOCK11	1.472	2.67E-02	*STAB1*	0.900	3.33E-02
NFKB1	1.183	2.67E-02	*WDFY1*	0.750	3.33E-02
ASB2	0.586	2.67E-02	*CST7*	0.690	3.33E-02
HIST1H2AB	-0.542	2.67E-02	*EGR1*	0.420	3.33E-02
ASB1	-0.559	2.67E-02	*TNFRSF11A*	0.228	3.33E-02
ARL3	-0.565	2.67E-02	*C7orf29*	0.167	3.33E-02
ZMYND8	-0.635	2.67E-02	*SLC25A21*	-0.373	3.33E-02
COTL1	1.319	2.68E-02	*C11orf84*	-0.467	3.33E-02
CXCL16	1.156	2.68E-02	*WDR74*	-0.538	3.33E-02
EMR1	0.976	2.68E-02	*GLUL*	0.443	3.34E-02
MCTP1	0.828	2.68E-02	*GCNT3*	-0.398	3.34E-02
REC8	0.683	2.68E-02	*BHLHE22*	0.560	3.35E-02
ARHGAP22	0.607	2.68E-02	*CCDC102B*	0.375	3.35E-02
JAKMIP1	0.387	2.68E-02	*P2RX5*	0.358	3.35E-02
CERS5	-0.571	2.69E-02	*ZNF418*	-0.433	3.35E-02
KIRREL2	-0.693	2.69E-02	*METTL13*	-0.505	3.35E-02
CXCL6	0.443	2.70E-02	*SNRNP25*	-0.545	3.35E-02
JAKMIP2	0.381	2.70E-02	*TNS3*	0.673	3.36E-02
LOC283481	-0.543	2.70E-02	*UBASH3A*	0.548	3.36E-02
APBB2	-0.584	2.70E-02	*ACTR5*	-0.455	3.36E-02
SLC41A2	0.982	2.71E-02	*CCDC114*	-0.483	3.36E-02
GREM1	0.673	2.71E-02	*OPN5*	-0.563	3.36E-02
TOX	0.423	2.71E-02	*SERPINE1*	0.805	3.37E-02
FLNB	-0.578	2.71E-02	*CD1C*	0.633	3.37E-02
PPDPF	-0.684	2.71E-02	*MAGEA12*	0.145	3.37E-02
RNF166	0.886	2.72E-02	*FAM228A*	-0.373	3.37E-02
UMODL1	-0.463	2.72E-02	*MARCH4*	-0.450	3.37E-02
LRRC46	-0.575	2.72E-02	*EPN3*	-0.463	3.37E-02
ASIC3	-0.609	2.72E-02	*ARHGAP11A*	-0.515	3.37E-02
SLC2A11	-0.627	2.72E-02	*PLAT*	0.553	3.38E-02

C5orf56	0.725	2.73E-02	*C7*	0.545	3.38E-02
MAML3	-0.509	2.73E-02	*GABRA5*	-0.468	3.38E-02
DLG3	-0.560	2.73E-02	*MAGI1*	-0.475	3.38E-02
FAM100A	-0.713	2.73E-02	*COL14A1*	0.740	3.39E-02
TBC1D10C	1.377	2.74E-02	*LOC375295*	0.260	3.39E-02
TMEM154	0.617	2.74E-02	*XRRA1*	-0.293	3.39E-02
TNFRSF10A	0.577	2.74E-02	*FAM184B*	-0.370	3.39E-02
TNFRSF11B	0.458	2.74E-02	*ZFYVE9*	-0.410	3.39E-02
KIAA1875	-0.691	2.74E-02	*RPRD1A*	-0.548	3.39E-02
BEND4	0.268	2.75E-02	*FBLN1*	0.898	3.40E-02
RGS7BP	-0.453	2.75E-02	*GIT2*	0.730	3.40E-02
ARSK	-0.555	2.75E-02	*LAT*	0.650	3.40E-02
CNTN1	-0.564	2.75E-02	*ADAMTSL4*	0.470	3.40E-02
CST9	-0.597	2.75E-02	*GIPC2*	0.255	3.40E-02
ZNF706	-0.599	2.75E-02	*ZNF8*	-0.400	3.40E-02
PELI3	-0.624	2.75E-02	*MGAT1*	0.920	3.41E-02
PLA2G4C	0.770	2.76E-02	*UBE2QL1*	0.240	3.41E-02
SGSM1	-0.759	2.76E-02	*CXorf69*	0.213	3.41E-02
ADAP2	1.239	2.77E-02	*GARNL3*	-0.423	3.41E-02
GNA15	0.963	2.77E-02	*TUBB2A*	0.500	3.42E-02
POLR2J4	-0.551	2.77E-02	*INPP4B*	0.355	3.42E-02
ORM1	0.222	2.78E-02	*ZSCAN22*	-0.357	3.42E-02
TSPAN13	-0.561	2.78E-02	*GPX8*	0.703	3.43E-02
PSME1	1.470	2.79E-02	*SPATS2L*	0.600	3.43E-02
TCP11L1	0.629	2.79E-02	*SLCO2A1*	0.580	3.43E-02
CYP27B1	0.526	2.79E-02	*TMTC1*	0.447	3.43E-02
FKBP5	0.522	2.79E-02	*EDEM1*	0.620	3.44E-02
SECTM1	1.129	2.80E-02	*PTGFRN*	0.440	3.44E-02
GFPT2	0.963	2.80E-02	*EFCAB4B*	-0.387	3.44E-02
SNX18	0.426	2.80E-02	*DPH3P1*	-0.443	3.44E-02
RGS10	0.737	2.81E-02	*BMP2K*	0.620	3.45E-02
AFMID	-0.552	2.81E-02	*FBP1*	0.438	3.45E-02
ATP6AP1L	-0.607	2.81E-02	*FDCSP*	0.207	3.45E-02
C12orf76	-0.614	2.81E-02	*SKAP2*	0.805	3.46E-02
DLX2	-0.616	2.81E-02	*CARD8*	0.738	3.46E-02
PSMB10	1.614	2.82E-02	*RBBP4*	-0.487	3.46E-02
MAP4K1	1.481	2.82E-02	*RAB42*	0.173	3.47E-02
TMEM170A	0.799	2.82E-02	*TUSC1*	-0.373	3.47E-02
LOC100169752	-0.452	2.82E-02	*CD2*	0.718	3.48E-02
CCDC158	-0.546	2.82E-02	*SH3D19*	0.653	3.48E-02
SLC15A3	1.595	2.83E-02	*PDE2A*	0.593	3.48E-02
PIP4K2C	-0.519	2.83E-02	*DIS3L2*	-0.390	3.48E-02
PCLO	-0.646	2.83E-02	*ZNF142*	-0.468	3.48E-02
JAZF1	1.045	2.84E-02	*LOC202781*	-0.617	3.48E-02
COL6A5	0.305	2.84E-02	*UGCG*	0.610	3.49E-02
MOB3C	0.955	2.85E-02	*SLAMF1*	0.553	3.49E-02
CUEDC1	-0.599	2.85E-02	*PALMD*	0.473	3.49E-02
INPP5D	1.248	2.86E-02	*ANTXR1*	0.698	3.50E-02
FBXO6	1.002	2.86E-02	*RGS1*	0.493	3.50E-02
KLRG1	0.757	2.86E-02	*PIM2*	0.450	3.50E-02
FNDC5	-0.662	2.86E-02	*SPATA5*	-0.480	3.50E-02

C20orf79	-0.664	2.86E-02	*MIR100HG*	0.620	3.51E-02
RORC	-0.676	2.87E-02	*NLRC4*	0.583	3.51E-02
GAS2L1	-0.678	2.87E-02	*CD38*	0.443	3.51E-02
ZNF10	-0.686	2.87E-02	*DOK7*	-0.387	3.51E-02
APOBEC3B	0.754	2.88E-02	*C10orf122*	-0.413	3.51E-02
ATP10A	0.485	2.88E-02	*PPM1J*	-0.423	3.51E-02
ENOSF1	-0.599	2.88E-02	*KRTAP7-1*	-0.440	3.51E-02
FAM211B	-0.680	2.88E-02	*FAM184A*	-0.453	3.51E-02
OPTN	1.564	2.89E-02	*POLA2*	-0.530	3.51E-02
CLDN1	0.573	2.89E-02	*FBLN5*	0.665	3.52E-02
ARHGAP5-AS1	-0.520	2.89E-02	*PHLDB2*	0.620	3.52E-02
FGGY	-0.536	2.89E-02	*CPED1*	0.583	3.52E-02
SLC35B2	-0.654	2.89E-02	*SULF2*	0.500	3.52E-02
ZC3HAV1	0.837	2.90E-02	*GALNT10*	0.465	3.52E-02
STON2	-0.498	2.90E-02	*KIAA1257*	-0.380	3.52E-02
ZFYVE9	-0.574	2.90E-02	*FAAH*	-0.483	3.52E-02
KIAA1407	-0.694	2.90E-02	*ISM1*	0.263	3.53E-02
DHX32	-0.495	2.91E-02	*SGPP2*	0.210	3.53E-02
GSTA4	-0.609	2.91E-02	*DZANK1*	-0.425	3.53E-02
MTX3	-0.658	2.91E-02	*SPPL2A*	0.853	3.54E-02
ARHGAP17	0.785	2.93E-02	*CD302*	0.690	3.54E-02
DYRK2	0.696	2.93E-02	*CLEC10A*	0.638	3.54E-02
FERMT1	0.565	2.93E-02	*FAM49A*	0.550	3.54E-02
SAMD12	-0.423	2.93E-02	*FAM89A*	0.193	3.54E-02
SCUBE2	-0.563	2.93E-02	*MKNK1*	0.615	3.55E-02
HECTD2	-0.685	2.93E-02	*RSBN1L*	-0.460	3.55E-02
FLI1	1.312	2.94E-02	*GBP2*	0.853	3.56E-02
SCARF1	0.652	2.94E-02	*IFI30*	0.840	3.56E-02
ZNF233	-0.499	2.94E-02	*ACTC1*	0.388	3.56E-02
E4F1	-0.749	2.94E-02	*HAR1A*	0.280	3.56E-02
SPPL2B	-0.758	2.94E-02	*PITPNC1*	0.248	3.56E-02
CORO1A	1.410	2.95E-02	*LINC00515*	-0.337	3.56E-02
OLFM4	0.166	2.96E-02	*RAB8B*	0.938	3.57E-02
HTT	-0.660	2.96E-02	*MYOF*	0.808	3.57E-02
CFLAR	1.362	2.97E-02	*SERPINB1*	0.743	3.57E-02
HLA-J	1.340	2.97E-02	*WLS*	0.518	3.57E-02
EBI3	1.051	2.97E-02	*B3GNT9*	0.503	3.57E-02
C8orf4	0.495	2.97E-02	*DTX4*	0.268	3.57E-02
RGS7	-0.518	2.97E-02	*FBXL21*	-0.387	3.57E-02
PCSK6	-0.593	2.97E-02	*CBLC*	-0.458	3.57E-02
MYH10	-0.619	2.97E-02	*COL5A1*	0.855	3.58E-02
SLC26A10	-0.660	2.97E-02	*FAIM3*	0.600	3.58E-02
ANKRD19P	-0.472	2.98E-02	*MAGEF1*	-0.440	3.58E-02
UBXN10	-0.522	2.98E-02	*PLCG2*	0.540	3.59E-02
DNAL1	-0.558	2.98E-02	*CYP1B1-AS1*	0.140	3.59E-02
NEK11	-0.612	2.98E-02	*POU2F3*	-0.368	3.59E-02
KHDRBS2	-0.699	2.98E-02	*ZNF568*	-0.400	3.59E-02
C1R	1.416	2.99E-02	*SPATA6*	-0.400	3.59E-02
TGM2	1.120	2.99E-02	*CDCA4*	-0.510	3.59E-02
ZNF438	0.712	2.99E-02	*MRTO4*	-0.535	3.59E-02
CARD9	0.626	2.99E-02	*HSD11B1L*	-0.550	3.59E-02

TXLNB	0.313	2.99E-02	*ANXA1*	0.808	3.60E-02
HN1L	-0.653	2.99E-02	*LOC388692*	0.410	3.60E-02
AAED1	0.974	3.00E-02	*LOH12CR2*	-0.400	3.60E-02
CXCL2	0.821	3.00E-02	*EXOSC5*	-0.538	3.60E-02
H2AFY2	0.413	3.01E-02	*CIAO1*	-0.468	3.61E-02
NUDCD3	-0.605	3.01E-02	*TIMP2*	0.903	3.62E-02
MAP3K5	0.996	3.02E-02	*CD27*	0.645	3.62E-02
UNC13C	-0.496	3.02E-02	*MMP7*	0.258	3.62E-02
KCTD12	1.144	3.03E-02	*SLC2A1*	-0.365	3.62E-02
ATF5	0.712	3.03E-02	*NPHP1*	-0.367	3.62E-02
DDIT4	0.510	3.03E-02	*BPHL*	-0.410	3.62E-02
NUB1	1.018	3.04E-02	*B9D1*	-0.468	3.62E-02
NBAS	-0.588	3.04E-02	*ELL2*	0.650	3.63E-02
IL17B	-0.600	3.04E-02	*PDZRN4*	0.305	3.63E-02
LRRC33	0.772	3.05E-02	*GTF3C2*	-0.485	3.63E-02
ITM2C	0.678	3.05E-02	*F3*	0.533	3.64E-02
DDX25	-0.588	3.05E-02	*F2RL2*	0.453	3.64E-02
LACTB	1.502	3.06E-02	*TMEM186*	-0.468	3.64E-02
VAMP1	0.587	3.06E-02	*ZBTB8B*	-0.497	3.64E-02
PITPNC1	0.369	3.06E-02	*MRPL43*	-0.507	3.64E-02
RNF175	0.248	3.06E-02	*SLC31A2*	0.780	3.65E-02
ING5	-0.620	3.06E-02	*TFPI*	0.603	3.65E-02
ABCC12	-0.649	3.06E-02	*STS*	0.408	3.65E-02
PDK2	-0.701	3.06E-02	*MSRA*	0.405	3.65E-02
LY96	1.635	3.07E-02	*AIFM3*	-0.427	3.65E-02
PPP1R18	1.617	3.07E-02	*C6orf123*	-0.473	3.65E-02
CSF1R	1.450	3.07E-02	*SLAMF7*	0.655	3.66E-02
PABPC5	-0.526	3.07E-02	*EIF4E3*	0.593	3.66E-02
C1orf213	-0.649	3.07E-02	*SCARA5*	0.510	3.66E-02
CAPN6	0.737	3.08E-02	*P2RY10*	0.503	3.66E-02
USP27X	-0.475	3.08E-02	*SPRYD7*	-0.400	3.66E-02
BSPRY	-0.571	3.08E-02	*APTX*	-0.483	3.66E-02
ALDH4A1	-0.623	3.08E-02	*DUSP3*	0.645	3.67E-02
SCRN2	-0.710	3.08E-02	*KCNJ2*	0.408	3.67E-02
C2orf15	-0.528	3.09E-02	*HOXA7*	0.223	3.67E-02
CROT	-0.588	3.09E-02	*LSM11*	-0.350	3.67E-02
IFT81	-0.677	3.09E-02	*DHPS*	-0.513	3.67E-02
ALKBH7	-0.735	3.09E-02	*PARP14*	0.823	3.68E-02
LGALS8-AS1	-0.760	3.09E-02	*COL12A1*	0.697	3.68E-02
PTAFR	1.507	3.10E-02	*CTLA4*	0.600	3.68E-02
PLEKHS1	0.125	3.10E-02	*ZBTB16*	0.345	3.68E-02
BCL2L14	0.996	3.11E-02	*CACNA2D3*	0.145	3.68E-02
CASP7	0.955	3.11E-02	*ZDHHC13*	-0.465	3.68E-02
S1PR1	0.895	3.11E-02	*ZNF837*	-0.517	3.69E-02
ECI1	-0.707	3.11E-02	*COQ3*	-0.518	3.69E-02
SNX20	0.871	3.12E-02	*OMD*	0.575	3.70E-02
BATF3	0.766	3.12E-02	*SGIP1*	0.483	3.70E-02
FLJ34503	-0.499	3.12E-02	*FAM107B*	0.450	3.70E-02
C1orf168	-0.511	3.12E-02	*EAF2*	0.440	3.70E-02
VAPB	-0.513	3.12E-02	*HBB*	0.318	3.70E-02
SRGAP3	-0.603	3.12E-02	*OLFM4*	0.080	3.70E-02

SLC25A17	-0.624	3.12E-02	*SLCO6A1*	-0.390	3.70E-02
PMM1	-0.659	3.12E-02	*CASP1*	0.813	3.71E-02
PANK2	0.785	3.13E-02	*SPRED1*	0.740	3.71E-02
SFRP2	0.627	3.13E-02	*BACH2*	0.448	3.71E-02
PBX4	0.475	3.13E-02	*SP100*	0.738	3.72E-02
LINC00471	-0.472	3.13E-02	*PVRIG*	0.585	3.72E-02
MTHFSD	-0.528	3.13E-02	*HGF*	0.488	3.72E-02
C17orf105	-0.541	3.13E-02	*SCN5A*	-0.423	3.72E-02
SERPINA10	-0.621	3.13E-02	*COPS7B*	-0.458	3.72E-02
RSG1	-0.632	3.13E-02	*CELSR3*	-0.470	3.72E-02
ABCB6	-0.535	3.14E-02	*RILPL2*	0.567	3.73E-02
TNRC6C	-0.594	3.14E-02	*CORO2B*	0.488	3.73E-02
COLQ	-0.632	3.14E-02	*RNF125*	0.330	3.73E-02
NFKBIA	1.014	3.15E-02	*XK*	-0.375	3.73E-02
FAM122C	0.549	3.15E-02	*MUTYH*	-0.488	3.73E-02
PCED1A	-0.666	3.15E-02	*NCDN*	-0.493	3.73E-02
CD97	1.256	3.16E-02	*AKNA*	0.630	3.74E-02
TDRD7	1.179	3.16E-02	*ATP8B4*	0.538	3.74E-02
PILRA	1.109	3.16E-02	*EPHA4*	0.375	3.74E-02
FBXL13	0.260	3.16E-02	*AFAP1L1*	0.330	3.74E-02
ZNF250	-0.525	3.16E-02	*LRP6*	-0.468	3.74E-02
ANKRD54	-0.625	3.16E-02	*OGFOD2*	-0.523	3.74E-02
TRAFD1	0.848	3.17E-02	*NME1*	-0.563	3.74E-02
RCN1	0.729	3.17E-02	*C3orf79*	-0.327	3.75E-02
ID3	0.463	3.17E-02	*ECM1*	0.635	3.76E-02
RHBDL2	0.449	3.17E-02	*PARP6*	-0.450	3.76E-02
GLI3	-0.605	3.17E-02	*MIR22HG*	0.758	3.77E-02
PLEKHO1	1.152	3.18E-02	*PDGFC*	0.695	3.77E-02
PDCD1LG2	1.073	3.18E-02	*HCP5*	0.533	3.77E-02
GFOD1	0.276	3.18E-02	*EREG*	0.273	3.77E-02
BCAS1	-0.533	3.18E-02	*ST6GALNAC1*	0.133	3.77E-02
STXBP4	-0.569	3.18E-02	*ST7L*	-0.375	3.77E-02
LOC728537	-0.581	3.18E-02	*APOBEC3G*	0.633	3.78E-02
IGSF3	-0.515	3.19E-02	*LPIN2*	0.533	3.78E-02
FLRT3	-0.516	3.19E-02	*GHR*	0.483	3.78E-02
ZNF193	-0.526	3.19E-02	*TMEM27*	-0.357	3.78E-02
NKX3-2	-0.587	3.19E-02	*TNS1*	0.813	3.79E-02
MKL2	-0.659	3.19E-02	*SERPINA1*	0.633	3.79E-02
VAMP5	1.226	3.20E-02	*SCN4B*	0.387	3.79E-02
KMO	0.665	3.20E-02	*C3orf33*	-0.393	3.79E-02
HAS2	0.587	3.20E-02	*MAPK8*	-0.445	3.79E-02
FNDC1	0.593	3.21E-02	*NBL1*	0.595	3.80E-02
KRT222	-0.472	3.21E-02	*ZNF786*	-0.420	3.80E-02
RAB3IP	-0.571	3.21E-02	*CEP70*	-0.470	3.80E-02
ARID2	-0.751	3.21E-02	*ITGA2B*	-0.503	3.80E-02
LST1	1.647	3.22E-02	*EDNRA*	0.615	3.81E-02
TMEM30B	-0.561	3.22E-02	*TBC1D2*	0.590	3.81E-02
ZNF263	-0.596	3.22E-02	*RAB37*	0.410	3.81E-02
OR2W1	-0.596	3.22E-02	*PRKRIP1*	-0.440	3.81E-02
LINC00612	-0.663	3.22E-02	*LRAT*	-0.333	3.82E-02
NECAP2	1.448	3.23E-02	*RNF41*	-0.400	3.82E-02

C9orf117	-0.653	3.23E-02	*CTU2*	-0.520	3.82E-02
SVOPL	0.188	3.24E-02	*TRPV2*	0.683	3.83E-02
PIGM	-0.619	3.24E-02	*AGTR1*	0.525	3.83E-02
JAK2	1.523	3.25E-02	*KIAA1199*	0.398	3.83E-02
CSRP2	0.429	3.25E-02	*STEAP2*	0.360	3.83E-02
TTLL11	-0.509	3.25E-02	*PMCHL1*	-0.357	3.83E-02
EXOC6	-0.615	3.25E-02	*ALCAM*	0.473	3.84E-02
PLXNC1	0.835	3.26E-02	*ATXN1L*	0.467	3.84E-02
NME8	0.524	3.26E-02	*CNTN3*	0.327	3.84E-02
XPNPEP3	-0.482	3.26E-02	*KCTD6*	-0.410	3.84E-02
CABS1	-0.516	3.26E-02	*EME1*	-0.487	3.84E-02
LILRA4	0.976	3.27E-02	*PIGQ*	-0.523	3.84E-02
C12orf66	-0.608	3.27E-02	*POLR2F*	-0.533	3.84E-02
ITFG3	-0.691	3.27E-02	*TRIM22*	0.903	3.85E-02
ZMYND10	-0.719	3.27E-02	*TPK1*	0.553	3.85E-02
PTPRE	1.211	3.28E-02	*CXorf65*	0.427	3.85E-02
ARHGDIB	1.179	3.28E-02	*KAL1*	0.355	3.85E-02
FYN	0.977	3.28E-02	*TMEM30B*	-0.403	3.85E-02
SIK1	0.612	3.28E-02	*MAGEH1*	0.558	3.86E-02
RGCC	0.511	3.28E-02	*UGT2B28*	0.155	3.86E-02
APLF	0.438	3.28E-02	*FAM201A*	-0.343	3.86E-02
IRF2	1.230	3.29E-02	*MYO10*	-0.368	3.86E-02
DIS3L2	-0.529	3.29E-02	*ZNF285*	-0.390	3.86E-02
SLC5A8	-0.649	3.29E-02	*C17orf101*	-0.433	3.86E-02
DHRS9	0.642	3.30E-02	*JMJD4*	-0.500	3.86E-02
HAMP	0.629	3.30E-02	*NDUFA7*	-0.528	3.86E-02
SHROOM3	-0.497	3.30E-02	*TPM1*	0.590	3.87E-02
FASLG	1.182	3.31E-02	*FAM133A*	0.187	3.87E-02
CHI3L2	0.449	3.31E-02	*ANKRD45*	-0.310	3.87E-02
ARL11	0.359	3.31E-02	*POLR1A*	-0.443	3.87E-02
MT1F	0.347	3.31E-02	*EPT1*	-0.517	3.87E-02
C11orf95	-0.586	3.31E-02	*PRC1*	-0.533	3.87E-02
CD14	1.376	3.32E-02	*APLNR*	0.738	3.88E-02
SERPINB1	1.217	3.32E-02	*RSAD2*	0.538	3.88E-02
RHEBL1	0.448	3.32E-02	*THSD7A*	0.415	3.88E-02
SLC14A1	-0.456	3.32E-02	*PRG4*	0.275	3.88E-02
CNGA4	-0.531	3.32E-02	*LOC100129726*	-0.483	3.88E-02
DAND5	-0.628	3.32E-02	*PTTG1*	-0.523	3.88E-02
DDAH2	-0.630	3.32E-02	*IGFLR1*	0.578	3.89E-02
ARSB	0.794	3.34E-02	*LHFPL1*	-0.353	3.89E-02
ZNF280B	0.569	3.34E-02	*LOC647946*	-0.357	3.89E-02
ANKRD52	-0.576	3.34E-02	*A1BG*	-0.453	3.89E-02
ADAMTS1	0.681	3.35E-02	*CCDC167*	-0.477	3.89E-02
RNF113A	0.665	3.35E-02	*ACSL1*	0.633	3.90E-02
FLT1	0.492	3.35E-02	*IRAK3*	0.585	3.90E-02
LOC158572	-0.509	3.35E-02	*CLDN11*	0.475	3.90E-02
LINC00085	-0.584	3.35E-02	*CCBE1*	0.373	3.90E-02
CHRM4	-0.605	3.35E-02	*NOG*	0.103	3.90E-02
GLRX	1.063	3.36E-02	*NLGN4Y*	-0.338	3.90E-02
DEPTOR	-0.523	3.36E-02	*WBP1L*	0.795	3.91E-02
FAM170A	-0.611	3.36E-02	*ITPR1*	0.543	3.91E-02

TMEM121	-0.640	3.36E-02	*CLIC5*	0.450	3.91E-02
EIF2B4	-0.687	3.36E-02	*MGC57346*	-0.370	3.91E-02
GPX1	0.778	3.37E-02	*NMNAT1*	-0.390	3.91E-02
UBE2E2	0.639	3.37E-02	*GOLGA7B*	-0.527	3.91E-02
LOC255411	-0.562	3.37E-02	*ORC1*	-0.533	3.91E-02
TMEM80	-0.610	3.37E-02	*CTSO*	0.920	3.92E-02
ATP5SL	-0.655	3.37E-02	*PTPRB*	0.633	3.92E-02
OTUD7A	-0.660	3.37E-02	*HTRA3*	0.613	3.92E-02
FGF12	-0.514	3.38E-02	*HLX*	0.585	3.92E-02
PGAP3	-0.656	3.38E-02	*HS3ST3A1*	0.275	3.92E-02
ATP6V1B2	1.490	3.39E-02	*S100A7*	0.160	3.92E-02
N4BP1	0.975	3.39E-02	*DCUN1D2*	-0.410	3.92E-02
METTL21A	0.633	3.39E-02	*FKBP15*	0.695	3.93E-02
TNFRSF21	0.503	3.39E-02	*TPM4*	0.633	3.93E-02
DTX4	0.366	3.39E-02	*ALPK2*	0.357	3.93E-02
C1orf87	-0.571	3.39E-02	*ATP10A*	0.318	3.93E-02
FLVCR1-AS1	-0.473	3.40E-02	*GPR174*	0.260	3.93E-02
OLFM2	-0.561	3.40E-02	*MUC15*	0.167	3.93E-02
TAF6	-0.619	3.40E-02	*BFSP1*	-0.333	3.93E-02
ZNF837	-0.717	3.41E-02	*ITLN1*	-0.367	3.93E-02
HMHA1	1.210	3.42E-02	*GOT1*	-0.448	3.93E-02
CX3CL1	0.825	3.42E-02	*ISLR*	0.690	3.94E-02
LOC339803	0.422	3.42E-02	*TMEM176A*	0.550	3.94E-02
ZC3H3	-0.649	3.42E-02	*FAM5C*	0.093	3.94E-02
MFSD10	-0.695	3.42E-02	*GZMB*	0.500	3.95E-02
EML4	0.954	3.43E-02	*C14orf182*	-0.350	3.95E-02
ABHD14B	-0.697	3.43E-02	*REXO1*	-0.510	3.95E-02
WDFY1	1.159	3.44E-02	*LRRC15*	0.608	3.96E-02
RCAN3	-0.438	3.44E-02	*PLCXD2*	0.233	3.96E-02
ANO1	-0.444	3.44E-02	*RP9P*	-0.337	3.96E-02
H2AFJ	-0.512	3.44E-02	*RASEF*	-0.347	3.96E-02
RAB32	0.864	3.45E-02	*PPME1*	-0.405	3.96E-02
TMC5	-0.503	3.45E-02	*ZNF137P*	-0.463	3.96E-02
ZMAT1	-0.697	3.45E-02	*CHAF1B*	-0.508	3.96E-02
DSE	1.496	3.46E-02	*DPY30*	-0.527	3.96E-02
MSR1	0.949	3.46E-02	*PIK3R5*	0.790	3.97E-02
CCDC96	-0.530	3.46E-02	*NID1*	0.653	3.97E-02
FAM173A	-0.745	3.46E-02	*MCAM*	0.613	3.97E-02
DUSP10	0.576	3.47E-02	*STAP1*	0.370	3.97E-02
LRRC8D	0.521	3.47E-02	*PNMA2*	0.318	3.97E-02
PTCHD1	0.391	3.47E-02	*ZC3HAV1L*	-0.330	3.97E-02
SDR16C5	-0.470	3.47E-02	*CHKA*	-0.380	3.97E-02
NXNL2	-0.473	3.47E-02	*C1orf213*	-0.457	3.97E-02
OSBPL1A	-0.567	3.47E-02	*BATF2*	0.483	3.98E-02
SERPINF1	0.766	3.48E-02	*SF3A2*	-0.513	3.98E-02
GCNT2	0.350	3.48E-02	*GEM*	0.618	3.99E-02
LYSMD4	0.339	3.48E-02	*FAM13C*	0.410	3.99E-02
SIT1	1.292	3.49E-02	*CYS1*	0.407	3.99E-02
KCNJ2	0.541	3.49E-02	*NGFR*	0.398	3.99E-02
SH2D1B	0.336	3.49E-02	*OLFM2*	-0.403	3.99E-02
XKRX	-0.458	3.49E-02	*CAPS*	-0.530	3.99E-02

ATP1B2	-0.698	3.49E-02	*PARVG*	0.703	4.00E-02
CD4	1.362	3.50E-02	*TNFAIP8L2*	0.660	4.00E-02
RNF19B	1.063	3.50E-02	*SCIN*	0.170	4.00E-02
KIF21B	0.696	3.50E-02	*SLC17A8*	-0.357	4.00E-02
DISC1	0.484	3.50E-02	*ENY2*	-0.483	4.00E-02
LGALS3BP	1.060	3.51E-02	*ANXA11*	0.695	4.01E-02
GM2A	0.966	3.51E-02	*PARD6G*	-0.370	4.01E-02
GYPC	0.878	3.51E-02	*ABCB6*	-0.383	4.01E-02
COX8C	-0.484	3.52E-02	*DIRAS1*	-0.460	4.01E-02
ZC3H10	-0.498	3.52E-02	*SP110*	0.738	4.02E-02
MCF2L	-0.621	3.52E-02	*ADH1C*	0.293	4.02E-02
CYP7B1	0.415	3.53E-02	*SHISA2*	0.123	4.02E-02
DACH1	-0.473	3.53E-02	*DECR2*	-0.440	4.02E-02
H1F0	-0.479	3.53E-02	*FOS*	0.438	4.03E-02
DCAKD	-0.519	3.53E-02	*ANKRD22*	0.427	4.03E-02
SV2A	-0.551	3.53E-02	*C19orf12*	0.303	4.03E-02
C16orf74	-0.580	3.53E-02	*APCDD1*	0.303	4.03E-02
C1orf172	-0.634	3.53E-02	*SPATS1*	-0.420	4.03E-02
FGFR4	-0.668	3.53E-02	*PTDSS2*	-0.478	4.03E-02
TRIM3	-0.711	3.53E-02	*KDM1A*	-0.490	4.03E-02
YBX1	0.943	3.54E-02	*OBSCN*	-0.517	4.03E-02
PNPLA4	-0.546	3.54E-02	*RPS6KA2*	0.613	4.04E-02
NRTN	-0.569	3.54E-02	*NINJ2*	0.303	4.04E-02
CCDC159	-0.658	3.54E-02	*CUTA*	-0.503	4.05E-02
HSD17B3	-0.513	3.55E-02	*SIGLEC10*	0.590	4.06E-02
FOXD2	-0.554	3.55E-02	*AGPAT4*	0.373	4.06E-02
C1orf53	-0.603	3.55E-02	*FAM71E1*	-0.467	4.06E-02
CLEC4E	0.887	3.56E-02	*XKRX*	-0.333	4.07E-02
RHOF	0.745	3.56E-02	*CNNM4*	-0.348	4.07E-02
CCDC89	0.194	3.56E-02	*LOC152217*	-0.487	4.07E-02
C3orf79	-0.443	3.56E-02	*FERMT2*	0.730	4.08E-02
LINC00339	-0.508	3.56E-02	*WDFY4*	0.673	4.08E-02
FCN1	1.104	3.57E-02	*RASSF2*	0.625	4.08E-02
DEF6	1.038	3.57E-02	*SLC40A1*	0.623	4.08E-02
KIAA0922	0.561	3.57E-02	*CPE*	0.565	4.08E-02
ART3	0.241	3.57E-02	*NXN*	0.365	4.08E-02
ZNF311	-0.442	3.57E-02	*TNPO3*	-0.445	4.08E-02
RARRES2	0.793	3.58E-02	*MED27*	-0.465	4.08E-02
SIRPB2	0.687	3.58E-02	*BTN3A2*	0.693	4.09E-02
PCOLCE2	0.312	3.58E-02	*ADAMTS5*	0.520	4.09E-02
MARK4	-0.642	3.58E-02	*ACO1*	0.510	4.09E-02
TEF	-0.644	3.58E-02	*COMP*	0.475	4.09E-02
C12orf52	-0.705	3.58E-02	*SLC25A14*	-0.395	4.09E-02
BIN1	0.718	3.59E-02	*PAX9*	-0.400	4.09E-02
CDC42BPA	-0.586	3.60E-02	*SAPCD2*	-0.527	4.09E-02
SGPP1	1.096	3.61E-02	*C1orf53*	-0.437	4.10E-02
DDX26B	0.893	3.61E-02	*C9orf47*	-0.450	4.10E-02
RRAS2	0.550	3.61E-02	*SNX9*	0.613	4.11E-02
MTA3	-0.523	3.61E-02	*GFI1*	0.410	4.11E-02
EFHD2	1.406	3.62E-02	*CPNE5*	0.347	4.11E-02
ADAM19	0.880	3.62E-02	*LIPG*	0.215	4.11E-02

BATF	0.871	3.62E-02	*ZNF677*	0.190	4.11E-02
MARC1	-0.517	3.62E-02	*THNSL2*	-0.388	4.11E-02
FAM63A	-0.617	3.62E-02	*MARK4*	-0.465	4.11E-02
C1orf64	-0.654	3.62E-02	*ANKRD26*	-0.485	4.11E-02
TRIM38	1.390	3.63E-02	*LCK*	0.593	4.12E-02
FAM117A	0.593	3.63E-02	*TIFAB*	0.573	4.12E-02
KIAA1383	-0.468	3.63E-02	*C4orf32*	0.427	4.13E-02
EDEM1	0.872	3.64E-02	*SLC43A1*	0.338	4.13E-02
PPP1R14A	-0.535	3.64E-02	*HOXB7*	0.128	4.13E-02
TCAM1P	0.198	3.65E-02	*DPM3*	-0.520	4.13E-02
AGAP11	-0.467	3.65E-02	*STARD8*	0.580	4.14E-02
FAM110B	-0.531	3.65E-02	*MYO1B*	0.570	4.14E-02
ATF7IP2	0.557	3.66E-02	*SLIT2*	0.540	4.14E-02
PPAPDC1A	0.448	3.66E-02	*CYTL1*	0.245	4.14E-02
TFPI2	0.428	3.66E-02	*RAB38*	0.195	4.14E-02
TNNC1	-0.452	3.66E-02	*KCNQ5*	-0.317	4.14E-02
MCF2L-AS1	-0.535	3.66E-02	*ZNF354C*	-0.327	4.14E-02
CHAD	-0.601	3.66E-02	*CCDC121*	-0.430	4.14E-02
IFI35	1.474	3.67E-02	*MOV10L1*	-0.443	4.14E-02
FOXO1	0.681	3.67E-02	*HCN3*	-0.467	4.14E-02
DDIT4L	-0.460	3.67E-02	*ADAD2*	-0.547	4.14E-02
LCE1E	-0.598	3.67E-02	*DNASE1L3*	0.345	4.15E-02
HPN	-0.614	3.67E-02	*NOSTRIN*	0.333	4.15E-02
PTGER2	0.452	3.68E-02	*C1orf220*	-0.403	4.15E-02
ATP7B	-0.558	3.68E-02	*CHTF18*	-0.547	4.15E-02
SH3BP5	0.624	3.69E-02	*PTPRG*	0.453	4.16E-02
STARD13	-0.578	3.69E-02	*EPN2*	-0.380	4.16E-02
R3HDML	-0.638	3.69E-02	*CACNB2*	-0.418	4.16E-02
DGAT1	-0.687	3.69E-02	*MTERFD1*	-0.553	4.16E-02
BST1	0.636	3.70E-02	*CALHM2*	0.688	4.17E-02
ABCA11P	-0.563	3.70E-02	*EPHA7*	-0.363	4.17E-02
COL15A1	0.723	3.71E-02	*GRIK1-AS1*	-0.387	4.17E-02
RUSC2	-0.706	3.71E-02	*LDB1*	-0.438	4.17E-02
MBP	0.480	3.72E-02	*C14orf149*	0.413	4.18E-02
SLC9B2	0.435	3.72E-02	*ENPEP*	0.248	4.18E-02
KIR3DL3	0.410	3.72E-02	*IL13RA2*	0.230	4.18E-02
LOC84856	0.362	3.72E-02	*PPP2R2D*	-0.353	4.18E-02
RAB38	0.296	3.72E-02	*ADIPOR1*	-0.423	4.18E-02
CCDC153	-0.604	3.72E-02	*NDUFB9*	-0.493	4.18E-02
AARS2	-0.645	3.72E-02	*IFNAR2*	0.433	4.19E-02
C10orf54	1.545	3.73E-02	*FAM124B*	0.415	4.19E-02
MYD88	1.249	3.73E-02	*RAP1GAP2*	-0.328	4.19E-02
RUNX1-IT1	0.651	3.73E-02	*RFC3*	-0.550	4.19E-02
AGMAT	0.424	3.73E-02	*BPNT1*	-0.417	4.20E-02
TMPRSS3	0.278	3.73E-02	*THAP7*	-0.498	4.20E-02
HOXB6	-0.393	3.73E-02	*CYR61*	0.643	4.21E-02
DOCK1	-0.581	3.73E-02	*LRCH2*	0.487	4.21E-02
CCDC121	-0.590	3.73E-02	*ARFIP2*	-0.450	4.21E-02
TSPY26P	-0.609	3.73E-02	*NAAA*	0.405	4.22E-02
CCNYL1	1.057	3.74E-02	*SCN3A*	0.243	4.22E-02
SH2D4A	-0.471	3.74E-02	*C15orf41*	-0.380	4.22E-02

ACAD10	-0.642	3.74E-02	*PTPN7*	0.580	4.23E-02
FGR	1.337	3.75E-02	*MTUS1*	0.495	4.23E-02
STX11	1.062	3.75E-02	*LGALS2*	0.413	4.23E-02
EPHB2	0.817	3.75E-02	*LAX1*	0.408	4.23E-02
PSAT1	0.545	3.75E-02	*MFHAS1*	0.300	4.23E-02
CYBA	1.235	3.76E-02	*IFT172*	-0.413	4.23E-02
CHST7	0.383	3.76E-02	*MRGPRF*	0.573	4.24E-02
CGN	-0.583	3.76E-02	*TREX2*	-0.465	4.24E-02
CD84	1.227	3.77E-02	*LPAR2*	-0.470	4.24E-02
C12orf50	0.189	3.77E-02	*CEBPA*	0.438	4.25E-02
DCDC2	-0.454	3.78E-02	*TSPAN2*	0.350	4.25E-02
CD3E	1.207	3.79E-02	*OR51B2*	-0.490	4.25E-02
C5AR1	0.915	3.79E-02	*LTBP2*	0.860	4.26E-02
NDRG3	-0.552	3.79E-02	*SYNPO2*	0.637	4.26E-02
ZNF84	-0.744	3.79E-02	*ZNF516*	-0.415	4.26E-02
LRRC2	0.282	3.80E-02	*RRNAD1*	-0.483	4.26E-02
GCA	1.107	3.81E-02	*HPSE*	0.383	4.27E-02
FAIM3	0.810	3.81E-02	*PDE4DIP*	0.265	4.27E-02
GAS1	0.686	3.81E-02	*VPRBP*	-0.423	4.27E-02
MAP7	-0.533	3.81E-02	*CYHR1*	-0.510	4.27E-02
SLC25A43	0.838	3.82E-02	*SIRPG*	0.498	4.28E-02
ERMN	0.272	3.82E-02	*CDR1*	0.165	4.28E-02
PECAM1	1.122	3.83E-02	*KAT2A*	-0.480	4.28E-02
ANKFY1	0.919	3.83E-02	*CHCHD6*	-0.480	4.28E-02
CD180	0.861	3.83E-02	*EPHB2*	0.560	4.29E-02
IGFBP3	0.593	3.83E-02	*RARRES1*	0.348	4.29E-02
KIAA1328	-0.454	3.83E-02	*RASSF6*	0.177	4.29E-02
TRAF3IP2	-0.476	3.83E-02	*SEMA3C*	0.605	4.30E-02
ATPBD4	-0.626	3.83E-02	*IL6*	0.530	4.30E-02
GRIN2C	-0.629	3.83E-02	*PFKFB3*	0.448	4.30E-02
LPCAT2	0.885	3.84E-02	*GRK4*	-0.393	4.30E-02
LINC00341	0.601	3.84E-02	*UBXN2A*	-0.403	4.30E-02
PP7080	-0.547	3.85E-02	*S100A4*	0.635	4.31E-02
SFXN4	-0.673	3.85E-02	*TCEAL7*	0.397	4.31E-02
PTPN6	1.170	3.86E-02	*C1orf74*	-0.373	4.31E-02
SLC6A6	0.604	3.86E-02	*KIF20A*	-0.515	4.31E-02
VPS37C	-0.548	3.86E-02	*LOXL1*	0.565	4.32E-02
TBX21	0.825	3.87E-02	*TMEM204*	0.493	4.32E-02
RBM24	-0.494	3.87E-02	*UBE2E3*	0.435	4.32E-02
NCDN	-0.665	3.87E-02	*AIM1*	0.398	4.32E-02
TNFRSF11A	0.281	3.88E-02	*FILIP1*	0.310	4.32E-02
AGPAT6	-0.501	3.88E-02	*NUAK2*	0.268	4.32E-02
LIN7B	-0.596	3.88E-02	*ZNF804A*	0.178	4.32E-02
RARRES3	1.069	3.89E-02	*LAMB2P1*	-0.460	4.32E-02
PTPRS	-0.578	3.89E-02	*LST1*	0.840	4.33E-02
PLXDC2	0.779	3.90E-02	*TINF2*	0.623	4.33E-02
ABCC1	0.557	3.90E-02	*ANKFY1*	0.600	4.33E-02
SGPP2	0.274	3.90E-02	*SPOCD1*	0.520	4.33E-02
IL12RB2	0.268	3.90E-02	*DDB2*	0.380	4.33E-02
CSPP1	-0.669	3.90E-02	*PPAP2C*	-0.390	4.33E-02
PPARGC1B	0.363	3.91E-02	*JUP*	-0.425	4.33E-02

PCDHGB7	-0.416	3.91E-02	*MRGPRX2*	-0.437	4.33E-02
SRG7	-0.482	3.91E-02	*PPFIA3*	-0.475	4.33E-02
HEATR6	-0.505	3.91E-02	*RBMS1*	0.715	4.34E-02
PIK3C2G	-0.581	3.91E-02	*LRRK1*	0.328	4.35E-02
KAT2A	-0.662	3.91E-02	*PNLIPRP3*	0.190	4.35E-02
CHFR	0.608	3.92E-02	*PRSS16*	-0.363	4.35E-02
ZNF717	-0.553	3.92E-02	*BATF3*	0.463	4.36E-02
SLX4	-0.648	3.92E-02	*JAKMIP2*	0.210	4.36E-02
TMEM87A	-0.682	3.92E-02	*HOTAIR*	-0.287	4.36E-02
NUMA1	-0.713	3.92E-02	*CAMTA1*	-0.343	4.36E-02
IRF9	1.436	3.93E-02	*IRAK1BP1*	-0.443	4.36E-02
IL33	0.494	3.93E-02	*CD248*	0.755	4.37E-02
SGCZ	-0.468	3.93E-02	*CD33*	0.705	4.37E-02
LACE1	-0.442	3.94E-02	*RGS4*	0.643	4.37E-02
NACA2	-0.500	3.94E-02	*RUNX1T1*	0.623	4.37E-02
POF1B	-0.454	3.95E-02	*TRAM1L1*	-0.380	4.37E-02
KIAA1671	-0.470	3.95E-02	*BCMO1*	-0.395	4.37E-02
ZNF35	-0.490	3.95E-02	*PBX1*	-0.438	4.37E-02
SRSF6	-0.615	3.95E-02	*GNS*	0.943	4.38E-02
KRT32	-0.659	3.95E-02	*ATP8B2*	0.478	4.38E-02
VASH1	0.692	3.96E-02	*SLC35G2*	0.353	4.38E-02
CBLN3	0.592	3.96E-02	*C18orf34*	0.347	4.38E-02
GDEP	-0.585	3.96E-02	*BAIAP2*	-0.470	4.38E-02
DIP2B	-0.643	3.96E-02	*MSRB3*	0.680	4.39E-02
PPP3CC	0.913	3.97E-02	*KLF10*	0.638	4.39E-02
TNFRSF10B	0.600	3.97E-02	*TTPA*	-0.348	4.39E-02
FAM54B	-0.650	3.97E-02	*C2orf61*	-0.353	4.39E-02
REPIN1	-0.570	3.98E-02	*C4orf3*	0.773	4.40E-02
CCDC78	-0.609	3.98E-02	*CXCL10*	0.520	4.40E-02
ANKRD50	-0.620	3.98E-02	*STBD1*	0.305	4.40E-02
FLJ13197	-0.649	3.98E-02	*CALD1*	0.805	4.41E-02
ACOT9	0.969	3.99E-02	*KDELC2*	0.650	4.41E-02
RASGRP2	0.825	3.99E-02	*TSPAN11*	0.377	4.41E-02
DGCR2	-0.563	3.99E-02	*IL12RB2*	-0.323	4.41E-02
RPL32P3	-0.590	3.99E-02	*TMEM194A*	-0.510	4.41E-02
RAB8B	1.581	4.00E-02	*GCH1*	0.638	4.42E-02
DKK1	0.414	4.00E-02	*ERVW-1*	0.140	4.42E-02
LOC90246	-0.539	4.00E-02	*SELPLG*	0.890	4.43E-02
RASSF9	0.472	4.01E-02	*CAV2*	0.650	4.43E-02
PDK1	0.452	4.01E-02	*CALCRL*	0.550	4.43E-02
FAIM2	-0.669	4.01E-02	*PLA2G4C*	0.450	4.43E-02
FCAMR	0.423	4.02E-02	*TMEM132C*	0.353	4.43E-02
STUB1	-0.752	4.02E-02	*NPTX2*	0.158	4.43E-02
UPP1	0.865	4.03E-02	*PFDN4*	-0.485	4.43E-02
SPATS2L	0.783	4.03E-02	*CDKN1A*	0.715	4.44E-02
GNA12	0.562	4.03E-02	*SETD7*	0.623	4.44E-02
PYDC1	0.277	4.03E-02	*NFKB1*	0.615	4.44E-02
CDH20	-0.488	4.03E-02	*KIAA0930*	0.533	4.44E-02
TSNARE1	-0.628	4.03E-02	*ABCG2*	0.223	4.44E-02
DOK3	1.007	4.04E-02	*DEPDC5*	-0.383	4.44E-02
PRRX1	0.924	4.04E-02	*PLIN1*	0.610	4.45E-02

EGR3	0.432	4.04E-02	*AMIGO2*	0.395	4.45E-02
LINC00314	-0.453	4.04E-02	*EDC3*	-0.438	4.45E-02
CXXC4	-0.473	4.04E-02	*DEFB1*	0.248	4.46E-02
RBPMS2	-0.476	4.04E-02	*KCNRG*	-0.307	4.46E-02
IRF4	0.982	4.05E-02	*ZNF711*	-0.423	4.46E-02
CHL1	0.358	4.05E-02	*CRYBG3*	0.613	4.47E-02
KRT6C	0.210	4.05E-02	*SNX10*	0.593	4.47E-02
SNORA74A	-0.594	4.05E-02	*PLN*	0.453	4.47E-02
SNTA1	-0.704	4.05E-02	*KIAA0125*	0.383	4.47E-02
EPB41L2	0.723	4.06E-02	*CBFA2T3*	0.240	4.47E-02
SIRPB1	0.720	4.06E-02	*ARX*	0.160	4.47E-02
TRIM47	0.762	4.07E-02	*IPPK*	-0.390	4.47E-02
B3GNT5	0.660	4.07E-02	*LLGL2*	-0.425	4.47E-02
CLIC5	0.565	4.07E-02	*PPM1M*	0.770	4.48E-02
GRHL3	0.402	4.07E-02	*SLC35G3*	-0.293	4.48E-02
PLCE1	-0.471	4.07E-02	*ZKSCAN2*	-0.343	4.48E-02
ZNF471	-0.493	4.07E-02	*ANKRD16*	-0.427	4.48E-02
ZNF101	0.539	4.08E-02	*LIG3*	-0.455	4.48E-02
MOXD1	0.710	4.09E-02	*HIST1H4C*	-0.465	4.48E-02
KAT8	-0.561	4.09E-02	*TIPIN*	-0.540	4.48E-02
GSTZ1	-0.615	4.09E-02	*MYCBP2*	0.788	4.49E-02
LY9	1.059	4.10E-02	*NUAK1*	0.558	4.49E-02
NAT8L	-0.570	4.10E-02	*PDE1A*	0.468	4.49E-02
NELL1	-0.584	4.10E-02	*GNLY*	0.410	4.49E-02
AGBL5	-0.627	4.10E-02	*TRIM69*	0.327	4.49E-02
C17orf61	-0.643	4.10E-02	*KCNMB4*	0.273	4.49E-02
STARD10	-0.651	4.10E-02	*FOXD2*	-0.403	4.49E-02
ARHGAP27	0.837	4.11E-02	*FZD9*	-0.520	4.49E-02
C11orf87	-0.474	4.11E-02	*PPIC*	0.640	4.50E-02
ADIG	-0.719	4.11E-02	*CXCL2*	0.470	4.50E-02
CHD5	-0.726	4.11E-02	*UBE2E2*	0.440	4.50E-02
C19orf66	1.233	4.12E-02	*DCLK1*	0.418	4.50E-02
SEPT1	0.704	4.12E-02	*GXYLT2*	0.407	4.50E-02
BDH1	-0.595	4.13E-02	*EBF3*	0.280	4.50E-02
TELO2	-0.776	4.13E-02	*SLC26A3*	0.153	4.50E-02
MEOX2	0.348	4.14E-02	*PI4K2A*	0.560	4.51E-02
TIFA	0.645	4.15E-02	*ANKH*	0.288	4.51E-02
FAM129C	0.514	4.15E-02	*INPP5D*	0.630	4.52E-02
KSR2	-0.540	4.15E-02	*RNF144A*	0.328	4.52E-02
TRIM25	0.648	4.16E-02	*PDGFA*	0.248	4.52E-02
CCDC65	-0.497	4.16E-02	*CCDC138*	-0.517	4.52E-02
CRLF1	-0.538	4.16E-02	*FNIP2*	0.557	4.53E-02
MAN2A2	-0.588	4.16E-02	*LRRC33*	0.440	4.53E-02
ARRDC2	0.801	4.17E-02	*ITGB6*	0.278	4.53E-02
SLC24A4	0.406	4.17E-02	*FBXL7*	0.530	4.54E-02
MB21D2	0.353	4.17E-02	*TMEM2*	0.493	4.54E-02
BTRC	-0.497	4.17E-02	*MZB1*	0.465	4.54E-02
STK4	0.879	4.18E-02	*ANXA2R*	0.277	4.54E-02
GJA5	0.606	4.18E-02	*TOP2A*	-0.530	4.54E-02
KRT6A	0.464	4.18E-02			
CCDC67	-0.492	4.18E-02			

INADL	-0.551	4.18E-02
ARHGEF25	-0.564	4.18E-02
HECW2	0.449	4.19E-02
EID3	0.323	4.19E-02
CCBE1	0.463	4.21E-02
GPR82	0.337	4.21E-02
GJA9	-0.533	4.21E-02
CSNK1E	-0.601	4.21E-02
NCEH1	0.982	4.22E-02
AKIRIN2	0.823	4.22E-02
ZNFX1	1.387	4.23E-02
PPAP2B	0.747	4.23E-02
ARHGAP26	0.568	4.23E-02
FILIP1	0.415	4.23E-02
CD40LG	0.914	4.24E-02
TNFSF14	0.802	4.24E-02
ZAP70	0.961	4.25E-02
NECAB1	0.169	4.25E-02
RPAP1	-0.603	4.25E-02
CSDA	0.461	4.26E-02
PARM1	0.457	4.26E-02
SLC6A14	0.243	4.26E-02
PROK2	0.216	4.26E-02
AK7	-0.466	4.26E-02
GLB1L2	-0.489	4.26E-02
ZNF214	-0.532	4.26E-02
TECPR2	-0.581	4.26E-02
DPYD	1.384	4.27E-02
BTN2A1	0.778	4.27E-02
CPT1A	-0.392	4.27E-02
BEND2	-0.456	4.27E-02
SERPINA1	0.837	4.28E-02
CAMSAP3	-0.687	4.28E-02
DOK2	1.268	4.29E-02
CREM	0.645	4.29E-02
ALPK1	0.955	4.30E-02
RNASE2	0.637	4.30E-02
TRIM36	-0.451	4.30E-02
RGS16	0.493	4.31E-02
TNFSF9	0.365	4.31E-02
PPARGC1A	-0.480	4.31E-02
PRR15L	-0.563	4.31E-02
KIAA1737	-0.653	4.31E-02
STAT2	1.131	4.32E-02
SLC8A1	0.953	4.32E-02
SAPCD1	-0.645	4.32E-02
AQP9	0.928	4.33E-02
ARMC9	-0.428	4.33E-02
ST6GALNAC2	-0.499	4.33E-02
ZSCAN2	-0.524	4.33E-02
ABHD12	-0.556	4.33E-02

CLEC4F	-0.564	4.33E-02
FGF19	-0.584	4.33E-02
FLJ44342	-0.662	4.33E-02
LINC00589	-0.683	4.33E-02
C11orf35	-0.720	4.33E-02
BACE2	0.404	4.34E-02
CA12	-0.504	4.34E-02
CXCL12	0.881	4.35E-02
KCNJ10	0.816	4.35E-02
ENPP6	0.188	4.35E-02
FGFRL1	-0.619	4.35E-02
IFI27L2	0.827	4.36E-02
LMO4	0.295	4.36E-02
VEGFC	0.744	4.37E-02
S100A9	0.697	4.37E-02
BEND7	-0.455	4.37E-02
IL17D	-0.528	4.37E-02
METRN	-0.689	4.37E-02
CD68	1.063	4.38E-02
TBC1D1	0.411	4.38E-02
SPIC	0.059	4.38E-02
SPATA6L	-0.482	4.38E-02
BEST3	-0.528	4.38E-02
RNF141	-0.631	4.38E-02
ABCA1	0.931	4.39E-02
RAB7L1	0.599	4.39E-02
POLH	-0.481	4.39E-02
IPW	-0.637	4.39E-02
CPM	0.421	4.40E-02
PGAP2	-0.578	4.40E-02
CDC42BPB	-0.599	4.40E-02
XIRP1	0.807	4.41E-02
GABRG1	-0.501	4.41E-02
TMEM201	-0.671	4.41E-02
ABI3BP	0.760	4.42E-02
ZNF625	-0.459	4.42E-02
CMYA5	-0.556	4.42E-02
KRTAP17-1	-0.691	4.43E-02
BLK	0.487	4.44E-02
C15orf26	-0.424	4.44E-02
FAM81A	-0.474	4.44E-02
MIR600HG	-0.609	4.44E-02
DACT1	0.499	4.45E-02
FAM9B	-0.432	4.45E-02
RASSF8	-0.454	4.45E-02
C9orf24	-0.584	4.45E-02
KLHDC10	-0.498	4.46E-02
ACSL4	1.153	4.47E-02
NUS1	0.582	4.47E-02
RBPJ	0.552	4.47E-02
ATP11A	0.398	4.47E-02

SPCS3	1.165	4.48E-02
KCNT2	0.591	4.48E-02
GNL1	-0.530	4.48E-02
S100A4	0.894	4.49E-02
ADPRH	0.747	4.49E-02
ZNF804A	0.251	4.50E-02
M6PR	1.064	4.51E-02
RGL1	1.003	4.51E-02
KLHL29	0.392	4.51E-02
TMEM232	-0.456	4.51E-02
SULF1	0.916	4.52E-02
ING1	0.328	4.52E-02
C1orf101	-0.538	4.52E-02
PPM1J	-0.552	4.52E-02
MID2	-0.592	4.52E-02
PPP2R2B	0.302	4.53E-02
TEKT2	-0.560	4.53E-02
C10orf116	-0.574	4.53E-02
STK10	1.217	4.54E-02
LPAR6	0.946	4.54E-02
CYFIP2	0.602	4.54E-02
CRIP2	-0.589	4.54E-02
NUDT14	-0.628	4.54E-02
ADAMTS18	-0.630	4.54E-02

Supplementary Table 6. Univariate Cox regression analysis of AXL and CD163 for relapse-free survival and overall survival in triple-negative breast cancer (N = 203).

Variable	***HR***	***95% CI***	**P-*value*[a]**	***HR***	***95% CI***	**P-*value*[a]**
	Relapse-Free Survival			***Overall Survival***		
AXL	3.19	1.66 – 6.14	**5.00E-04**	3.16	1.64 – 6.08	**6.00E-04**
CD163	2.02	1.06 – 3.85	**3.29E-02**	1.75	0.92 – 3.31	8.65E-02

Abbreviations: CI, Confidence interval; HR, Hazard ratio.
[a]Significant *P*-values are given in bold.

Supplementary Table 7. Multivariate Cox regression analysis of AXL for relapse-free survival and overall survival in the validation cohort of triple-negative breast cancer (N = 95)

Variable	***HR***	***95% CI***	**P-*value*[a]**	***HR***	***95% CI***	**P-*value*[a]**
	Relapse-Free Survival			***Overall Survival***		
AXL	6.05	1.93 – 18.72	**1.80E-03**	6.09	2.12 – 17.54	**8.00E-04**
Age	0.87	0.32 – 2.36	7.86E-01	0.89	0.35 – 2.25	8.01E-01
Grade	1.54	0.42 – 5.57	5.12E-01	2.01	0.55 – 7.32	2.90E-01
Nodal status	5.96	1.16 – 30.57	**3.20E-02**	3.99	1.05 – 15.19	**4.20E-02**
Tumor size	1.69	0.63 – 4.54	2.96E-01	1.43	0.53 – 3.85	4.76E-01
Tumor stage	1.12	0.36 – 3.51	8.44E-01	1.01	0.31 – 3.21	9.93E-01

Abbreviations: CI, Confidence interval; HR, Hazard ratio.
[a]Significant *P*-values are given in bold.

Supplementary Table 8. Multivariate Cox regression analysis of AXL evaluated as a continuous variable for relapse-free survival and overall survival in the discovery (N = 203) and validation (N = 95) cohort of triple-negative breast cancer.

Variable	***HR***	***95% CI***	**P-*value*[a]**	***HR***	***95% CI***	**P-*value*[a]**
		Relapse-Free Survival			***Overall Survival***	
			**Discovery cohort**			
AXL	1.25	1.07 – 1.46	**3.39E-03**	1.32	1.13 – 1.53	**5.00E-04**
Age	0.95	0.50 – 1.82	8.81E-01	1.09	0.57 – 2.07	8.05E-01
Grade	1.60	0.65 – 3.90	3.04E-01	1.93	0.78 – 4.74	1.54E-01
Nodal status	2.67	1.07 – 6.67	**3.49E-02**	1.37	0.61 – 3.08	4.42E-01
Tumor size	1.51	0.78 – 2.91	2.18E-01	1.14	0.60 – 2.17	6.91E-01
Tumor stage	1.71	0.80 – 3.68	1.66E-01	1.22	0.52 – 2.87	6.49E-01
			**Validation cohort**			
AXL	1.42	1.12 – 1.81	**4.44E-03**	1.44	1.13 – 1.83	**3.00E-03**
Age	1.20	0.44 – 3.30	7.23E-01	1.24	0.47 – 3.26	6.65E-01
Grade	1.54	0.42 – 5.62	5.14E-01	1.91	0.53 – 6.97	3.25E-01
Nodal status	5.65	1.06 – 30.24	**4.30E-02**	3.44	0.88 – 13.42	7.58E-02
Tumor size	1.47	0.51 – 4.27	4.78E-01	1.20	0.43 – 3.35	7.34E-01
Tumor stage	0.97	0.32 – 2.93	9.52E-01	0.88	0.29 – 2.65	8.23E-01

Abbreviations: CI, Confidence Interval; HR, Hazard Ratio.
[a]Significant *P*-values (Fisher's exact test) are given in bold.
Multivariate analysis adjusted for age (≥50 *v* <50), histological grade (G3 *v* G1-2), nodal status (1 *v* 0), tumor size (> 20 mm *v* ≤ 20 mm), and tumor stage (III *v* I-II).

Supplementary Table 9. List of TaqMan gene expression assays used in this study.

Assay ID	***Gene Symbol***	***Gene Name***
Hs00178289_m1	*AKT1*	v-akt murine thymoma viral oncogene homolog 1
Hs01086102_m1	*AKT2*	v-akt murine thymoma viral oncogene homolog 2
Hs01064444_m1	*AXL*	AXL receptor tyrosine kinase
Hs00234140_m1	*CCL2*	chemokine (C-C motif) ligand 2
Hs00174575_m1	*CCL5*	chemokine (C-C motif) ligand 5
Hs01023894_m1	*CDH1*	E- cadherin
Hs00174164_m1	*CSF1*	colony stimulating factor 1
Hs00171266_m1	*CSF2*	colony stimulating factor 2
Hs00171042_m1	*CXCL10*	chemokine (C-X-C motif) ligand 10
Hs01058430_m1	*DDR1*	discoidin domain receptor tyrosine kinase 1
Hs01025953_m1	*DDR2*	discoidin domain receptor tyrosine kinase 2
Hs01076078_m1	*EGFR*	epidermal growth factor receptor
Hs00946872_m1	*ERK1*	mitogen-activated protein kinase 3
Hs01046830_m1	*ERK2*	mitogen-activated protein kinase 1
Hs01056457_m1	*FAK*	protein tyrosine kinase 2
Hs00915142_m1	*FGFR1*	fibroblast growth factor receptor 1
Hs01552926_m1	*FGFR2*	fibroblast growth factor receptor 2
Hs00179829_m1	*FGFR3*	fibroblast growth factor receptor 3
Hs00941600_m1	*FYN*	FYN oncogene related to SRC
Hs02758991_g1	*GAPDH*	glyceraldehyde-3-phosphate dehydrogenase
Hs01047719_m1	*GSK3B*	glycogen synthase kinase 3 beta
Hs00174143_m1	*IFNG*	interferon gamma
Hs00609566_m1	*IGF1R*	insulin-like growth factor 1 receptor
Hs00989502_m1	*IKKA*	conserved helix-loop-helix ubiquitous kinase
Hs00233287_m1	*IKKB*	inhibitor of kappa light polypeptide gene enhancer in B-cells, kinase beta
Hs00174086_m1	*IL10*	interleukin 10
Hs00174379_m1	*IL13*	interleukin 13
Hs00174122_m1	*IL4*	interleukin 4
Hs00174131_m1	*IL6*	interleukin 6
Hs00174103_m1	*IL8*	interleukin 8
Hs00176719_m1	*LYN*	LYN proto-oncogene, Src family tyrosine kinase
Hs00361185_m1	*KRT5*	keratin 5, type II
Hs04194231_s1	*KRT6A*	keratin 6A, type II
Hs01565584_m1	*MET*	met proto-oncogene
Hs00968300_g1	*OSM*	oncostatin M
Hs00998018_m1	*PDGFRA*	platelet-derived growth factor receptor, alpha polypeptide
Hs01019589_m1	*PDGFRB*	platelet-derived growth factor receptor, beta polypeptide
Hs00169444_m1	*PTK2B*	protein tyrosine kinase 2 beta

Hs01082246_m1	*SRC*	SRC proto-oncogene, non-receptor tyrosine kinase
Hs00895377_m1	*SYK*	spleen tyrosine kinase
Hs00171257_m1	*TGFB1*	transforming growth factor beta 1
Hs00234244_m1	*TGFB2*	transforming growth factor beta 2
Hs00610320_m1	*TGFBR1*	transforming growth factor, beta receptor 1
Hs00234253_m1	*TGFBR2*	transforming growth factor, beta receptor 2
Hs01052961_m1	*VEGFR1*	fms-related tyrosine kinase 1
Hs00911700_m1	*VEGFR2*	kinase insert domain receptor
Hs01047677_m1	*VEGFR3*	fms-related tyrosine kinase 4
Hs00185584_m1	*VIM*	vimentin
Hs01080045_g1	*YES1*	YES proto-oncogene 1, Src family tyrosine kinase
