# Supplementary material for: AXL-associated tumor inflammation as a poor prognostic signature in chemotherapy-treated triple-negative breast cancer patients
Source: NPJ Breast Cancer. 2016 Nov 2;2:16033–. doi: 10.1038/npjbcancer.2016.33 (PMC5515347; doi:10.1038/npjbcancer.2016.33)
Supplement: Supplementary Figures [file npjbcancer201633-s3.pdf]

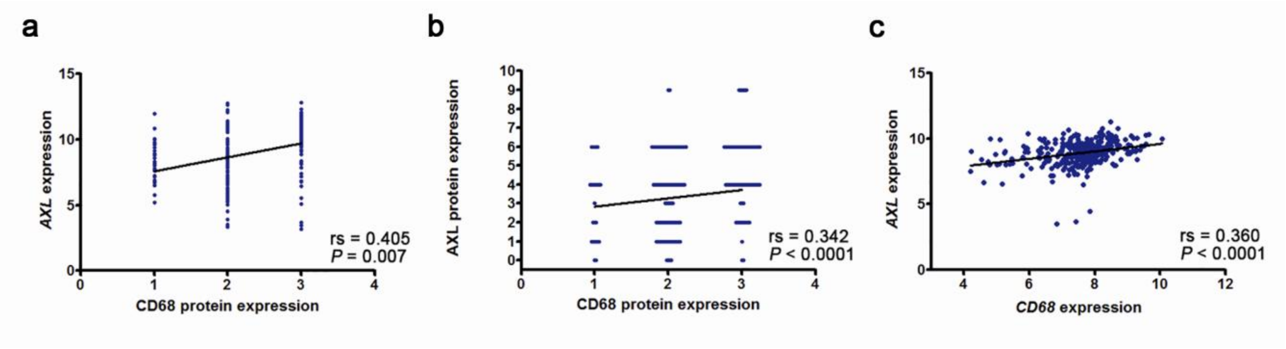

Supplementary Figure 1. AXL expression correlates with CD68 expression in triple-negative breast cancer. a) Correlation analysis between *AXL* gene expression and CD68 protein in the internal cohort of 203 TNBC (Spearman's coefficient, rs = 0.405; Bonferroni adjusted $P = 0.007$). b) A scatter diagram showing the positive correlation between immunohistochemical staining of AXL and CD163 proteins in TNBC (N = 203) (rs = 0.342; $P < 0.0001$). c) Correlation analysis between the expression of *AXL* and *CD68* performed on gene expression data from 3 publicly available TNBC datasets (N = 311) (rs = 0.360; $P < 0.0001$).

a

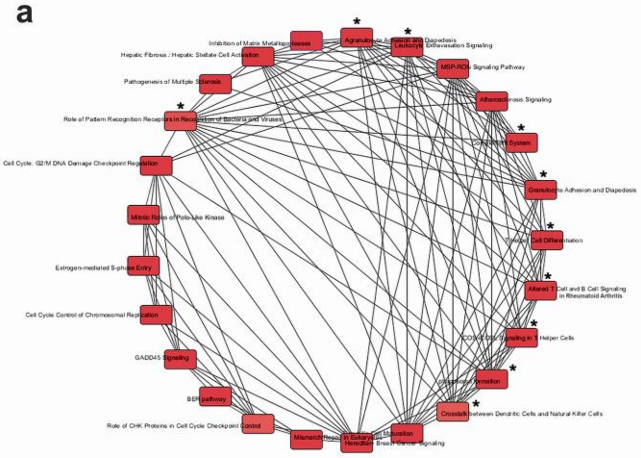

b

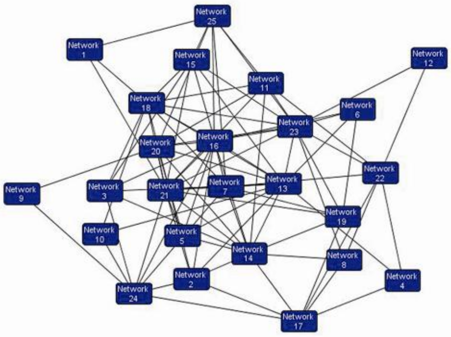

Network 1 Hematological Disease, Infectious Diseases, Organismal Injury and Abnormalities
Network 2 Antimicrobial Response, Inflammatory Response, Infectious Diseases
Network 3 Cell-To-Cell Signaling and Interaction, Hematological System Development and Function, Immune Cell Trafficking
Network 4 Cell-To-Cell Signaling and Interaction, Hematological System Development and Function, Immune Cell Trafficking
Network 5 Cell-To-Cell Signaling and Interaction, Hematological System Development and Function, Immune Cell Trafficking
Network 6 Lipid Metabolism, Molecular Transport, Small Molecule Biochemistry
Network 7 Endocrine System Disorders, Gastrointestinal Disease, Immunological Disease
Network 8 Hematological System Development and Function, Tissue Morphology, Lymphoid Tissue Structure and Development
Network 9 Cellular Assembly and Organization, Cellular Function and Maintenance, Protein Degradation
Network 10 Cell-To-Cell Signaling and Interaction, Hematological System Development and Function, Immune Cell Trafficking
Network 11 Cellular Movement, Immune Cell Trafficking, Hematological System Development and Function
Network 12 Developmental Disorder, Hereditary Disorder, Neurological Disease
Network 13 Immunological Disease, Endocrine System Disorders, Gastrointestinal Disease
Network 14 Antimicrobial Response, Inflammatory Response, Infectious Diseases
Network 15 Cell Death and Survival, Dermatological Diseases and Conditions, Cancer
Network 16 Antimicrobial Response, Inflammatory Response, Infectious Diseases
Network 17 Hereditary Disorder, Neurological Disease, Organismal Injury and Abnormalities
Network 18 Inflammatory Response, Cell-To-Cell Signaling and Interaction, Cellular Growth and Proliferation
Network 19 Cellular Development, Hematological System Development and Function, Hematopoiesis
Network 20 Cell Death and Survival, Embryonic Development, Organismal Development
Network 21 Cellular Function and Maintenance, Hematological System Development and Function, Immune Cell Trafficking
Network 22 Cardiac Arteriopathy, Cardiovascular Disease, Organismal Injury and Abnormalities
Network 23 Cell Death and Survival, Gene Expression, Organismal Development
Network 24 Cellular Growth and Proliferation, Cellular Development, Hematological System Development and Function
Network 25 Cellular Assembly and Organization, Cellular Function and Maintenance, Cellular Growth and Proliferation

c

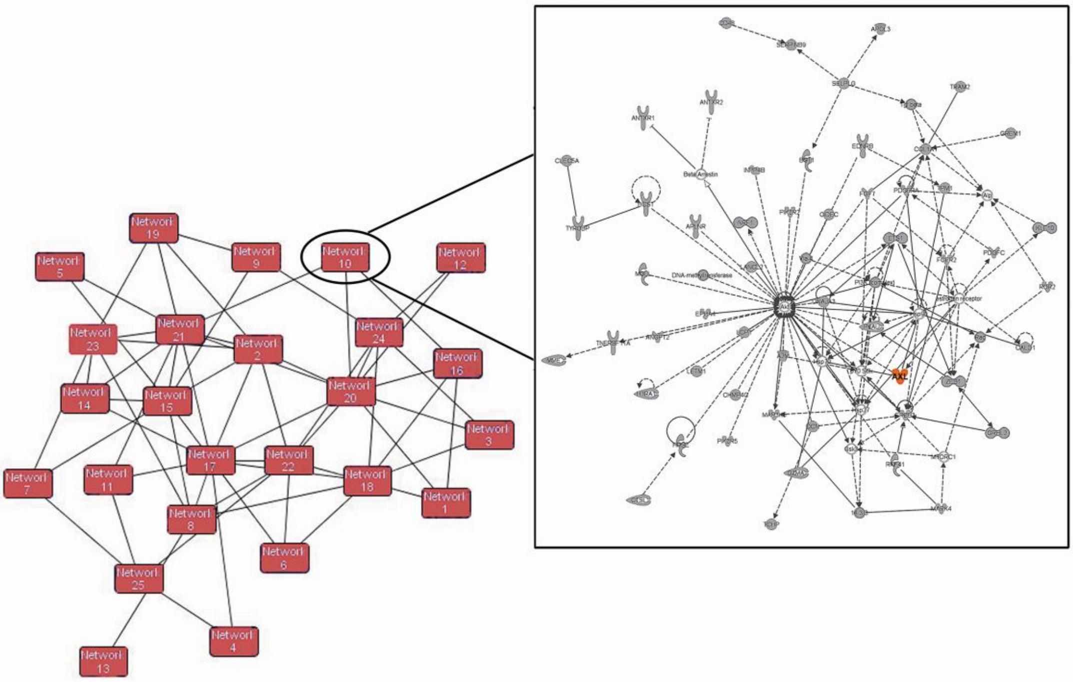

Network 1 Cellular Development, Hematological System Development and Function, Hematopoiesis
Network 2 Cell Death and Survival, Cell Cycle, DNA Replication, Recombination, and Repair
Network 3 Cardiovascular Disease, Developmental Disorder, Hematological Disease
Network 4 Cellular Movement, Cell Morphology, Cellular Growth and Proliferation
Network 5 Lipid Metabolism, Molecular Transport, Small Molecule Biochemistry
Network 6 Cellular Movement, Cardiovascular System Development and Function, Organismal Development
Network 7 Cellular Function and Maintenance, Cellular Growth and Proliferation, Hematological System Development and Function
Network 8 Cellular Movement, Immune Cell Trafficking, Cellular Function and Maintenance
Network 9 Cancer, Organismal Injury and Abnormalities, Cell Death and Survival
Network 10 Cellular Movement, Cell Death and Survival, Cellular Development
Network 11 Cellular Movement, Cell Morphology, Cellular Assembly and Organization
Network 12 Cancer, Connective Tissue Disorders, Developmental Disorder
Network 13 Developmental Disorder, Hereditary Disorder, Metabolic Disease
Network 14 Cell-To-Cell Signaling and Interaction, Hematological System Development and Function, Immune Cell Trafficking
Network 15 Cellular Movement, Hematological System Development and Function, Immune Cell Trafficking
Network 16 Hereditary Disorder, Neurological Disease, Organismal Injury and Abnormalities
Network 17 Gene Expression, Cancer, Cell Death and Survival
Network 18 Cell Death and Survival, Cellular Movement, Cancer
Network 19 Cell Death and Survival, Post-Translational Modification, Protein Degradation
Network 20 Cellular Growth and Proliferation, Cell Cycle, Cellular Development
Network 21 RNA Post-Transcriptional Modification, Developmental Disorder, Neurological Disease
Network 22 Gene Expression, Cell Cycle, Cell Morphology
Network 23 Cellular Function and Maintenance, Hematological System Development and Function, Lymphoid Tissue Structure and Development
Network 24 Cardiovascular System Development and Function, Cellular Assembly and Organization, DNA Replication, Recombination, and Repair
Network 25 Cellular Movement, Cancer, Organismal Injury and Abnormalities

Supplementary Figure 2. AXL is involved in a gene network regulating tumor immune response and M2 macrophages. a) Pathway analysis of genes co-expressed with AXL in triple negative breast cancer. Co-regulated genes were identified using the SEEK platform, and pathway analysis was performed with IPA. *P*-values for pathway enrichment were adjusted for multiple testing using the Benjamini-Hochberg correction. Immune pathways were indicated with an asterisk (*). b, c) The images represent the top networks identified with IPA software for the genes significantly co-expressed ($P < 0.01$) with the immune modules reflecting the polarization of M1 (b) or M2 (c) macrophages. As evidenced in (c), *AXL* was present in the signaling network associated with the M2-related module only.

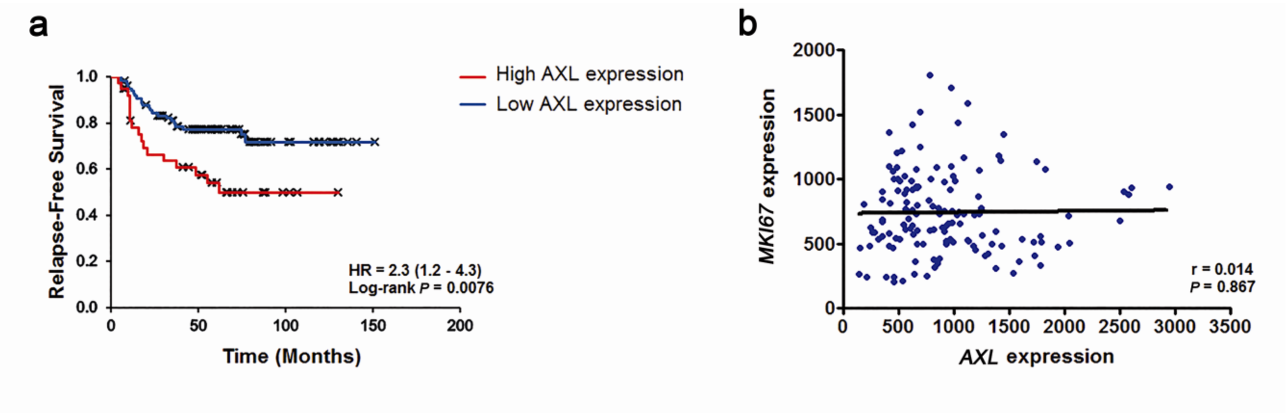

Supplementary Figure 3. *AXL* expression is predictive of relapse-free survival independently of proliferation in adjuvant chemotherapy treated triple-negative breast cancers. a) Kaplan-Meier analysis for relapse-free survival according to *AXL* expression in 137 TNBC treated with adjuvant chemotherapy. The median expression value of *AXL* was used as cutoff. b) Pearson's correlation analysis showing that *AXL* expression does not correlate with the expression of *MKI67* (r = 0.014).

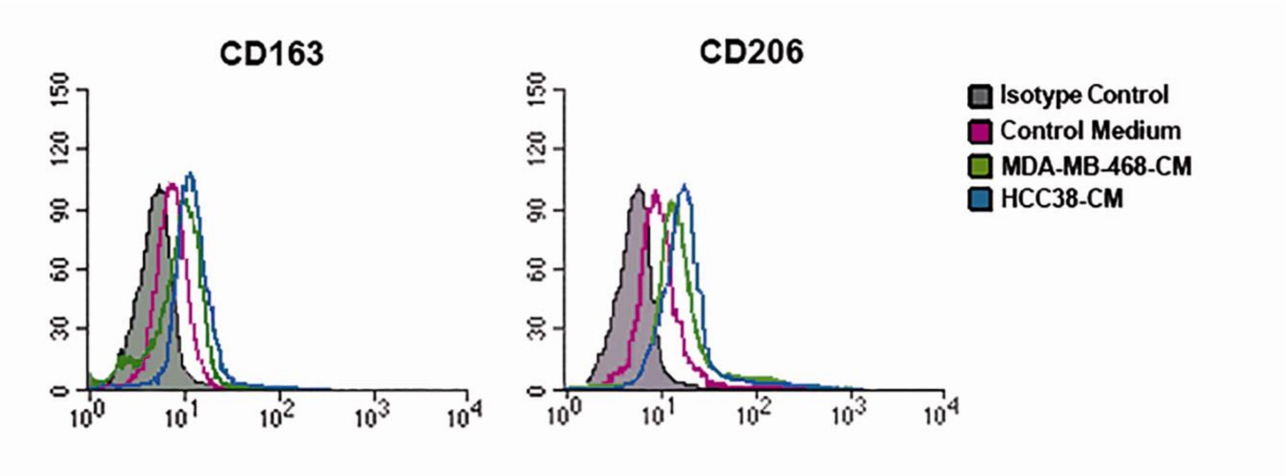

Supplementary Figure 4. Effects of *AXL*-overexpressing basal-like breast cancer cells on the polarization of M2 macrophages. Flow cytometric analysis of the M2 markers CD163 and CD206 in human monocytes cultured in the absence (pink) or presence of *AXL*-expressing basal-like HCC38-conditioned medium (CM) (blue), and *AXL*-negative basal-like MDA-MB-468-CM (green) for 6 days. Gray histograms represent staining with isotype controls. The histograms are representatives of five independent experiments.
